# Discovery of a First‐in‐Class Murine Double Minute 2‐Recruiting Positive Transcription Elongation Factor B PROTAC Degrader With Selective Antitumor Activity

**DOI:** 10.1002/mco2.70723

**Published:** 2026-04-09

**Authors:** Xian Guan, Long Xie, Hanjun Guo, Lin Ma, Jiawei Zhou, Lisong Luo, Hao Yang, Yuanfang Wu, Jiangyu Liu, Yue Wang, Xingze Huang, Jiyang Liu, Ying Zhang, Wenhao Chen, Ye Chen, Liang Xu, Xin Han

**Affiliations:** ^1^ Cancer Institute (Key Laboratory of Cancer Prevention and Intervention, China National Ministry of Education) of the Second Affiliated Hospital and Institute of Translational Medicine, Zhejiang University School of Medicine Hangzhou China; ^2^ Institute of Biochemistry, College of Life Sciences, Zhejiang University Hangzhou China; ^3^ Department of Surgical Oncology Children's Hospital Zhejiang University School of Medicine, National Clinical Research Center for Children and Adolescents' Health and Diseases Hangzhou China; ^4^ Pediatric Cancer Research Center, National Clinical Research Center For Children and Adolescents' Health and Diseases Hangzhou China; ^5^ Department of Orthopedic Surgery Children's Hospital Zhejiang University School of Medicine, National Clinical Research Center for Children and Adolescents' Health and Diseases Hangzhou China; ^6^ Cancer Center of Zhejiang University Hangzhou China; ^7^ Department of Radiation Oncology, Key Laboratory of Cancer Prevention and Intervention The Second Affiliated Hospital, Zhejiang University School of Medicine Hangzhou China

**Keywords:** anti‐cancer, cyclin T, cyclin‐dependent kinase 9, mouse double minute 2, positive transcription elongation factor B, proteolysis targeting chimera

## Abstract

The positive transcription elongation factor b (P‐TEFb) complex, composed of CDK9 and cyclin T isoforms (T1, T2a and T2b), is critical for gene transcription, positioning CDK9 as a very promising oncology target. However, the development of selective and clinically effective small‐molecule CDK9 inhibitors has proven challenging. To overcome this limitation, we designed a series of highly efficient and selective P‐TEFb degraders by conjugating the CDK9 inhibitor SNS032 with the mouse double minute 2 (MDM2) ligand RG7388. Our lead compound, **13** (dCDK9‐010), recruits the MDM2 E3 ligase to induce proteasome‐dependent degradation of CDK9 and all cyclin T isoforms across diverse cancer models. dCDK9‐010 potently inhibits RNA polymerase II carboxy‐terminal repeat domain phosphorylation and blocks MDM2‐mediated p53 degradation, resulting in concurrent p53 pathway activation. This dual mechanism drives selective cytotoxicity in *TP53* wild‐type cancer cells, sparing *TP53*‐mutant or nonmalignant cells. In murine xenograft models of lung cancer and Ewing sarcoma, intravenous dCDK9‐010 administration significantly inhibited tumor growth while demonstrating an excellent safety profile. Collectively, this study establishes dCDK9‐010 as a first‐in‐class, selective MDM2‐recruiting P‐TEFb degrader. By enabling the elimination of the entire P‐TEFb complex, this MDM2‐recruiting degradation strategy expands the toolkit for targeting CDK9 and represents a promising new therapeutic paradigm for *TP53* wild‐type cancers.

## Graphical abstract

This study reports the development of dCDK9‐010, a first‐in‐class, selective MDM2‐recruiting CDK9/cyclin T PROTAC degrader with compelling in vivo antitumor efficacy and safety profile. This degrader potently inhibits RNA polymerase II phosphorylation and interferences with MDM2‐mediated p53 degradation, resulting in concurrent transcription disruption and p53 pathway activation. MDM2‐recruiting CDK9/cyclin T PROTACs represent promising targeted therapeutic agents for MDM2&TP53‐proficient malignancies.

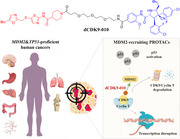

## 1 Introduction

CDK9 associates with its regulatory cyclin partners (i.e., Cyclin T1, Cyclin T2a, and Cyclin T2b, hereafter referred to as Cyclin T) to form functional positive transcription elongation factor B (P‐TEFb) complex mediating gene transcription and transcription‐coupled RNA processing via Ser2 phosphorylation of YSPTSPS hepta‐repeats in the carboxy‐terminal domain (CTD) of DNA‐directed RNA polymerase II (RNA Pol II) subunit Rpb1 [1, 2, 3, 4, 5]. CDK9/cyclin T complex critically potentiates transcription elongation and mRNA processing, thereby promoting cell proliferation and driving the occurrence and development of cancer [6, 7, 8]. Consequently, CDK9 represents a promising target for antitumor drug discovery [9, 10].

Notwithstanding this potential, the clinical translation of conventional CDK9 inhibitors has encountered obstacles, primarily due to suboptimal selectivity and dose‐limiting toxicities [11, 12]. A principal reason for this challenge lies in the high conservation of the ATP‐binding pocket across the kinome, making it difficult to achieve specificity for CDK9 over other CDK family members and unrelated kinases. In this context, PROTAC technology [13, 14, 15, 16, 17, 18] provides a new strategy to target CDK9 and enhance its specificity and safety. Unlike inhibitors that occupy the ATP‐binding site, PROTACs are heterobifunctional molecules that catalytically induce the ubiquitination and degradation of the target protein via the ubiquitin‐proteasome system. CDK9 PROTAC degraders, as an emerging therapeutic strategy, have the potential to become important tools in future cancer treatment [19, 20]. Currently, various CDK9 PROTAC degraders, such as THAL‐SNS032 and B03, have been reported, all of which exhibit submicromolar‐level CDK9‐selective degradation and anti‐proliferative activity in cancer cells [21, 22, 23, 24, 25].

However, these pioneer degraders are not without limitations. Unfortunately, these molecules have low bioavailability in vivo and lack cancer cell‐specific targeting. This narrow therapeutic index could hinder their clinical application. A significant concern is that these molecules were often designed using first‐generation Cereblon (CRBN) ligands (e.g., thalidomide), which can lead to off‐target effects on G1 to S phase transition (GSPT) 1/2 and Ikaros family zinc finger protein (IKZF) 1/3 [26, 27], thereby causing unwanted toxic side effects. This collateral degradation of non‐target proteins, a phenomenon inherent to the molecular glue mechanism of these E3 ligase ligands, presents a significant safety consideration for drug development. Furthermore, the reliance on a limited set of E3 ligases, primarily CRBN and VHL [28, 29, 30, 31], may restrict the degradation efficiency and tissue distribution of resulting PROTACs. Therefore, it remains crucial to develop novel, efficient, and bioavailable CDK9 PROTAC drugs with improved safety profiles, prompting the exploration of alternative E3 ligase ligands and degraders design.

Exploring alternative E3 ligases is a key strategy for innovating the PROTAC landscape. Mouse double minute 2 (MDM2) is an E3 ubiquitin ligase, whose main function is to promote the ubiquitination and degradation of the prominent tumor suppressor protein p53 in cells [32, 33, 34]. It is well established that the MDM2‐p53 pathway plays a central role in cancer biology, regulating critical processes such as cell cycle control, genome stability, cell death, metabolic reprogramming, and immune modulation [35, 36]. In some cancers, MDM2 is overexpressed, persistently degrading intracellular wild‐type p53 via the ubiquitin‐proteasome pathway [37]. This thereby suppresses p53 expression levels and activity, further promoting abnormal cell proliferation and cancer formation. This mechanism plays a significant role in cancer development. Given the critical interaction between MDM2 and p53, some oncological drugs are designed to inhibit the binding of MDM2 to p53, thereby preserving the antitumor function of p53 and becoming a promising strategy for cancer treatment [38, 39, 40]. By leveraging MDM2's E3 ligase activity, PROTACs incorporating MDM2 ligands enable targeted degradation of disease‐driving proteins. This strategy precisely modulates protein stability to disrupt pathological signaling pathways, representing a novel therapeutic paradigm for intercepting oncogenic processes [41, 42, 43, 44, 45, 46]. Compound A1874 is the first PROTAC molecule that employs MDM2 to degrade the BRD4 protein. A1874 can effectively degrade BRD4 while stabilizing the p53 protein, thus exerting a dual effect to inhibit the proliferation of cancer cells with high MDM2 expression both in vitro and in vivo [47]. However, different substrate proteins display significant variations in their sensitivity to the MDM2‐dependent degradation process [48]. Consequently, the design of MDM2‐based PROTACs targeting specific proteins remains fraught with unknowns and challenges.

To date, no MDM2‐dependent PROTAC degraders targeting either CDK9 or cyclin T have been reported. In this work, we have designed and synthesized, for the first time, degraders targeting CDK9/cyclin T complex (P‐TEFb) based on the MDM2 E3 ligase ligand RG7388 for use in antitumor drug discovery and development.

## 2 Results

### 2.1 Design and Synthesis of New CDK9/Cyclin T Degraders With MDM2 E3 Ligands

**FIGURE 1:**
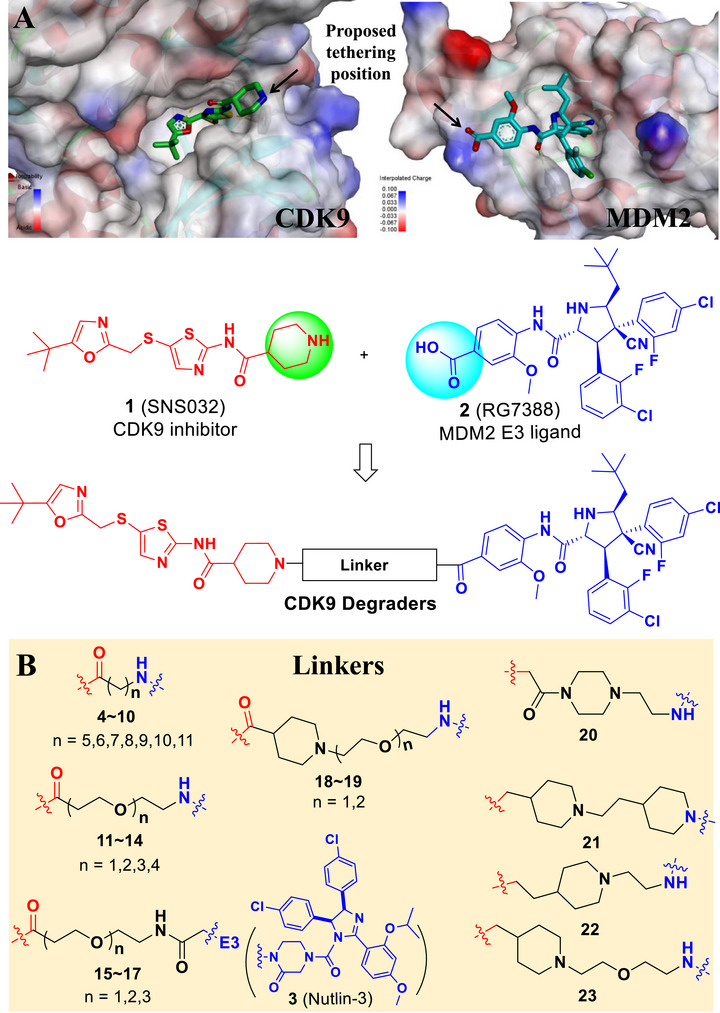
Design and optimization of CDK9 degraders. (A) Docking model of CDK9 inhibitor SNS032 to CDK9 enzyme (Protein Data Bank: 8K5R); docking model of MDM2 ligand RG7388 to MDM2 E3 ubiquitin ligase (Protein Data Bank: 4JSC); structures of CDK9 inhibitor, MDM2 ligand, and degraders. (B) Linkers used for CDK9 degraders in this manuscript.

In our endeavor to develop novel degraders for the CDK9/cyclin T complex, we adopted a PROTAC strategy that conjugates the CDK9 inhibitor SNS032 with MDM2 E3 ligands, namely, RG7388 and Nutlin‐3 (Figure 1A). Molecular docking analysis of SNS032 bound to CDK9 (Protein Data Bank: 8K5R) indicated that the piperidine ring, projecting into the solvent‐accessible region, serves a suitable attachment site for linker connection in PROTAC design. Similarly, in the docking results of MDM2 protein in a complex with a potent MDM2 E3 ligand RG7388, the benzoic acid group is exposed to solvent, making this site suitable for the design of potential MDM2‐based PROTACs.

We then designed and synthesized compounds **4**‐**23** using the CDK9 inhibitor SNS032 tethering to either RG7388 or Nutlin‐3 with linear/flexible and rigid linkers (Figure 1B, Table 1). The CDK9 degradation efficiency of these compounds was examined in the non‐small cell lung cancer cell line NCI‐H226 and an Ewing sarcoma cell line TC‐32, both of which are *TP53* wild type (Figure 1A,B, Table 1, Figures S1 and S2). Initially, we synthesized compounds **4**–**10** containing a linker with 5–11 methylene groups. However, none of these compounds exhibited significant degradation of CDK9 in both NCI‐H226 and TC‐32 cell lines. We next investigated the chemical composition of the linker. Interestingly, when we introduced oxygen atoms into the linker to synthesize compounds **11**–**14**, significant CDK9 protein degradation activity was observed in both NCI‐H226 and TC‐32 cell lines, especially for compounds **12** (dCDK9‐009) and **13** (dCDK9‐010). Our results show that compound **12** has a DC_50_ value of 0.5 ± 0.1 µM in TC‐32 cell line and compound **13** has DC_50_ values of 0.5 ± 0.1 µM and 1.4 ± 0.2 µM in NCI‐H226 and TC‐32 cells, respectively. When we replaced RG7388 with another MDM2 E3 ligand Nutlin‐3, compounds **15‐** **17** almost lost their degradation activity against the CDK9 protein (Figure 1A,B, Table 1, Figures S1 and S2). We further investigated the chemical composition of the linker by introducing rigid rings to synthesize compounds **18**–**23**. Our results show that the introduction of rigid rings is beneficial for enhancing the degradation activity of CDK9, such as compounds **18**–**19**, especially compound **23**. Taken together, compounds **11–13** and **23** exhibited the highest efficiency in depleting CDK9 protein in both NCI‐H226 and TC‐32 cells.

**TABLE 1:** Degradation and cell growth inhibition in NCI‐H226 and TC‐32 cells treated with CDK9 degraders^a^.

No.	Compound	DC_50_ (µM)		IC_50_ (µM)	
		NCI‐H226	TC‐32	NCI‐H226	TC‐32
**4**	dCDK9‐001	> 2	> 2	2.5 ± 0.1	4.2 ± 0.1
**5**	dCDK9‐002	> 2	> 2	9.9 ± 0.3	>10
**6**	dCDK9‐003	> 2	> 2	>10	>10
**7**	dCDK9‐004	> 2	> 2	>10	>10
**8**	dCDK9‐005	> 2	> 2	>10	8.0 ± 0.1
**9**	dCDK9‐006	> 2	> 2	>10	>10
**10**	dCDK9‐007	> 2	> 2	>10	>10
**11**	dCDK9‐008	0.5 ± 0.2	1.8 ± 0.2	0.3 ± 0.03	1.0 ± 0.3
**12**	dCDK9‐009	2.0 ± 0.9	0.5 ± 0.1	0.3 ± 0.03	0.5 ± 0.2
**13**	dCDK9‐010	0.5 ± 0.1	1.4 ± 0.2	0.2 ± 0.02	0.4 ± 0.09
**14**	dCDK9‐012	> 2	> 2	0.2 ± 0.01	0.4 ± 0.02
**15**	dCDK9‐015	> 2	> 2	2.5 ± 0.2	1.7 ± 0.2
**16**	dCDK9‐016	> 2	> 2	4.5 ± 0.4	2.2 ± 0.09
**17**	dCDK9‐017	> 2	> 2	2.7 ± 0.001	1.9 ± 0.2
**18**	dCDK9‐019	0.8 ± 0.1	> 2	0.5 ± 0.01	0.3 ± 0.01
**19**	dCDK9‐020	0.8 ± 0.2	> 2	0.3 ± 0.01	0.2 ± 0.01
**20**	dCDK9‐021	> 2	> 2	0.1 ± 0.01	0.3 ± 0.04
**21**	dCDK9‐022	> 2	> 2	0.4 ± 0.04	0.5 ± 0.1
**22**	dCDK9‐023	> 2	> 2	0.3 ± 0.02	0.3 ± 0.02
**23**	dCDK9‐027	0.4 ± 0.1	1.7 ± 0.3	0.4 ± 0.05	0.3 ± 0.01

^a^ All data are the average of three independent experiments.

### 2.2 Evaluation of Those Potent CDK9/Cyclin T Degraders for Growth Inhibition in Both NCI‐H226 and TC‐32 Cells

In order to comprehensively assess the in vitro activity of these CDK9/cyclin T degraders, we conducted cell growth inhibition studies using compounds **4–23** in both NCI‐H226 and TC‐32 cells (Figure 1A,B, Table 1, Figures S3 and S4). Consistent with their potent CDK9 degradation, compounds **11–13** and **23** exhibited sub‐micromolar anti‐proliferative activity (IC_50_: 0.2–1.0 µM) across these two cancer cell models.

### 2.3 dCDK9‐009 and dCDK9‐010 Selectively Induced CDK9/Cyclin T Degradation

**FIGURE 2:**
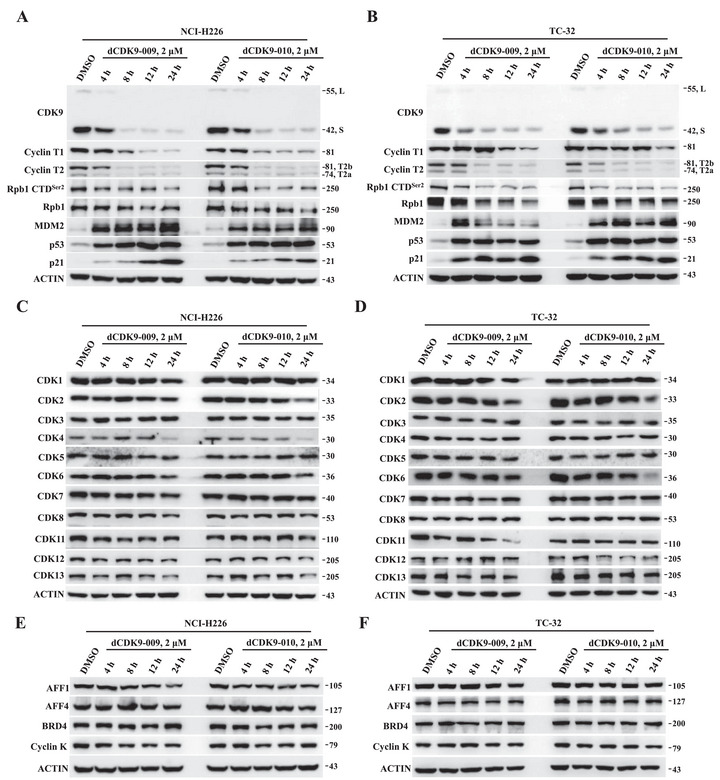
Temporal effect of **12** (dCDK9‐009) and **13** (dCDK9‐010) on intracellular protein homeostasis. (A and B) Protein levels of CDK9 (the predominant 42‐kD isoform), Cyclin T, and other putative target proteins in NCI‐H226 (A) and TC‐32 (B) cells upon time‐course treatment with dCDK9‐009 and dCDK9‐010 (2 µM). (C–F) Protein levels of various CDKs and CDK9‐interacting proteins in NCI‐H226 and TC‐32 cells upon time‐course treatment with dCDK9‐009 and dCDK9‐010 (2 µM).

Next, we assessed the degradation kinetics of compound **12** (dCDK9‐009) and compound **13** (dCDK9‐010) in NCI‐H226 and TC‐32 cells, and the western blotting results are shown in Figure 2. Compounds **12** and **13** rapidly reduced protein levels of CDK9 (55‐kD CDK9‐L and 42‐kD CDK9‐S) and Cyclin T2 within 8 h, with Cyclin T1 depletion evident between 8 and 24 h post‐treatment at a concentration of 2 µM (Figure 2A,B). For compound **12** (dCDK9‐009), its maximal levels of degradation (*D*_max_) of CDK9‐S, CDK9‐L, Cyclin T1, Cyclin T2b, and Cyclin T2a achieved 95%, > 95%, 93%, 77%, and 80% in NCI‐H226 cells, and 83%, > 95%, 67%, 88%, and 90% in TC‐32 cells, respectively. In the case of compound **13** (dCDK9‐010), its maximal levels of degradation (*D*_max_) of CDK9‐S, CDK9‐L, Cyclin T1, Cyclin T2b, and Cyclin T2a were > 95%, > 95%, 75%, 70%, and 81% in NCI‐H226 cells, and 84%, > 95%, 61%, 95%, and 92% in TC‐32 cells, respectively. In parallel to CDK9 and Cyclin T1/T2 depletion, phospho‐Rpb1 (CTD Ser2) was reduced, while MDM2, p53, and p21 levels were strongly elevated after 4h treatment. Remarkably, further examining the effects of compound **12** (dCDK9‐009) and compound **13** (dCDK9‐010) on a panel of CDK proteins and CDK9‐interacting proteins demonstrated no acute suppression of other CDKs, BRD4, Cyclin K, AFF1, or AFF4 within 12 h (Figure 2C–F). However, a moderate, collateral reduction in CDK2 protein levels was consistently observed after 24‐h treatment in both cell lines (Figure 2C,D). Real‐time quantitative polymerase chain reaction (qPCR) analysis indicated that both compounds significantly reduced *CDK2* mRNA levels as early as 8 h post‐treatment, prior to protein decline (Figure S5A,B). This pattern extended to other CDKs (*CDK4*, *CDK6*, *CDK11*, *CDK12*, and *CDK13*) whose mRNA downregulation also preceded their protein reduction in NCI‐H226 cells (Figure S5C). Hence, the collateral downregulation of multiple CDK proteins is not a direct off‐target effect, but a secondary transcriptional consequence caused by P‐TEFb degradation. Together, kinetic profiling reveals that these PROTACs induce rapid, concerted intracellular responses in target cells through CDK9/cyclin T degradation, RNA Pol II inhibition, and p53 stabilization/activation.

**FIGURE 3:**
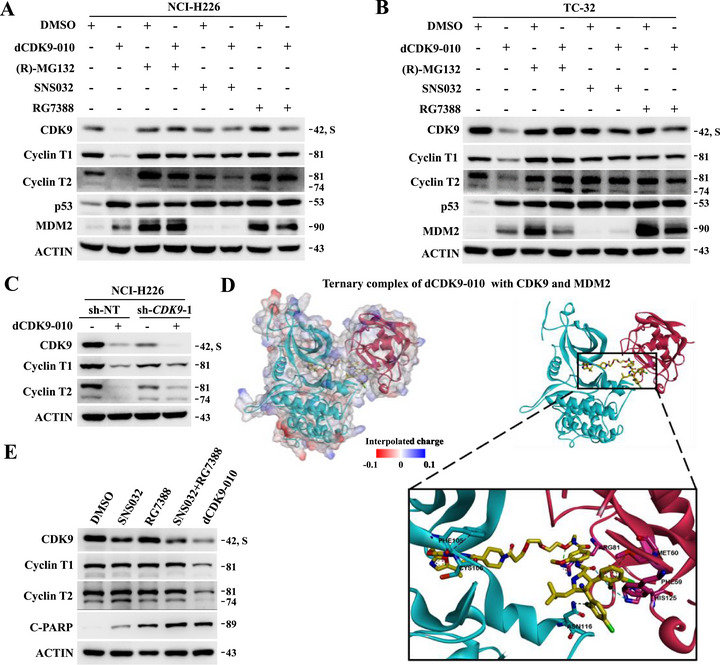
Mechanistic investigation of CDK9/Cyclin T degradation induced by **13** (dCDK9‐010) in NCI‐H226 and TC‐32 cells. (A and B) Effects of (R)‐MG132 (10 µM), SNS032 (20 µM), and RG7388 (20 µM) pretreatments on dCDK9‐010's CDK9/Cyclin T degrading activity in TC‐32 and NCI‐H226 cells. Cells were pretreated with indicated compounds for 2 h, followed by 8‐h treatment with dCDK9‐010 at 2 µM. (C) Effect of shRNA‐mediated *CDK9* knockdown on dCDK9‐010‐mediated CDK9/Cyclin T degradation. Indicated NCI‐H226 cells were treated with dCDK9‐010 at 500 nM for 8 h. (D) Predicted docking model of the ternary complex: CDK9 enzyme (Protein Data Bank: 8K5R, cyan)‐dCDK9‐010 (yellow)‐MDM2 E3 ubiquitin ligase (Protein Data Bank: 4JSC, red). (E) Effects of SNS032, RG7388, SNS032/RG7388 combo, and dCDK9‐010 on CDK9, Cyclin T, and c‐PARP levels. TC‐32 cells were treated with each compound at 500 nM for 24 h

We investigated the mechanism of CDK9/Cyclin T degradation induced by compound **13** (dCDK9‐010) in NCI‐H226 and TC‐32 cells. Supported by consistent observations in both cells, CDK9 and cyclin T degradation caused by dCDK9‐010 could be effectively reversed by pretreatment with the CDK9 inhibitor SNS032, the MDM2 E3 ligand RG7388, and the proteasome inhibitor (R)‐MG132 (Figure 3A,B). MDM2 protein was markedly induced by dCDK9‐010, (*R*)‐MG132 and RG7388, but was barely affected by SNS032. Intriguingly, silencing of endogenous *CDK9* attenuated the activity of dCDK9‐010 to degrade Cyclin T1/T2 (Figure 3C), suggesting that CDK9 serves as the molecular bridge to facilitate the MDM2‐mediated Cyclin T degradation in the presence of compound **13** (dCDK9‐010). Such cyclin T degradation activity is unique to the MDM2‐recruiting compounds, for example, compounds **12** (dCDK9‐009) and **13** (dCDK9‐010), as existing CRBN‐recruiting CDK9 PROTAC degraders made of the same warhead merely affected cyclin T levels (Figure S6).

To further verify whether compound **13** (dCDK9‐010) could form a stable ternary complex with CDK9/cyclin T and MDM2, we conducted molecular docking simulation analysis (Figure 3D). Compound dCDK9‐010 forms hydrogen bond and π‐π interactions with multiple amino acid residues in the protein interface, specifically with PHE‐105 from CDK9/cyclin T and ARG‐81/ASN‐116 from MDM2. The formation of hydrogen bonds and π‐π interactions enable proteins to bind tightly with compound dCDK9‐010. In addition, it could also engage in extensive hydrophobic interactions with multiple residues (PHE‐105, CYS‐106, PHE‐59, MET‐60, ARG‐81, ASN‐116, HIS‐125, etc.), contributing substantial van der Waals stabilization effects to the protein–ligand complex. Binding affinity from the bio‐layer interferometry (BLI) assay also indicated that the observed differences in degradation efficacy are influenced by both the binding affinity (Figure S7) and the formation of the ternary complex.

**FIGURE 4:**
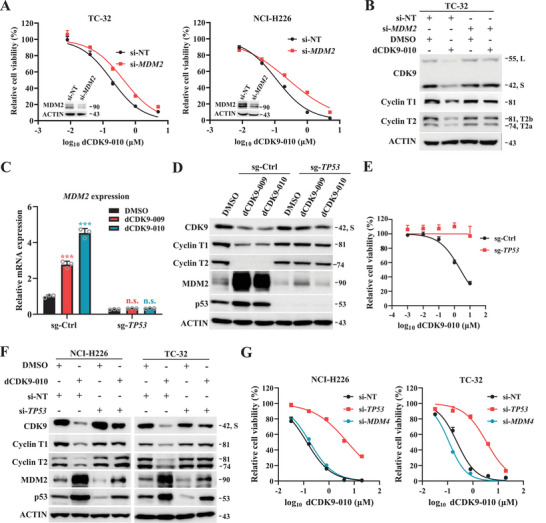
MDM2‐ and p53‐dependent activities of MDM2‐recruiting P‐TEFb PROTAC degraders. (A) Effects of siRNA‐mediated *MDM2* knockdown on cellular sensitivity to dCDK9‐010. Data were presented as mean ± standard deviation (SD); *n* = 3. (B) Effects of *MDM2* knockdown on dCDK9‐010‐mediated CDK9/Cyclin T degradation. TC‐32 cells were transfected with indicated siRNAs for 48 h, followed by treatment with 2 µM dCDK9‐010 for 8 h. (C) *MDM2* mRNA levels in wild type versus *TP53*‐knockout U87 cells, with or without compound treatment (2 µM, 8 h). Data were presented as mean ± SD (*n* = 3); ***p < 0.001 based on one‐way analysis of variance (ANOVA); n.s., no significance. (D) Immunoblot analysis of P‐TEFb components, p53, and MDM2 in wild‐type and *TP53*‐knockout isogenic U87 cells after compound treatment (2 µM, 8 h). (E) Viability of isogenic U87 cells treated with dCDK9‐010. Data were presented as mean ± SD (*n* = 3). (F) Immunoblot analysis of P‐TEFb components, p53, and MDM2 in NCI‐H226 and TC‐32 cells after siRNA‐mediated *TP53* silencing and dCDK9‐010 treatment (2 µM, 8 h). (G) Effects of *TP53* or *MDM4* knockdown by siRNA relative to control on dCDK9‐010 sensitivity in NCI‐H226 and TC‐32 cells. Data were presented as mean ± SD (*n* = 3).

In addition, we compared the effects of RG7388, SNS032, and compound **13** (dCDK9‐010) as single agents, as well as the RG7388/SNS032 combination, on key downstream proteins in TC‐32 cells. dCDK9‐010 uniquely induced CDK9/cyclin T degradation among all treatments; it also elicited cleaved poly (ADP‐ribose) polymerase (c‐PARP) level higher than that achieved with either single‐agent treatment (RG7388 or SNS032), yet slightly lower than the level observed with their combination (Figure 3E). Remarkably, siRNA‐mediated silencing of endogenous *MDM2* rendered NCI‐H226 and TC‐32 cells less sensitive to dCDK9‐010 (Figure 4A). In parallel, MDM2‐silenced TC‐32 cells also showed reduced response to dCDK9‐010 in terms of CDK9/cyclin T degradation (Figure 4B). Collectively, these data unequivocally demonstrate that dCDK9‐010 functions as a genuine MDM2‐dependent PROTAC degrader of the CDK9/cyclin T complex.

MDM2 is a well‐established target of p53. Consistent with this regulation, clustered regularly interspaced short palindromic repeats (CRISPR)/CRISPR‐associated protein 9 (Cas9)‐mediated knockout of *TP53* in *TP53* wild type cells (e.g., U87) significantly reduced both basal and treatment‐induced MDM2 expression at the mRNA and protein levels (Figure 4C,D). Resembling the effect of genetic *MDM2* depletion, the resultant MDM2 insufficiency in *TP53*‐knockout cells led to a complete resistance to dCDK9‐010, impairing both its anti‐proliferative efficacy and the on‐target degradation of P‐TEFb (Figure 4D,E). To generalize this finding, siRNA‐mediated acute depletion of endogenous *TP53* was performed in NCI‐H226 and TC‐32 cells. Perturbation of wild‐type p53 robustly decreased cellular sensitivity to dCDK9‐010 (Figure 4F,G), further corroborating the p53‐dependency. In contrast, silencing of MDM4, a closely related MDM2 paralog and interactor, did not confer survival advantage upon dCDK9‐010 treatment (Figure 4G). Together, these genetic experiments demonstrate that the on‐target activity of dCDK9‐010 is critically dependent on functional p53.

**FIGURE 5:**
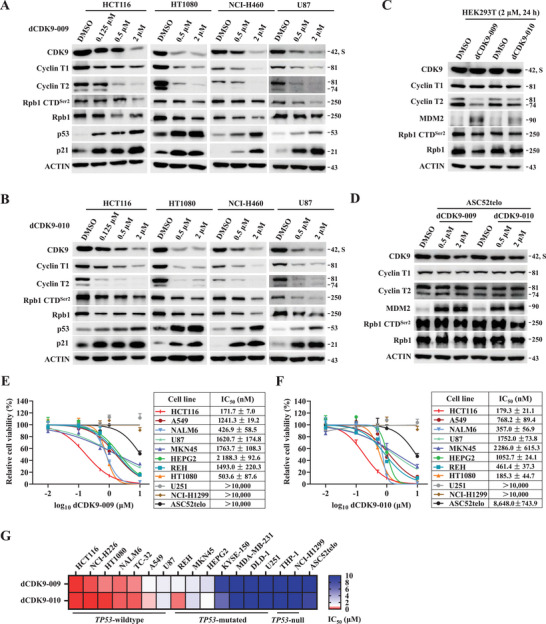
Selective activities of compounds **12** (dCDK9‐009) and **13** (dCDK9‐010) against *TP53* wild‐type cancer cells. (A and B) Examination of dose‐dependent impacts of dCDK9‐009 and dCDK9‐010 in *TP53* wild‐type HCT116, HT1080, NCI‐H460, and U87. Cells were treated with increasing concentrations of indicated compounds for 24 h. (C and D) Examination of impacts of dCDK9‐009 and dCDK9‐010 in non‐malignant HEK293T and mesenchymal stem cell line ASC52telo. Cells were treated with increasing concentrations of indicated compounds for 24 h. (E and F) Effect of dCDK9‐009 and dCDK9‐010 treatment on cellular viabilities of indicated cancer cell lines, as well as ASC52telo. IC_50_ was presented as mean ± SD with three independent replicates. (G) Heatmap summarizing the IC_50_ values of dCDK9‐009 and dCDK9‐010 across human cell lines tested in this study (related to E and F, Figures S3, S4, and S8).

We next investigated the ability of compounds **12** (dCDK9‐009) and **13** (dCDK9‐010) to degrade CDK9 in additional cancer cell lines of diverse tissue origins. Mirroring the findings in NCI‐H226 and TC‐32 (Figure 2A), both dCDK9‐009 and dCDK9‐010 depleted significantly endogenous CDK9 and cyclin T at 500 nM in NCI‐H460 non‐small cell lung cancer cells, U87 glioblastoma cells, HCT116 colon cancer cells, and HT1080 fibrosarcoma cells (Figure 5A,B). In contrast, these compounds exhibited minimal to moderate effects on CDK9 and Cyclin T1 levels in p53‐compromised HEK293T cells and non‐transformed hTERT‐immortalized ASC52telo human mesenchymal stem cells, except for a notable downregulation of Cyclin T2 in HEK293T cells (Figure 5C,D). Hence, these MDM2‐dependent CDK9/Cyclin T PROTACs demonstrate selective target degradation in *TP53* wild‐type cancer cells while sparing p53‐compromised or non‐transformed counterparts.

The potent degradation of CDK9/Cyclin T complexes by compounds **12** (dCDK9‐009) and **13** (dCDK9‐010) across diverse cancer models motivated us to further evaluate their anti‐proliferative effects in an expanded cell line panel (Figure 5E–G and Figure S8). Overall, compounds dCDK9‐009 and dCDK9‐010 exhibited comparable anti‐proliferative potency across diverse cancer models. Both compounds achieved sub‐micromolar to low micromolar IC_50_ values in *TP53* wild‐type lines including HCT116 (colon cancer), HT1080 (fibrosarcoma), NALM6 (B‐cell acute lymphoblastic leukemia), A549 (non‐small cell lung cancer), and U87 (glioblastoma), while showing poor efficacy in *TP53*‐null NCI‐H1299 (non‐small cell lung cancer, IC_50_: >10 µM), and THP‐1 (acute monocytic leukemia, IC_50_: >10 µM), and in *TP53*‐mutated U251 (glioblastoma, IC_50_: >10 µM), DLD‐1 (colon cancer, IC_50_: >10 µM), and MDA‐MB‐231 (breast cancer, IC_50_: >10 µM). Other *TP53*‐mutated cancer cell lines tested in this study, namely, REH (acute non‐B non‐T lymphocytic leukemia), HEPG2 (liver cancer), MKN45 (gastric cancer), and KYSE‐150 (esophageal squamous cell carcinoma), retained partial sensitivity to these compounds, implying that the cellular response to these MDM2‐recruiting P‐TEFb PROTAC degraders may be influenced by *TP53* mutation type and zygosity (Figure 5G). Importantly, these compounds exhibited minimal toxicity in non‐malignant ASC52telo mesenchymal stem cells (IC_50_: >10 µM and 8.65 µM, respectively), indicative of a favorable therapeutic index.

### 2.4 dCDK9‐009 and dCDK9‐010 Induced Apoptosis in Cancer Cells

**FIGURE 6:**
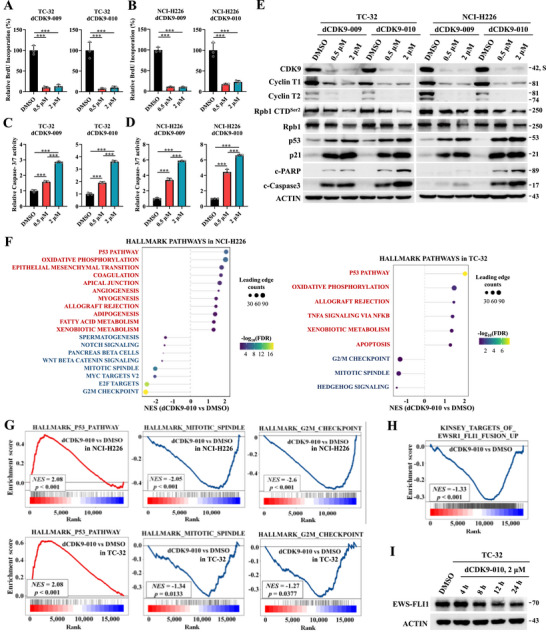
Anti‐proliferative activities of CDK9/Cyclin T degraders **12** (dCDK9‐009) and **13** (dCDK9‐010). (A and B) Effect of compounds dCDK9‐009 and dCDK9‐010 treatments (24 h) on BrdU incorporation by TC‐32 and NCI‐H226 cells. Data were presented as mean ± SD; ***p < 0.001 based on one‐way ANOVA. (C and D) Effect of compounds dCDK9‐009 and dCDK9‐010 treatments (24 h) on Caspase‐3/7 activity in TC‐32 and NCI‐H226 cells. Data were presented as mean ± SD; ***p < 0.001 based on one‐way ANOVA. (E) Effect of compounds dCDK9‐009 and dCDK9‐010 treatments (24 h) on intracellular levels of CDK9, Cyclin T, p53, p21, and apoptotic markers in TC‐32 and NCI‐H226 cells. (F) Differentially enriched hallmark pathways in NCI‐H226 and TC‐32 cells treated with dCDK9‐010 (500 nM, 8 h). (G) Representative biological pathways affected by dCDK9‐010 treatment (500 nM, 8 h) in NCI‐H226 and TC‐32 cells. FDR, false discovery rate; NES, normalized enrichment score. (H) Negative enrichment of EWS‐FLI1 targets in TC‐32 cells treated with dCDK9‐010 (500 nM, 8 h). (I) Protein level of EWS‐FLI1 in TC‐32 cells upon time‐course treatment with dCDK9‐010 (2 µM).

In line with efficient CDK9/Cyclin T degradation and p53 induction, compounds **12** (dCDK9‐009) and **13** (dCDK9‐010) profoundly suppressed cellular DNA replication, as measured by bromodeoxyuridine (BrdU) incorporation in both NCI‐H226 and TC‐32 cells at a dose of 500 nM (Figure 6A,B). Concurrently, these compounds significantly activated Caspase‐3/7 (Figure 6C,D) and elevated the levels of cleaved Caspase‐3 and c‐PARP in a dose‐dependent manner (Figure 6E). Hence, our newly designed CDK9/Cyclin T degraders are capable of inhibiting cell proliferation and inducing apoptosis.

As suggested by the higher levels of cleaved Caspase‐3 and c‐PARP, compound **13** (dCDK9‐010) showed a slightly better efficacy in apoptotic induction than **12** (dCDK9‐009). We then selected compound dCDK9‐010 as a lead for further biological and pharmacokinetic investigation. To explore the impact of MDM2‐dependent CDK9/Cyclin T degraders on global gene expression, we performed an RNA‐seq analysis in NCI‐H226 and TC‐32 cells treated with either dimethylsulfoxide (DMSO) or dCDK9‐010 (500 nM, 8 h). Gene set enrichment analysis (GSEA) revealed that dCDK9‐010 induced significant upregulation of p53 pathways, concomitant with repression of mitotic spindle and G2/M checkpoint pathway‐related genes in both cell lines (Figure 6F,G). Intriguingly, dCDK9‐010 significantly attenuated the EWS‐FLI1‐dependent transcriptional network (specifically, “KINSEY targets of EWSR‐FLI1 fusion UP” dataset; false discovery rate = 0.0046, net enrichment score = −1.33) in the Ewing sarcoma cell line TC‐32, without affecting the protein level of EWS‐FLI1 (Figure 6 H,I and Figure S9). Given that EWS‐FLI1 is a fusion transcription factor driving Ewing sarcoma development, these data suggest dCDK9‐010 can selectively disrupt transcriptional addiction to EWS‐FLI1 protein in this disease. Together, dCDK9‐010 appears capable of impairing both the disease‐specific gene expression program and general oncogenic pathways.

### 2.5 dCDK9‐010 Significantly Inhibited Tumor Growth in Xenograft Models

**FIGURE 7:**
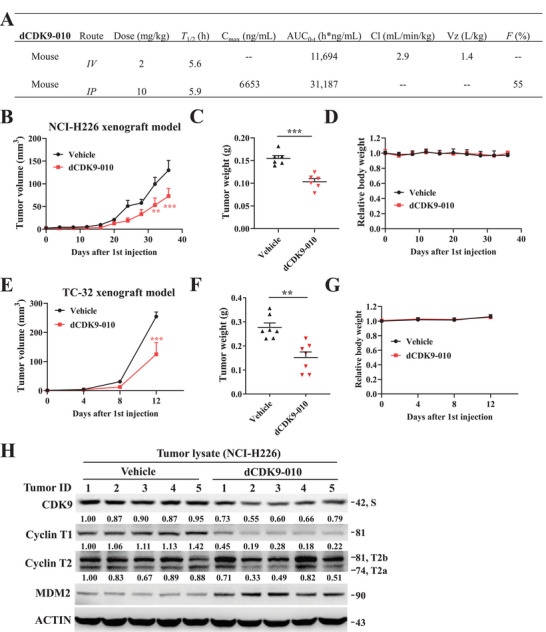
Antitumor activity of **13** (dCDK9‐010) in the NCI‐H226 and TC‐32 xenograft models in immunocompromised mice. (A) In vivo PK profile of dCDK9‐010 in mice (*n* = 3). AUC, area under the curve. (B) Changes of NCI‐H226 xenograft volume during treatment. Data were presented as mean ± standard error of the mean (SEM; **p < 0.01; ***p < 0.001; two‐tailed t‐test (*n* = 6)). (C) NCI‐H226 xenograft weight at the endpoint of experiment. Data were presented as mean ± SEM; ***p < 0.001; two‐tailed t‐test (*n* = 6). (D) Body weight of NCI‐H226 xenograft‐bearing mice during the drug treatment. Data were presented as mean ± SEM (*n* = 3). (E) Changes of TC‐32 xenograft volume during treatment. Data were presented as mean ± SEM; ***p < 0.001; two‐tailed *t*‐test (*n* = 7). (F) TC‐32 xenograft weight at the endpoint of experiment. Data were presented as mean ± SEM; **p < 0.01; two‐tailed *t*‐test (*n* = 7). (G) Body weight of TC‐32 xenograft‐bearing mice during the drug treatment. Data were presented as mean ± SEM (*n* = 4). (H) Western blotting analysis of CDK9 and Cyclin T proteins in lysates from NCI‐H226 xenograft tumors harvested at the endpoint of experiment. CDK9‐S, Cyclin T1, and Cyclin T2a levels were quantified.

*Pharmacokinetic studies of compound 13 (dCDK9‐010) in mice*: We first evaluated the pharmacokinetics (PK) of CDK9 degrader dCDK9‐010 in mice with both intravenous and intraperitoneal administration, obtaining the data summarized in Figure 7A. The PK data show that compound dCDK9‐010 achieves excellent overall PK profiles. Compound dCDK9‐010 has a low clearance (Cl) of 2.9 mL/min/kg and a moderate volume of distribution (*V*_z_) of 1.4 L/kg. Compound dCDK9‐010 has a moderate drug half‐life (*T*_1/2_) following intravenous and intraperitoneal administration, ranging from 5.5 to 6 h. Compound dCDK9‐010 achieves a maximum plasma concentration (*C*_max_) value of 6653 ng/mL following intravenous administration and AUC_0‐t_ values of 11,694 and 31,187 h·ng/mL following intravenous and intraperitoneal administration, respectively. The bioavailability of compound dCDK9‐010 following intraperitoneal administration is 55%.

*Antitumor activity of compound 13 (dCDK9‐010) in xenograft models*: Building upon its favorable selectivity and pharmacokinetic profile, we assessed the in vivo antitumor efficacy of compound **13** (dCDK9‐010) (Figure 7). Intravenous administration of dCDK9‐010 (20 mg/kg, every other day) significantly suppressed the growth of subcutaneous NCI‐H226 and TC‐32 xenografts in immunocompromised mice, as reflected by reduced tumor volume and weight compared to the vehicle control (Figure 7B–G). Remarkably, the treatment was well‐tolerated, with no significant body weight loss observed in the treated mice (Figure 7D,G). Tumor lysate analysis from NCI‐H226 models confirmed on‐target degradation of CDK9 and Cyclin T (preferentially Cyclin T1 and T2a) complex (Figure 7H), validating the mechanism of action in vivo.

## 3 Discussion

The MDM2‐p53 axis plays a critical role in tumorigenesis and the progression of cancer. Notably, the combination of MDM2 inhibitors and CDK9 inhibitors has demonstrated synergistic effects in cancer models including melanoma and sarcoma, motivating development of dual‐targeting agents. Leveraging this synergy, we designed a series of novel PROTACs using the MDM2 ligand RG7388 to specifically degrade CDK9 and Cyclin T proteins for antitumor drug development.

Recent advances in protein degradation, including CRBN‐recruiting PROTACs, hydrophobic tags, and autophagy‐tethering compound, have expanded the toolkit for therapeutic targeting of CDK9 [19, 23, 25, 49, 50, 51]. Nevertheless, a fundamental challenge in targeting this essential transcriptional kinase is the lack of cancer cell selectivity, which often results in dose‐limiting toxicity. Our strategy addresses this challenge by harnessing cancer‐specific E3 ligase MDM2 for P‐TEFb proteolysis, opening a new avenue for selective transcription‐targeted therapy. Notably, while hydrophobic tags and autophagy‐tethering compounds targeting CDK9 can reduce Cyclin T1 levels, existing CDK9‐targeting PROTACs (e.g., THAL‐SNS032 and dCDK9‐202) fail to do so. In contrast, our lead compound dCDK9‐010 induces the co‐depletion of CDK9 and Cyclin T, but not other CDK9‐interacting proteins (e.g., BRD4, Cyclin K, and AFF1/4). The degradation of Cyclin T is dependent on CDK9 that serves as the molecular bridge to MDM2. This mechanism is analogous to the bridged degradation of Cyclin D1 by a PROTAC degrader MS28, which requires CDK4/6 to recruit the von Hippel‐Lindau E3 ligase [52]. However, dCDK9‐010 exhibits a critical distinction: while MS28 achieves cyclin‐selective degradation, our compound induces the concomitant degradation of both the cyclin partner (Cyclin T) and the bridging kinase (CDK9) itself. This “self‐consuming bridge” mechanism ensures the complete and irreversible depletion of the entire P‐TEFb complex. To our knowledge, this unique property of co‐degrading the Cyclin T and CDK9 is a specific feature conferred by the RG7388‐based MDM2‐recruitment PROTAC strategy and represents a novel paradigm not observed in other CDK9‐targeting PROTACs.

In addition, our findings reveal that targeted degradation of CDK9 initiates a cascade of secondary transcriptional repression. The on‐target CDK9 depletion impairs global transcriptional homeostasis, leading to the downregulation of mRNAs for multiple CDKs (e.g., *CDK2*, *CDK4*, *CDK6*, and *CDK12*) and a consequent suppression of their protein abundance. This hierarchical regulatory network, where CDK9 acts as a central transcriptional node upstream of other CDKs, uncovers an inherent oncogenic vulnerability. Therefore, targeting CDK9 and Cyclin T also exerts collateral anti‐proliferative effects through the indirect suppression of a broader CDK network in cancer cells.

The biological functions of Cyclin T1 and T2 have been primarily attributed to their role in forming the P‐TEFb complex. However, it is possible that Cyclin T proteins possess CDK9‐independent functions. In this context, conventional ATP‐competitive inhibitors or CDK9‐only degraders leave the potential scaffolding and protein‐interaction functions of Cyclin T intact. Therefore, the co‐degradation of Cyclin T with CDK9 may offer a distinct therapeutic advantage. Future studies directly comparing the transcriptional and phenotypic consequences of CDK9 inhibition, CDK9 degradation, and P‐TEFb degradation will be valuable to fully delineate this advantage.

Another significant advantage of dCDK9‐010 lies in its selectivity profile. Unlike CRBN‐based degraders, which often exhibit off‐target degradation of neo‐substrates like GSPT1 or IKZF1/3, dCDK9‐010 leverages MDM2 as a tumor‐enriched E3 ligase. This strategy minimizes on‐target toxicity in non‐malignant tissues, as evidenced by the compound's minimal effects on ASC52telo mesenchymal stem cells and p53‐compromised HEK293T cells. The p53‐dependent selectivity of dCDK9‐010 is particularly advantageous in the clinic, as *TP53* wild‐type status is a common cancer biomarker. Furthermore, the translational potential of dCDK9‐010 is supported by its favorable pharmacokinetic properties including moderate half‐life, low clearance, and good bioavailability. The significant tumor growth inhibition in both NSCLC and Ewing sarcoma xenografts, without observable toxicity, highlights the desirable therapeutic index of this agent.

Of note, our work demonstrates the feasibility of MDM2‐mediated degradation of CDK9/Cyclin T complex, in contrast to prior unsatisfactory attempts to recruit MDM2 for the degradation of other CDKs. These results raise a key outstanding question: What are the structural principles governing MDM2's adoption of neo‐substrates, especially in such “bridged PROTAC” mechanisms? Nevertheless, whether the activity of MDM2 to degrade protein complex can be generalized to other targets needs to be explored. Future studies integrating structural biology or artificial intelligence‐driven modeling of ternary complexes will be crucial to elucidate this mode of action and to rationally design next‐generation MDM2‐recruiting protein degraders with enhanced specificity and efficacy.

While this study establishes strong preclinical proof‐of‐concept, its translational potential remains to be fully realized. To bridge this gap, future efforts will be required to identify predictive biomarkers and rigorously evaluate the therapeutic window of these MDM2‐recruiting P‐TEFb degraders in selective cancer models, and ultimately, in clinical settings.

In summary, this work exploits MDM2's E3 ligase activity to create bifunctional molecules that simultaneously degrade CDK9/Cyclin T complex and stabilize p53. To our knowledge, compound **13** (dCDK9‐010) represents the first‐in‐class MDM2‐recruiting PROTAC achieving dual degradation of CDK9/Cyclin T complex, with compelling in vivo antitumor efficacy and safety profile. Given the significant number of human cancers with *TP53* wild‐type status and MDM2 overexpression, MDM2‐recruiting CDK9/Cyclin T PROTACs represent promising targeted therapeutic agents for *MDM2*&*TP53*‐proficient malignancies.

## 4 Materials and Methods

### 4.1 Chemistry

*General experiment and information*: Unless otherwise noted, all purchased reagents were used as received without further purification. ^1^H nuclear magnetic resonance (NMR) and ^13^C NMR spectra were recorded on a Bruker Advance 500 or 600 MHz spectrometer. ^1^H NMR spectra are reported in parts per million (ppm) downfield from tetramethylsilane (TMS). All ^13^C NMR spectral peaks are reported in ppm and measured with ^1^H decoupling. In reported spectral data, the format (*δ*) chemical shift (multiplicity, *J* values in Hz, integration) was used with the following abbreviations: s = singlet, d = doublet, t = triplet, q = quartet, m = multiplet. Mass spectrometric (MS) analysis was carried out with a Waters ultra‐performance liquid chromatography (UPLC) mass spectrometer. The final compounds were all purified by C18 reverse phase preparative high‐performance liquid chromatography (HPLC) column with solvent A (0.1% formic acid in H_2_O) and solvent B (0.1% formic acid in methanol) as eluents. The purity of all the final compounds was shown to be > 95% by UPLC−MS or UPLC. The detailed synthesis is available in the Supporting Information.

### 4.2 Cell Lines and Cell Culture

The cell lines utilized in this study were cultured under standard conditions. ASC52telo (ATCC, USA) was maintained in mesenchymal stem cell basal medium supplemented with a mesenchymal stem cell growth Kit (ATCC). HEK293T (ATCC), TC‐32 (COG Repository, USA), HCT116 (ATCC), A549 (ATCC), HEPG2 (ATCC), HT1080 (ATCC), MDA‐MB‐231 (ATCC), U87 (U‐87MG, ATCC), U251 (U251MG, ATCC), and MKN45 (ATCC) cells lines were maintained in Dulbecco's modified Eagle medium (DMEM; Sigma‐Aldrich, Taufkirchen, Germany). NCI‐H460 (ATCC), NCI‐H226 (ATCC), NCI‐H1299 (ATCC), KYSE‐150 (DSMZ, Germany), DLD‐1 (ATCC), THP‐1 (ATCC), NALM6 (ATCC), and REH (ATCC) cells were maintained in Roswell Park Memorial Institute medium 1640 (RPMI‐1640; Meilunbio, Dalian, China). With the exception of ASC52telo, all cell lines were cultured in their respective media supplemented with 10% fetal bovine serum (FBS, Vazyme, Nanjing, China) and 1% penicillin‐streptomycin (Beyotime, Shanghai, China), and were incubated at 37°C in a humidified atmosphere containing 5% CO_2_. All cell lines were authenticated by short tandem repeat (STR) profiling and confirmed to be free of mycoplasma contamination by MycoBlue Mycoplasma Detector (Vazyme). STR profiles matched reference databases to ensure cell line identity. RNAiMAX (Invitrogen, Waltham, MA, USA) was used for siRNA transfection. ON‐TARGETplus Non‐targeting Pool (D‐001810‐10‐05), ON‐TARGETplus SMART‐pool siRNAs against *MDM2* (L‐003279‐00‐0005), *MDM4* (L‐006536‐02‐0005), and *TP53* (L‐003329‐00‐0005) were purchased from Dharmacon (Lafayette, CO, USA). Short hairpin RNA (shRNA) vector targeting CDK9 (target sequence: AGGGACATGAAGGCTGCTAAT) was constructed based on pLKO.1 backbone. SHC002 (Sigma‐Aldrich) was used as non‐target control (sh‐NT). All‐in‐one lentiCRISPRv2‐based sgRNA vectors were constructed to edit *TP53* (target sequences: GAGCGCTGCTCAGATAGCGA, ATGTGTAACAGTTCCTGCAT). Stable cell lines were generated by lentiviral transduction followed by Puromycin selection (Beyotime).

### 4.3 Western Blotting

Cells were harvested on ice by scraping, pelleted by centrifugation, and lysed to prepare whole‐cell extracts. Lysis was performed by resuspending the cell pellets in Lysis Buffer (420 mM NaCl, 5% glycerol, 0.1% NP‐40, 0.1 mM EDTA, 50 mM Tris pH 8.0) with fresh addition of 1 mM dithiothreitol (Beyotime), 1 mM phenylmethylsulfonyl fluoride (Sigma‐Aldrich), 1x protease inhibitor cocktail (TargetMol, Wellesley Hills, MA, USA), 1x phosphatase inhibitor cocktail (Targetmol), and 1 mM MgCl_2_ and BenzoNuclease (Novoprotein, Suzhou, China) for 20 min on ice with occasional vortexing. Following centrifugation, the supernatant was collected for protein concentration determination using the Bradford assay (Beyotime), and subsequent sample were prepared for analysis by sodium dodecyl sulfate polyacrylamide gel electrophoresis. Standard protocols were followed for immunoblot analysis. The following primary antibodies were used: CDK9 (Cell Signaling Technology, 2316), CDK9 (Santa Cruz, sc‐13130), Cyclin T1 (Cell Signaling Technology, 81464), Cyclin T2 (Santa Cruz, sc‐81243), p53 (Santa Cruz, sc‐126), p21 (Cell Signaling Technology, 2947), ACTIN (Proteintech, 66009‐1‐Ig), AFF1 (Upingbio, YP‐mAb‐16926), AFF4 (Proteintech, 14662‐1‐AP), BRD4 (Cell Signaling Technology, 13440), Cyclin K (Sangon, D124444), CDK1 (Diagbio, db12527), CDK2 (Uptbio, PLA015198), CDK3 (Uptbio, PLA006035), CDK4 (Cell Signaling Technology, 12790), CDK5 (Santa Cruz, sc‐173), CDK6 (Cell Signaling Technology, 13331), CDK7 (Bethyl Laboratories, A300‐405A), CDK8 (Santa Cruz, sc‐13155), CDK11 (Abclonal, A12830), CDK12 (Cusabio, CSB‐PA882147LA01HU), CDK13 (Proteintech, 30461‐1‐AP), Cleaved Caspase‐3 (Diagbio, db15982), Cleaved PARP (Cell Signaling Technology, 5625), FLI1 (Abcam, ab15289), MDM2 (Genetex, GTX100531), RNA polymerase II Rpb1 (Diagbio, db15872), and phospho‐Rpb1 CTD^Ser2^ (Cell Signaling Technology, 13499). Horseradish peroxidase‐conjugated goat anti‐mouse IgG and goat anti‐rabbit IgG were used as secondary antibodies for detection. Quantification of western blot bands was performed using ClinxChemi software. After background subtraction, the grayscale values of target proteins were normalized to the ACTIN loading control in the same lane.

### 4.4 Cell Viability Assay and Chemical Treatment

Cell viability was assessed using the MTT (thiazolyl blue) colorimetric assay. Cells were seeded in 96‐well plates at densities ranging from 1200 to 15,000 cells per well, optimized according to their proliferation rates. After a 72‐h incubation with the compounds, the dose–response relationships were determined by adding MTT reagent (Coolaber) and incubating for 3 h at 37°C. For adherent cells, the medium was aspirated before adding 100 µL of MTT STOP solution; for suspension cells (leukemic lines), the assay was performed without the aspiration step. Absorbance was measured at 570 nm using a microplate reader. Apart from those synthesized in house, commercially obtained chemicals used in culture assays included DMSO (Solarbio, Beijing, China; D8371), SNS032 (Targetmol, T6049), THAL‐SNS032 (Targetmol, T17069), RG7388 (Targetmol, T6254), and (R)‐MG132 (Targetmol, T12628).

### 4.5 BrdU Incorporation Assay

Cell proliferation was assessed using the BrdU Cell Proliferation Assay Kit (BioVision, Denver, CO, USA; K306). Cells were seeded in 96‐well plates at 5000 cells per well and allowed to adhere overnight. After 24‐h treatment with indicated compounds, BrdU solution (10 µM final concentration) was added to each well and incubated for 3 h at 37°C. Cells were then fixed, and BrdU incorporation was quantified via anti‐BrdU antibody‐based enzyme‐linked immunosorbent assay according to manufacturer's protocol.

### 4.6 Caspase‐Glo 3/7 Assay

Apoptosis was monitored by measuring Caspase‐3/7 activity using the Caspase‐Glo 3/7 Assay (Promega, Madison, WI, USA; G8091). Cells were seeded in white‐walled 96‐well plates at 10,000 cells per well. After 24‐h compound treatment, 100 µL of Caspase‐Glo reagent was added to each well. Luminescence was measured using a FlexStation 3 microplate reader (Molecular Devices, San Jose, CA, USA). Signals from cell‐free medium were subtracted as background.

### 4.7 RNA Preparation and RNA‐seq Analysis

Total RNA was extracted from cell samples using the FastPure Cell/tissue total RNA isolation kit (Vazyme) according to the manufacturer's instructions. Total RNA was DNase‐treated to eliminate genomic DNA contamination. First‐strand complementary DNA (cDNA) was synthesized using the HiScript III first Strand cDNA Synthesis Kit (Vazyme, R312‐02). qPCR was performed using ChamQ Universal SYBR qPCR Master Mix (Vazyme, Q711‐02) on a CFX Opus 96 Real‐Time PCR System (Bio‐Rad, Hercules, CA, USA). All reactions were run in technical triplicates, and melting curve analysis was performed to confirm amplicon specificity. All primer sequences are provided in Table S2. For RNA‐seq, stranded mRNA libraries were constructed and paired‐end sequenced (150 bp) on an Illumina NovaSeq 6000 by Seqhealth Technology Co. (Wuhan, China). Raw sequencing data quality was assessed using FastQC (v0.11.9) and MultiQC (v1.13). Reads were then aligned to the Ensembl GRCh38 reference genome with STAR (v2.7.11b). Alignment metrics were generated with RSeQC (v5.0.3). Gene‐level quantification was performed using RSEM (v1.3.3). Differential expression analysis was conducted with DESeq2 (v1.44.0). GSEA used clusterProfiler (v4.0.5) with visualization via enrichplot (v1.16.0) and ggplot2 (v3.4.0). Raw sequencing data are deposited in the Genome Sequence Archive (GSA: HRA012261) at China National Center for Bioinformation (https://ngdc.cncb.ac.cn/gsa).

### 4.8 Antitumor Efficacy Studies in Mice

All animal experiments were approved by the Zhejiang University Institutional Animal Care and Use Committee (Protocol ID: ZJU20210146, ZJU20241058) and were conducted in accordance with relevant institutional and national guidelines. NCG (NOD/ShiLtJGpt‐*Prkdc^em26Cd52^Il2rg^em26Cd22^*/Gpt) mice (female, 6‐ to 8‐week‐old) were used to establish subcutaneous xenograft models of NCI‐H226 and TC‐32 cells. One million of cancer cells were resuspended in 100 µL of PBS/Matrigel (LABLEAD, Beijing, China; MG4248) solution (1:1) and implanted subcutaneously into dorsal flank of recipient mice. To access the antitumor efficacy of dCDK9‐010 in vivo, mice bearing established tumors were randomly assigned to treatment groups 5 days post‐implantation. dCDK9‐010 was administered intravenously at a dose of 20 mg/kg every other day. The vehicle control group received an equivalent volume of 10% Kolliphor HS 15 in saline. Tumor size was measured by caliper and tumor volume (mm^3^) was estimated according to the following formula: 1/2 × (Length × Width^2^). Mice were euthanized at the experimental endpoint, and tumors were excised and weighed. No specific randomization algorithm was used to assign mice into different treatment groups. The investigators were not blinded to group allocation during the experiment or outcome assessment. No animals or data points were excluded from these experiments.

### 4.9 PK Studies in Mice

All pharmacokinetic studies of dCDK9‐010 were performed following the current Technical Guidelines for Non‐clinical Pharmacokinetic Studies of Drugs (National Medical Products Administration) and the current guidelines of the International Conference on Harmonization. Six‐ to eight‐week‐old male CD‐1 (ICR; Charles River, Beijing, China) mice were used for the pharmacokinetic studies. Six mice were randomly assigned to two groups (three mice per group). In the intravenous injection and intraperitoneal injection groups, about 50 µL of blood was collected from the cheek vein at specified time points (5 min, 15 min, 30 min, 1 h, 2 h, 4 h, 6 h, 8 h, and 24 h) after dCDK9‐010 administration. The concentration of dCDK9‐010 in mouse plasma samples was quantified by liquid chromatography mass spectrometry (LC‐MS)/MS method. Pharmacokinetic parameters were calculated from the plasma concentration–time data using WinNolin software.

## Author Contributions

Supervision: Xin Han, Liang Xu, and Ye Chen. Conceptualization and Methodology: Xin Han, Liang Xu, Ye Chen, Xian Guan, and Long Xie. Validation and Investigation: Xian Guan, Long Xie, Hanjun Guo, Jiawei Zhou, Lisong Luo, Hao Yang, Yuanfang Wu, Jiangyu Liu, Yue Wang, Xingze Huang, Jiyang Liu, Ying Zhang, and Wenhao Chen. Compounds design and synthesis: Xian Guan and Lin Ma. Bioactivity testing and animal experiments: Long Xie, Jiawei Zhou, Jiyang Liu, Ying Zhang, Wenhao Chen, and Ye Chen. Writing – review and editing: Xian Guan, Long Xie, Ye Chen, Liang Xu, and Xin Han. All authors have read and approved the final manuscript.

## Funding

This work is supported by the National Natural Science Foundation of China (82272637, 82204429, 82203247, 82203415, 82372668, 32270746), Zhejiang Provincial Natural Science Foundation of China (LZ24H160004), Fundamental Research Funds for the Central Universities (226‐2025‐00101), and Medical Interdisciplinary Innovation Program 2024, Zhejiang University School of Medicine.

## Ethics Statement

All animal experiments were conducted in accordance with ethical approval from Zhejiang University Institutional Animal Care and Use Committee (Protocol ID: ZJU20210146, ZJU20241058).

## Conflicts of Interest

The authors declare no conflicts of interest.

## Supporting information




**Figure S1. CDK9 degradation efficiency of newly designed compounds in NCI‐H226 cells**. Cells were treated with indicated treatment for 8 hours and subjected to immunoblotting assay. CDK9 short isoform (CDK9‐S, 42 kD) was detected as the predominant form of CDK9. Results are representative of three independent experiments.
**Figure S2. CDK9 degradation efficiency of newly designed compounds in TC‐32 cells**. Cells were treated with indicated treatment for 8 hours and subjected to immunoblotting assay. CDK9 short isoform (CDK9‐S, 42 kD) was detected as the predominant form of CDK9. Results are representative of three independent experiments.
**Figure S3. Dose‐response curves of NCI‐H226 cell viability following treatment with newly designed compounds**. Cells were cultured under indicated treatment for 72 hours and subjected to MTT assay. Data represent three independent biological experiments, each with ≥2 technical replicates.
**Figure S4. Dose‐response curves of TC‐32 cell viability following treatment with newly designed compounds**. Cells were cultured under indicated treatment for 72 hours and subjected to MTT assay. Data represent three independent biological experiments, each with ≥2 technical replicates.
**Figure S5**. **Transcriptional response of CDK genes to MDM2‐receuiting P‐TEFb PROTAC degraders**. (A) Temporal changes of *CDK2* mRNA levels in response to **12** (dCDK9‐009) and **13** (dCDK9‐010) treatments (2 µM) in NCI‐H226 cells. (B‐F) Temporal changes of *CDK4*, *CDK6*, *CDK11*, *CDK12*, and *CDK13* mRNA levels in response to **12** (dCDK9‐009) and **13** (dCDK9‐010) treatments (2 µM) in NCI‐H226 cells. *CDK11* qPCR primer amplified both *CDK11A* and *CDK11B*. qPCR data were presented as mean ± SD; ***, p < 0.001 based on one‐way ANOVA.
**Figure S6**. **Efficacy of MDM2‐ and CRBN‐recruiting CDK9 PROTAC degraders in degradation of CDK9 and Cyclin T**. NCI‐H226 and TC‐32 cells were treated with compound **12** (2 µM), compound **13** (2 µM), THAL‐SNS032 (2 µM), and dCDK9‐202 (500 nM) for 8 hours. THAL‐SNS032 and dCDK9‐202 are CRBN‐recruiting CDK9 PROTAC degraders made of the same CDK9‐binding warhead (SNS032)
**Figure S7**. **B** **inding** **a** **ffinitie** **s** **of newly designed compounds toward CDK9 protein** **measured by Bio‐Layer Interferometry**. **Figure S8**. **Dose‐response** **curves of** ***TP53*** **‐defective cancer cells to compound** **12** (dCDK9‐009) **and compound** **13** (dCDK9‐010). *TP53*‐mutated: DLD‐1 (colon cancer, S241F), MDA‐MB‐231 (breast cancer, R280K), KYSE‐150 (esophageal squamous cell carcinoma, R248Q); *TP53*‐null: THP‐1 (acute monocytic leukemia)
**Figure S9**. Volcano plot visualizing the differentially enriched pathways in TC‐32 cells treated with dCDK9‐010 (500 nM, 8 h). GSEA of RNA‐seq data was performed through interrogating C2 curated gene sets within MSigDB database in TC‐32 cells.
**Table S1**. Comprehensive Summary of Chemical Information for All Final Products. **Table S2**. Sequences of qPCR primers

## Data Availability

Raw RNA‐seq data generated from in this study have been deposited in the Genome Sequence Archive in National Genomics Data Center, China National Center for Bioinformation (https://ngdc.cncb.ac.cn/gsa), and are available under accession HRA012261. The following Supporting Information is available free of charge: ^1^H and ^13^C NMR Spectra for representative CDK9 degraders. HPLC purity spectra for CDK9 degraders. LC‐MS/HRMS spectra for CDK9 degraders. The data that support the findings of this study are available from the corresponding author upon reasonable request.
