# Supplementary material for: Discovery of a First‐in‐Class Murine Double Minute 2‐Recruiting Positive Transcription Elongation Factor B PROTAC Degrader With Selective Antitumor Activity
Source: MedComm (2020). 2026 Apr 9;7(4):e70723. doi: 10.1002/mco2.70723 (PMC13066720; doi:10.1002/mco2.70723)
Supplement: Supplementary file 1 — Figure S1. CDK9 degradation efficiency of newly designed compounds in NCI‐H226 cells. Cells were treated with indicated treatment for 8 hours and subjected to immunoblotting assay. CDK9 short isoform (CDK9‐S, 42 kD) was detected as the predominant form of CDK9. Results are representative of three independent experiments. Figure S2. CDK9 degradation efficiency of newly designed compounds in TC‐32 cells. Cells were treated with indicated treatment for 8 hours and subjected to immunoblotting assay. CDK9 short isoform (CDK9‐S, 42 kD) was detected as the predominant form of CDK9. Results are representative of three independent experiments. Figure S3. Dose‐response curves of NCI‐H226 cell viability following treatment with newly designed compounds. Cells were cultured under indicated treatment for 72 hours and subjected to MTT assay. Data represent three independent biological experiments, each with ≥2 technical replicates. Figure S4. Dose‐response curves of TC‐32 cell viability following treatment with newly designed compounds. Cells were cultured under indicated treatment for 72 hours and subjected to MTT assay. Data represent three independent biological experiments, each with ≥2 technical replicates. Figure S5. Transcriptional response of CDK genes to MDM2‐receuiting P‐TEFb PROTAC degraders. (A) Temporal changes of CDK2 mRNA levels in response to 12 (dCDK9‐009) and 13 (dCDK9‐010) treatments (2 µM) in NCI‐H226 cells. (B‐F) Temporal changes of CDK4, CDK6, CDK11, CDK12, and CDK13 mRNA levels in response to 12 (dCDK9‐009) and 13 (dCDK9‐010) treatments (2 µM) in NCI‐H226 cells. CDK11 qPCR primer amplified both CDK11A and CDK11B. qPCR data were presented as mean ± SD; ***, p < 0.001 based on one‐way ANOVA. Figure S6. Efficacy of MDM2‐ and CRBN‐recruiting CDK9 PROTAC degraders in degradation of CDK9 and Cyclin T. NCI‐H226 and TC‐32 cells were treated with compound 12 (2 µM), compound 13 (2 µM), THAL‐SNS032 (2 µM), and dCDK9‐202 (500 nM) for 8 hours. THAL‐SNS032 and dCDK9‐202 [file MCO2-7-e70723-s001.docx]

**Supporting Information**

**Discovery of a First-in-Class Murine Double Minute 2-Recruiting Positive Transcription Elongation Factor B PROTAC Degrader With Selective Antitumor Activity**

Xian Guan^1,#^, Long Xie^2,^^#^, Hanjun Guo^2,3,4^, Lin Ma^1^, Jiawei Zhou^3,4^, Lisong Luo^2^, Hao Yang^1^, Yuanfang Wu^3,4^, Jiangyu Liu^3^, Yue Wang^2^, Xingze Huang^2^, Jiyang Liu^2^, Ying Zhang^2^, Wenhao Chen^4,5^, Ye Chen^3,4,6,^*, Liang Xu^2,6,7^* and Xin Han^1,6,^*

^1^ Cancer Institute (Key Laboratory of Cancer Prevention and Intervention, China National Ministry of Education) of the Second Affiliated Hospital and Institute of Translational Medicine, Zhejiang University School of Medicine, Hangzhou 310029, China.

^2^ Institute of Biochemistry, College of Life Sciences, Zhejiang University, Hangzhou 310058, China.

^3^ Department of Surgical Oncology, Children's Hospital Zhejiang University School of Medicine, National Clinical Research Center for Children and Adolescents' Health and Diseases, Hangzhou 310052, China.

^4^ Pediatric Cancer Research Center, National Clinical Research Center for Children and Adolescents' Health and Diseases, Hangzhou 310052, China.

^5^ Department of Orthopedic Surgery, Children's Hospital Zhejiang University School of Medicine, National Clinical Research Center for Children and Adolescents' Health and Diseases, Hangzhou 310052, China.

^6^ Cancer Center of Zhejiang University, Hangzhou 310029, China.

^7^ Department of Radiation Oncology, Key Laboratory of Cancer Prevention and Intervention, The Second Affiliated Hospital, Zhejiang University School of Medicine, Hangzhou 310009, China.

^#^These authors contribute equally.

*Corresponding authors.

E-mail addresses: [chenyephd@zju.edu.cn](mailto:chenyephd@zju.edu.cn) (Y. Chen), [xuliang.phd@zju.edu.cn](mailto:xuliang.phd@zju.edu.cn) (L. Xu) and [xinhan@zju.edu.cn](mailto:xinhan@zju.edu.cn) (X. Han)

**Contents:**

**Part 1. Supplementary Tables and Figures…...………………………………………………S3**

**Part 2. Synthesis and characterization…..……………………………………………….…S14**

**Part 3. ^1^H and ^13^C NMR Spectra.………..………………………………………………….S40**

**Part 4. UPLC-MS results...…………………….……………………………………...……...S60**

**Part 5. HRMS spectra……………………………………………………...………………..S80**

**Part 1. Supplementary Tables and Figures**

**Figure S1. CDK9 degradation efficiency of newly designed compounds in NCI-H226 cells.** Cells were treated with indicated treatment for 8 hours and subjected to immunoblotting assay. CDK9 short isoform (CDK9-S, 42 kD) was detected as the predominant form of CDK9. Results are representative of three independent experiments.

**Figure S2. CDK9 degradation efficiency of newly designed compounds in TC-32 cells.** Cells were treated with indicated treatment for 8 hours and subjected to immunoblotting assay. CDK9 short isoform (CDK9-S, 42 kD) was detected as the predominant form of CDK9. Results are representative of three independent experiments.


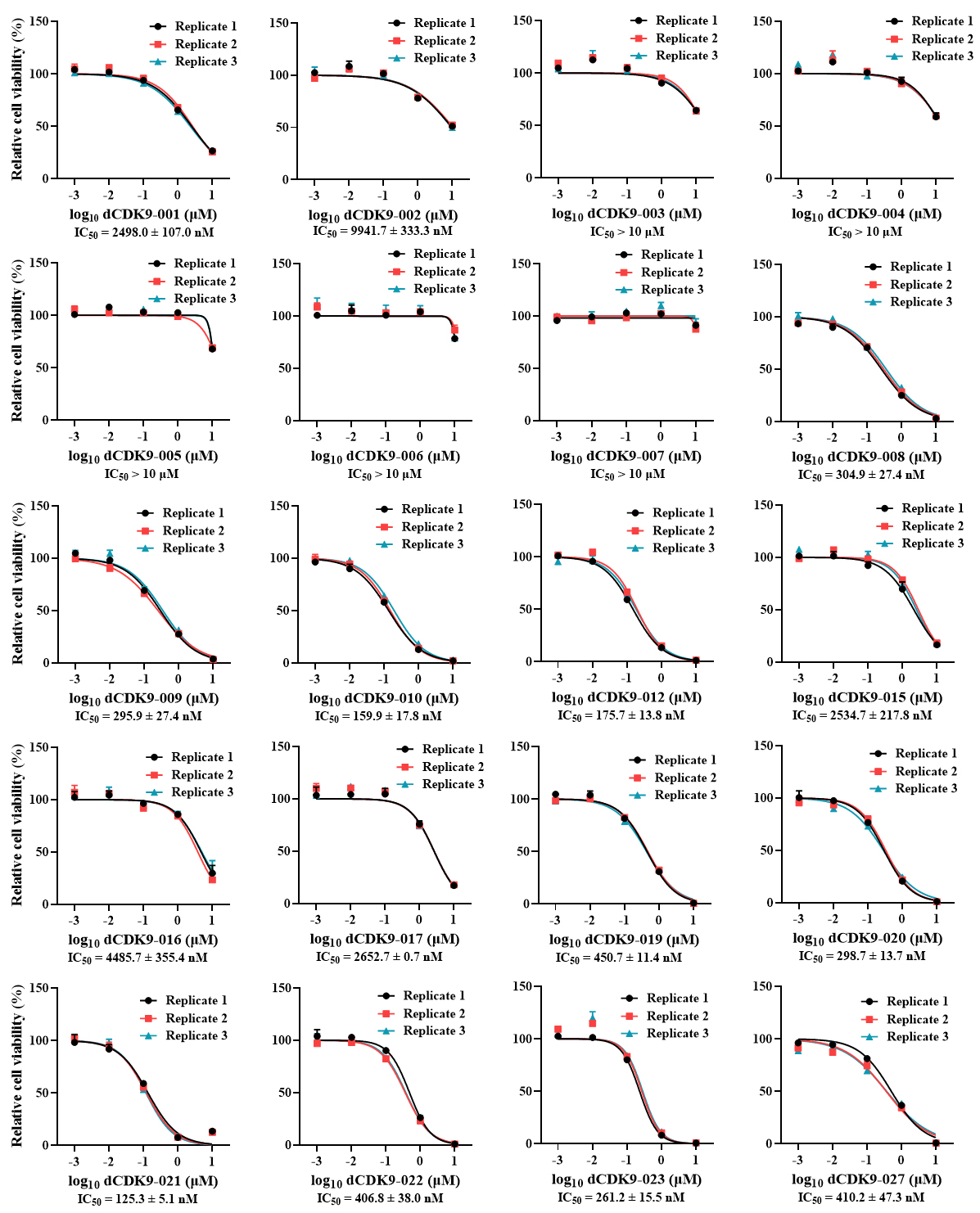


**Figure S3. Dose-response curves of NCI-H226 cell viability following treatment with newly designed compounds.** Cells were cultured under indicated treatment for 72 hours and subjected to MTT assay. Data represent three independent biological experiments, each with ≥2 technical replicates.


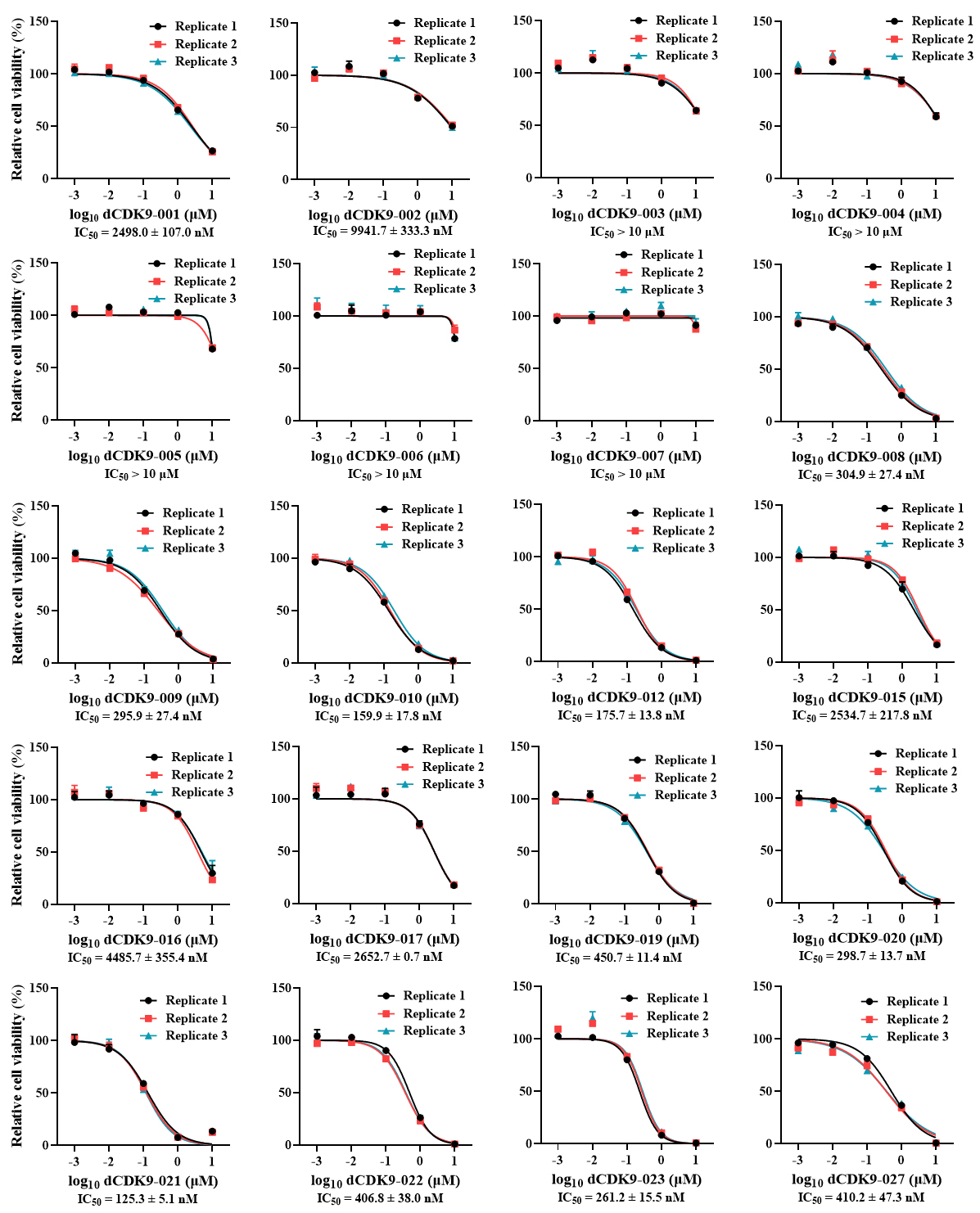


**Figure S4. Dose-response curves of TC-32 cell viability following treatment with newly designed compounds.** Cells were cultured under indicated treatment for 72 hours and subjected to MTT assay. Data represent three independent biological experiments, each with ≥2 technical replicates.

**Figure S5**. Transcriptional response of CDK genes to MDM2-receuiting P-TEFb PROTAC degraders. (A) Temporal changes of *CDK2* mRNA levels in response to **12** (dCDK9-009) and **13** (dCDK9-010) treatments (2 μM) in NCI-H226 cells. (B-F) Temporal changes of *CDK4*, *CDK6*, *CDK11*, *CDK12*, and *CDK13* mRNA levels in response to **12** (dCDK9-009) and **13** (dCDK9-010) treatments (2 μM) in NCI-H226 cells. *CDK11* qPCR primer amplified both *CDK11A* and *CDK11B*. qPCR data were presented as mean ± SD; ***, p < 0.001 based on one-way ANOVA.

**Figure S6**. Efficacy of MDM2- and CRBN-recruiting CDK9 PROTAC degraders in degradation of CDK9 and Cyclin T. NCI-H226 and TC-32 cells were treated with compound **12** (2 μM), compound **13** (2 μM), THAL-SNS032 (2 μM), and dCDK9-202 (500 nM) for 8 hours. THAL-SNS032 and dCDK9-202 are CRBN-recruiting CDK9 PROTAC degraders made of the same CDK9-binding warhead (SNS032).

**Figure S7. Binding affinities of newly designed compounds toward CDK9 protein measured by Bio-Layer Interferometry (BLI).** Recombinant CDK9 protein (Proteintech, Wuhan, China) was biotinylated using NHS-LC-LC-BIOTIN (Pierce, Rockford, IL, USA). Briefly, CDK9 protein (50 μg/mL) was prepared in 50 mM phosphate buffer (pH 6.5), and a 10-fold molar excess of biotin reagent was added to achieve preferential N-terminal biotinylation of CDK9 protein. The mixture was incubated in the dark at room temperature for at least 1 h. Unconjugated biotin was subsequently removed via size-exclusion chromatography using a PD-10 desalting column (GE Healthcare, Vienna, Austria). The biotinylated CDK9 protein was dissolved in phosphate-buffered saline (PBS). Prior to each assay, streptavidin (SA)-coated biosensors were pre-wetted in PBS to establish a stable baseline. Biotinylated CDK9 protein was then immobilized onto the surface of SA-coated biosensors in a 96-well plate. Subsequently, a 200 μL solution containing various concentrations of SNS032, dCDK9-004, dCDK9-007 and dCDK9-010 diluted by PBS (containing 0.02% Tween 20) was added to each well. The binding assays were conducted with repeated cycles comprising four sequential steps: initial loading, baseline stabilization, association, and dissociation. Data acquisition and analysis were performed using a ForteBio Octet R8 system (Sartorius).

**Figure S8**. Dose-response curves of *TP53*-defective cancer cells to compound **12** (dCDK9-009) and compound **13** (dCDK9-010). *TP53*-mutated: DLD-1 (colon cancer, S241F), MDA-MB-231 (breast cancer, R280K), KYSE-150 (esophageal squamous cell carcinoma, R248Q); *TP53*-null: THP-1 (acute monocytic leukemia).

**Figure S9**. Volcano plot visualizing the differentially enriched pathways in TC-32 cells treated with dCDK9-010 (500 nM, 8 h). GSEA of RNA-seq data was performed through interrogating C2 curated gene sets within MSigDB database in TC-32 cells.

**Table S1**. Comprehensive Summary of Chemical Information for All Final Products

| **No.** | **Compound** | **Linker** | **MDM2 E3 Ligands** | **Molecular formula** | **M.W.** |
| --- | --- | --- | --- | --- | --- |
| **4** | dCDK9-001 |  | RG7388 | C_54_H_62_Cl_2_F_2_N_8_O_6_S_2_ | 1092.16 |
| **5** | dCDK9-002 |  | RG7388 | C_55_H_64_Cl_2_F_2_N_8_O_6_S_2_ | 1106.18 |
| **6** | dCDK9-003 |  | RG7388 | C_56_H_66_Cl_2_F_2_N_8_O_6_S_2_ | 1120.21 |
| **7** | dCDK9-004 |  | RG7388 | C_57_H_68_Cl_2_F_2_N_8_O_6_S_2_ | 1134.24 |
| **8** | dCDK9-005 |  | RG7388 | C_58_H_70_Cl_2_F_2_N_8_O_6_S_2_ | 1148.26 |
| **9** | dCDK9-006 |  | RG7388 | C_59_H_72_Cl_2_F_2_N_8_O_6_S | 1162.29 |
| **10** | dCDK9-007 |  | RG7388 | C_60_H_74_Cl_2_F_2_N_8_O_6_S_2_ | 1176.32 |
| **11** | dCDK9-008 |  | RG7388 | C_53_H_60_Cl_2_F_2_N_8_O_7_S_2_ | 1094.13 |
| **12** | dCDK9-009 |  | RG7388 | C_55_H_64_Cl_2_F_2_N_8_O_8_S_2_ | 1138.18 |
| **13** | dCDK9-010 |  | RG7388 | C_57_H_68_Cl_2_F_2_N_8_O_9_S_2_ | 1182.23 |
| **14** | dCDK9-012 |  | RG7388 | C_59_H_72_Cl_2_F_2_N_8_O_10_S_2_ | 1226.29 |
| **15** | dCDK9-015 |  | Nutlin-3 | C_54_H_63_Cl_2_N_9_O_9_S_2_ | 1117.17 |
| **16** | dCDK9-016 |  | Nutlin-3 | C_56_H_67_Cl_2_N_9_O_10_S_2_ | 1161.23 |
| **17** | dCDK9-017 |  | Nutlin-3 | C_58_H_71_Cl_2_N_9_O_11_S_2_ | 1205.28 |
| **18** | dCDK9-019 |  | RG7388 | C_58_H_69_Cl_2_F_2_N_9_O_7_S_2_ | 1177.26 |
| **19** | dCDK9-020 |  | RG7388 | C_60_H_73_Cl_2_F_2_N_9_O_8_S_2_ | 1221.32 |
| **20** | dCDK9-021 |  | RG7388 | C_56_H_66_Cl_2_F_2_N_10_O_6_S_2_ | 1148.22 |
| **21** | dCDK9-022 |  | RG7388 | C_61_H_75_Cl_2_F_2_N_9_O_5_S_2_ | 1187.35 |
| **22** | dCDK9-023 |  | RG7388 | C_57_H_69_Cl_2_F_2_N_9_O_5_S_2_ | 1133.25 |
| **23** | dCDK9-027 |  | RG7388 | C_57_H_69_Cl_2_F_2_N_9_O_6_S_2_ | 1149.25 |

**Table S2**. Sequences of qPCR primers

| **qPCR primer** | **Sequence (5’ → 3’)** |
| --- | --- |
| ACTB-F | GACGACATGGAGAAAATCTG |
| ACTB-R | ATGATCTGGGTCATCTTCTC |
| CDK2-F | GTACCTCCCCTGGATGAAGAT |
| CDK2-R | CGAAATCCGCTTGTTAGGGTC |
| CDK4-F | CTGGTGTTTGAGCATGTAGACC |
| CDK4-R | GATCCTTGATCGTITCGGCTG |
| CDK6-F | CCAGATGGCTCTAACCTCAGT |
| CDK6-R | AACTTCCACGAAAAAGAGGCTT |
| CDK11-F | TCAAGCAGGAGCTGCCCAAG |
| CDK11-R | ATGCTGGGCCTTGAGGATGG |
| CDK12-F | ACCGAAGGGAGAACGACGAA |
| CDK12-R | GAACGCTTTGAACTTCCCGAT |
| CDK13-F | CTTCAGGTAACGAAGGTGGAAAA |
| CDK13-R | CCTTGGCTCCTATGCTTGGTG |

**Part 2. Synthesis and characterization**

Compounds **4**-**10** were prepared in Scheme S1, compounds **11**-**14** were prepared in Scheme S2, compounds **15**-**17** were prepared in Scheme S3, compounds **18**-**19** were prepared in Scheme S4, compound **20** was prepared in Scheme S5, compound **21** was prepared in Scheme S6, compound **22** was prepared in Scheme S7 and compound **23** was prepared in Scheme S8.

**Scheme S1**. Synthesis of **4**-**10**. (a) TEA, DCM, 0 ^o^C; (b) POCl3, reflux; (c) MeOH, rt; (d) 4, NaBH4, EtOH, Acetone, rt ~ reflux; (e) HATU, DIPEA, DMF, rt; (f) 4 M HCl in 1,4-Dioxane, DCM, rt; (g) HATU, DIPEA, DMF, rt; (h) TFA, DCM, rt; (i) HATU, DIPEA, DMF, rt.

**Scheme S2**. Synthesis of **11**-**14**. (a) HATU, DIPEA, DMF, rt; (b) TFA, DCM, rt.; (c) HATU, DIPEA, DMF, rt.

**Scheme S3**. Synthesis of **15**-**17**. (a) NaH, DMF, 0 ℃ ~ rt; (b) TFA, DCM, rt; (c) HATU, DIPEA, DMF, rt.

**Scheme S4**. Synthesis of **18**-**19**. (a) HATU, DIPEA, DMF, rt; (b) TFA, DCM, rt; (c) K_2_CO_3_, ACN, reflux; (d) TFA, DCM, rt; (e) HATU, DIPEA, DMF, rt.

**Scheme S5**. Synthesis of **20**. (a) K_2_CO_3_, DMF, rt; (b) TFA, DCM, rt; (c) HATU, DIPEA, DMF, rt; (d) TFA, DCM, rt; (e) HATU, DIPEA, DMF, rt.

**Scheme S6**. Synthesis of **21**. (a) K_2_CO_3,_ ACN, reflux; (b) TFA, DCM, rt; (c) K_2_CO_3,_ ACN, reflux; (d) TFA, DCM, rt; (e) HATU, DIPEA, DMF, rt.

**Scheme S7**. Synthesis of **22**. (a) K_2_CO_3,_ ACN, reflux; (b) TFA, DCM, rt; (c) K_2_CO_3,_ ACN, reflux; (d) TFA, DCM, rt; (e) HATU, DIPEA, DMF, rt.

**Scheme S8**. Synthesis of **23**. (a) NaBH_3_CN, DCM/MeOH, rt; (b) TFA, DCM, rt; (c) K_2_CO_3_, ACN, reflux; (d) TFA, DCM, rt; (e) HATU, DIPEA, DMF, rt.

***General Procedure for Synthesis of Compound*** ***4-10****.*

Triethylamine (3.5 eq.) was added to a solution of compound **24** (1.51 g, 10 mmol) in 35 mL DCM at -5 °C. After 30 min, compound **25** (1.1 eq.) was added dropwise at -5 °C slowly. After 1 h at rt, the reaction mixture was quenched with 1 N HCl and extracted with DCM three times. The organic layer was again washed with brine before being dried over MgSO_4_ and the solvent was evaporated under reduced pressure. Compound **26** was obtained by flash column chromatography (PE : EtOAc = 20:1) with 95% yield.

Compound **26** (958 mg, 5 mmol) was placed in a 10 mL round bottomed flask with 3 mL POCl_3_. The reaction mixture was heated to 105 °C and stirred for 1 h. After being cooled to room temperature, the reaction mixture was quenched with ice/H_2_O and extracted with DCM three times. The organic layer was combined and neutralized to pH 7-8 with saturated sodium bicarbonate. Then, the organic layer was again washed with brine before being dried over MgSO_4_ and the solvent was evaporated under reduced pressure. The compound **27** was obtained by flash column chromatography (PE : EtOAc = 4:1) with 76% yield.

A mixture of compound **28** (1.30 g, 5 mmol) and potassium thiocyanate **29** (4 eq.) in 34 mL MeOH was stirred at rt for 48 h. The solvents were evaporated under reduced pressure to aﬀord the corresponding crude. And the crude was diluted by 3 mL H_2_O and the pH of the solution was adjusted to pH = 12 with 10% NaOH. The resulting residue was filtered and the compound **30** was obtained by flash column chromatography (DCM : MeOH = 4:1) with 32% yield.

NaBH_4_ (2 eq.) was slowly added to a solution of compound **30** (314 mg, 2 mmol) in 20 mL absolute EtOH at rt. After 1 h, 10 mL acetone was slowly introduced. After another 1 h at rt, compound **27** (1 eq.) in 3 mL EtOH was added and then the resulting reaction mixture was refluxed for 1 h. After the reaction mixture was cooled to rt, the solvents were evaporated under reduced pressure. Then, H_2_O was added into the resulting residue, which was extracted with EtOAc three times. The organic layer was again washed with brine before being dried over MgSO_4_ and the solvent was removed under vacuum leaving the crude. The compound **31** was puriﬁed by flash column chromatography (PE : EA = 4:1) with 60% yield. ^1^H NMR (500 MHz, DMSO-*d*_6_) *δ* 7.27 (s, 2H), 6.84 (s, 1H), 6.72 (s, 1H), 3.93 (s, 2H), 1.23 (s, 9H). HRMS (ESI) calculated for C_11_H_16_N_3_OS_2_ [M + H]^+^: 270.0729, found: 270.0703.

DIPEA (5 eq.) and HATU (1.5 eq.) were added to a solution of **31** (539 mg, 2 mmol) and compound **32** (1 eq.) in 10 mL DMF. The resulting mixture was stirred at rt overnight. The mixture was quenched with H_2_O and extracted with EtOAc three times. The organic layer was again washed with brine before being dried over MgSO_4_ and the solvent was removed under vacuum leaving the crude. Then the crude was purified by flash column chromatography (PE/EA = 2:1) to afford the intermediate. Then 5 mL 4 M HCl in 1,4-dioxane was added to a solution of the intermediate in 10 mL DCM. The reaction mixture was stirred at rt for 4 h. The solvents were evaporated under reduced pressure to give the compound **1** (SNS032) without further purification (59% yield).

DIPEA (5 eq.) and HATU (1.2 eq.) were added to a solution of compound **1** (381 mg, 1 mmol) and a series of linear acids, referred to as compound **33-39** (1.1 eq.) in DMF (2 mL). After 30 min at rt, the mixture was subject to prep-HPLC to aﬀord the intermediates in 80−90% yields. A solution of the intermediate in 1:1 TFA/DCM was stirred at rt for 30 min. The solvents were evaporated under reduced pressure to give the corresponding deprotected intermediates **40-46** (TFA salt) that were used in the following reactions without further puriﬁcation (45% - 65% yields).

DIPEA (5 eq.) and HATU (1.2 eq.) were added to a solution of compound **40** (49.2 mg, 0.1 mmol) and compound **2** (RG7388) (1.1 eq.) in DMF (2 mL). After 30 min at rt, the mixture was subject to prep-HPLC to aﬀord compound **4**. Following the procedures used to prepare compound **4**, compounds **5**−**10** with diﬀerent chain lengths were obtained by the same methods.

***N*-(5-(((5-(*tert*-butyl)oxazol-2-yl)methyl)thio)thiazol-2-yl)-1-(6-(4-((2*R*,3*S*,4*R*,5*S*)-3-(3-chloro-2-fluorophenyl)-4-(4-chloro-2-fluorophenyl)-4-cyano-5-neopentylpyrrolidine-2-carboxamido)-3-methoxybenzamido)hexanoyl)piperidine-4-carboxamide (4).** White solid; Yield: 65%. ^1^H NMR (600 MHz, DMSO-*d*_6_) *δ* 12.31 (s, 1H), 10.41 (s, 1H), 8.41 (t, *J* = 5.7 Hz, 1H), 8.32 (d, *J* = 8.4 Hz, 1H), 7.74 (t, *J* = 7.2 Hz, 1H), 7.62 – 7.46 (m, 4H), 7.43 – 7.30 (m, 4H), 6.71 (s, 1H), 4.59 (d, *J* = 7.4 Hz, 2H), 4.39 (dd, *J* = 12.7, 6.9 Hz, 2H), 4.05 (s, 2H), 3.93 (s, 5H), 3.26 (q, *J* = 6.7 Hz, 2H), 3.03 (t, *J* = 11.6 Hz, 1H), 2.77 – 2.69 (m, 1H), 2.58 (t, *J* = 11.2 Hz, 1H), 2.32 (q, *J* = 7.5 Hz, 2H), 1.85 – 1.75 (m, 2H), 1.65 (dd, *J* = 14.3, 9.9 Hz, 1H), 1.54 (p, *J* = 7.5 Hz, 5H), 1.35 – 1.30 (m, 2H), 1.28 – 1.23 (m, 2H), 1.17 (s, 9H), 0.98 (s, 9H). ^13^C NMR (150 MHz, DMSO-*d*_6_) *δ* 172.61, 170.41, 169.81, 164.67, 160.59, 160.22, 159.74, 158.13, 158.08, 155.78, 154.14, 146.89, 144.54, 134.19, 134.11, 130.38, 129.41, 129.30, 128.56, 128.01, 125.49, 125.41, 125.04, 124.71, 124.68, 119.52, 119.34, 118.93, 118.80, 118.62, 118.55, 118.08, 117.06, 116.88, 116.72, 116.38, 108.96, 64.04, 62.86, 62.81, 62.65, 62.61, 55.19, 49.56, 43.64, 43.28, 40.66, 33.38, 31.68, 30.31, 29.50, 28.90, 28.48, 27.80, 27.69, 27.18, 25.69, 24.02. HRMS (ESI) calculated for C_54_H_63_Cl_2_F_2_N_8_O_6_S_2_ [M + H]^+^: 1091.3652, found: 1091.3627. UPLC-retention time: 6.718 min, purity >95%.

***N*-(5-(((5-(*tert*-butyl)oxazol-2-yl)methyl)thio)thiazol-2-yl)-1-(7-(4-((2*R*,3*S*,4*R*,5*S*)-3-(3-chloro-2-fluorophenyl)-4-(4-chloro-2-fluorophenyl)-4-cyano-5-neopentylpyrrolidine-2-carboxamido)-3-methoxybenzamido)heptanoyl)piperidine-4-carboxamide (5).** White solid; Yield: 60%. ^1^H NMR (600 MHz, DMSO-*d*_6_) *δ* 12.31 (s, 1H), 10.41 (s, 1H), 8.40 (t, *J* = 5.7 Hz, 1H), 8.32 (d, *J* = 8.4 Hz, 1H), 7.74 (t, *J* = 7.2 Hz, 1H), 7.62 – 7.46 (m, 4H), 7.44 – 7.30 (m, 4H), 6.71 (s, 1H), 4.60 (d, *J* = 7.4 Hz, 2H), 4.39 (d, *J* = 13.1 Hz, 2H), 4.05 (s, 2H), 3.93 (s, 5H), 3.25 (q, *J* = 6.5 Hz, 2H), 3.02 (t, *J* = 11.7 Hz, 1H), 2.77 – 2.69 (m, 1H), 2.63 – 2.54 (m, 1H), 2.31 (q, *J* = 7.2 Hz, 2H), 1.87 – 1.76 (m, 2H), 1.65 (dd, *J* = 14.2, 9.8 Hz, 1H), 1.52 (dq, *J* = 14.7, 7.1 Hz, 5H), 1.33 – 1.23 (m, 6H), 1.17 (s, 9H), 0.98 (s, 9H). ^13^C NMR (150 MHz, DMSO-*d*_6_) *δ* 172.61, 170.41, 169.85, 164.69, 160.60, 160.21, 159.74, 158.13, 158.08, 155.79, 154.15, 146.89, 144.54, 134.19, 134.12, 130.39, 130.36, 129.41, 129.31, 128.55, 128.01, 125.49, 125.41, 125.03, 124.71, 124.68, 119.52, 119.34, 118.93, 118.80, 118.61, 118.55, 118.09, 117.06, 116.88, 116.73, 116.39, 108.96, 64.04, 62.65, 62.61, 55.19, 49.55, 43.65, 43.28, 33.38, 31.68, 30.32, 29.50, 28.90, 28.52, 28.43, 27.97, 27.79, 27.68, 27.20, 25.80, 24.23. HRMS (ESI) calculated for C_55_H_65_Cl_2_F_2_N_8_O_6_S_2_ [M + H]^+^: 1105.3808, found: 1105.3797. UPLC-retention time: 7.047 min, purity >95%.

***N*-(5-(((5-(*tert*-butyl)oxazol-2-yl)methyl)thio)thiazol-2-yl)-1-(8-(4-((2*R*,3*S*,4*R*,5*S*)-3-(3-chloro-2-fluorophenyl)-4-(4-chloro-2-fluorophenyl)-4-cyano-5-neopentylpyrrolidine-2-carboxamido)-3-methoxybenzamido)octanoyl)piperidine-4-carboxamide (6).** White solid; Yield: 55%. ^1^H NMR (600 MHz, DMSO-*d*_6_) *δ* 12.31 (s, 1H), 10.41 (s, 1H), 8.40 (t, *J* = 5.7 Hz, 1H), 8.32 (d, *J* = 8.2 Hz, 1H), 7.74 (t, *J* = 7.4 Hz, 1H), 7.62 – 7.45 (m, 4H), 7.44 – 7.32 (m, 4H), 6.71 (s, 1H), 4.60 (d, *J* = 7.1 Hz, 2H), 4.42 – 4.34 (m, 2H), 4.05 (s, 2H), 3.98 – 3.88 (m, 5H), 3.25 (q, *J* = 6.8 Hz, 2H), 3.02 (t, *J* = 12.0 Hz, 1H), 2.78 – 2.69 (m, 1H), 2.63 – 2.55 (m, 1H), 2.30 (q, *J* = 7.1 Hz, 2H), 1.80 (t, *J* = 13.2 Hz, 2H), 1.65 (dd, *J* = 14.2, 9.9 Hz, 1H), 1.51 (dt, *J* = 21.5, 7.2 Hz, 5H), 1.31 – 1.23 (m, 8H), 1.17 (s, 9H), 0.98 (s, 9H). ^13^C NMR (150 MHz, DMSO-*d*_6_) *δ* 172.61, 170.41, 169.86, 164.68, 160.61, 160.21, 159.74, 158.13, 158.08, 155.78, 154.15, 146.89, 144.54, 134.19, 134.12, 130.38, 129.41, 129.31, 128.55, 128.01, 125.49, 125.41, 125.03, 124.71, 124.68, 119.52, 119.33, 118.92, 118.80, 118.62, 118.55, 118.08, 117.06, 116.88, 116.73, 116.39, 108.95, 64.04, 62.87, 62.82, 62.65, 62.61, 55.19, 49.56, 43.65, 43.28, 33.38, 31.69, 30.32, 29.50, 28.90, 28.58, 28.18, 28.16, 28.07, 27.80, 27.68, 27.20, 25.95, 25.86, 24.23. HRMS (ESI) calculated for C_56_H_67_Cl_2_F_2_N_8_O_6_S_2_ [M + H]^+^: 1119.3965, found: 1119.3949. UPLC-retention time: 6.451 min, purity >95%.

***N*-(5-(((5-(*tert*-butyl)oxazol-2-yl)methyl)thio)thiazol-2-yl)-1-(9-(4-((2*R*,3*S*,4*R*,5*S*)-3-(3-chloro-2-fluorophenyl)-4-(4-chloro-2-fluorophenyl)-4-cyano-5-neopentylpyrrolidine-2-carboxamido)-3-methoxybenzamido)nonanoyl)piperidine-4-carboxamide (7).** White solid; Yield: 60%. ^1^H NMR (500 MHz, DMSO-*d*_6_) *δ* 12.30 (s, 1H), 10.41 (s, 1H), 8.39 (t, *J* = 5.7 Hz, 1H), 8.32 (d, *J* = 8.4 Hz, 1H), 7.74 (ddd, *J* = 8.2, 6.5, 1.6 Hz, 1H), 7.60 – 7.47 (m, 4H), 7.43 – 7.33 (m, 4H), 6.71 (s, 1H), 4.60 (d, *J* = 7.2 Hz, 2H), 4.39 (d, *J* = 12.8 Hz, 2H), 4.05 (s, 2H), 3.96 (t, *J* = 11.2 Hz, 2H), 3.93 (s, 3H), 3.25 (q, *J* = 6.6 Hz, 2H), 3.02 (t, *J* = 11.5 Hz, 1H), 2.72 (ddt, *J* = 11.4, 7.6, 4.0 Hz, 1H), 2.63 – 2.54 (m, 1H), 2.29 (q, *J* = 7.5 Hz, 2H), 1.85 – 1.76 (m, 2H), 1.65 (dd, *J* = 14.1, 9.8 Hz, 1H), 1.51 (dt, *J* = 20.9, 7.1 Hz, 5H), 1.28 (d, *J* = 14.6 Hz, 10H), 1.17 (s, 9H), 0.98 (s, 9H). ^13^C NMR (125 MHz, DMSO-*d*_6_) *δ* 173.67, 171.47, 170.93, 165.75, 161.67, 161.28, 159.20, 158.98, 157.03, 155.07, 147.97, 145.60, 135.27, 135.18, 131.45, 130.48, 130.39, 129.63, 129.09, 126.57, 126.47, 126.10, 125.77, 120.59, 120.40, 120.02, 119.87, 119.69, 119.62, 119.17, 118.15, 117.93, 117.79, 117.47, 110.03, 65.12, 63.95, 63.73, 63.68, 56.26, 50.63, 44.73, 44.36, 41.73, 40.77, 34.46, 32.78, 31.38, 30.56, 29.97, 29.66, 29.34, 29.26, 29.22, 28.87, 28.75, 28.27, 26.98, 25.36. HRMS (ESI) calculated for C_57_H_69_Cl_2_F_2_N_8_O_6_S_2_ [M + H]^+^: 1133.4121, found: 1133.4111. UPLC-retention time: 6.953 min, purity >95%.

***N*-(5-(((5-(*tert*-butyl)oxazol-2-yl)methyl)thio)thiazol-2-yl)-1-(10-(4-((2*R*,3*S*,4*R*,5*S*)-3-(3-chloro-2-fluorophenyl)-4-(4-chloro-2-fluorophenyl)-4-cyano-5-neopentylpyrrolidine-2-carboxamido)-3-methoxybenzamido)decanoyl)piperidine-4-carboxamide (8).** White solid; Yield: 65%. ^1^H NMR (600 MHz, DMSO-*d*_6_) *δ* 12.31 (s, 1H), 10.41 (s, 1H), 8.40 (t, *J* = 5.7 Hz, 1H), 8.32 (d, *J* = 8.2 Hz, 1H), 7.74 (t, *J* = 7.2 Hz, 1H), 7.60 – 7.47 (m, 4H), 7.43 – 7.33 (m, 4H), 6.71 (s, 1H), 4.59 (d, *J* = 7.1 Hz, 2H), 4.39 (dd, *J* = 13.2, 4.5 Hz, 2H), 4.05 (s, 2H), 3.98 – 3.89 (m, 5H), 3.25 (q, *J* = 6.7 Hz, 2H), 3.02 (t, *J* = 11.6 Hz, 1H), 2.77 – 2.69 (m, 1H), 2.58 (t, *J* = 11.3 Hz, 1H), 2.29 (q, *J* = 7.6 Hz, 2H), 1.87 – 1.76 (m, 2H), 1.65 (dd, *J* = 14.3, 9.7 Hz, 1H), 1.55 – 1.45 (m, 5H), 1.32 – 1.25 (m, 12H), 1.17 (s, 9H), 0.98 (s, 9H). ^13^C NMR (150 MHz, DMSO-*d*_6_) *δ* 172.61, 170.41, 169.86, 164.67, 160.61, 160.21, 159.74, 158.13, 158.08, 155.78, 154.15, 146.88, 144.54, 134.19, 134.11, 130.38, 130.35, 129.41, 129.31, 129.04, 128.55, 128.02, 125.49, 125.41, 125.04, 124.71, 124.68, 119.52, 119.33, 118.92, 118.80, 118.62, 118.55, 118.07, 117.06, 116.88, 116.72, 116.38, 108.95, 64.04, 62.85, 62.65, 62.61, 55.19, 49.56, 43.65, 43.28, 33.38, 31.70, 30.32, 29.50, 28.90, 28.59, 28.49, 28.43, 28.34, 28.28, 28.20, 28.18, 28.15, 27.98, 27.80, 27.68, 27.20, 25.92, 24.29. HRMS (ESI) calculated for C_58_H_71_Cl_2_F_2_N_8_O_6_S_2_ [M + H]^+^: 1147.4278, found: 1147.4246. UPLC-retention time: 6.928 min, purity >95%.

***N*-(5-(((5-(*tert*-butyl)oxazol-2-yl)methyl)thio)thiazol-2-yl)-1-(11-(4-((2*R*,3*S*,4*R*,5*S*)-3-(3-chloro-2-fluorophenyl)-4-(4-chloro-2-fluorophenyl)-4-cyano-5-neopentylpyrrolidine-2-carboxamido)-3-methoxybenzamido)undecanoyl)piperidine-4-carboxamide (9).** White solid; Yield: 60%. ^1^H NMR (600 MHz, DMSO-*d*_6_) *δ* 12.31 (s, 1H), 10.41 (s, 1H), 8.39 (t, *J* = 5.9 Hz, 1H), 8.32 (d, *J* = 8.4 Hz, 1H), 7.74 (t, *J* = 7.4 Hz, 1H), 7.61 – 7.52 (m, 3H), 7.48 (d, *J* = 8.4 Hz, 1H), 7.42 – 7.33 (m, 4H), 6.71 (s, 1H), 4.60 (d, *J* = 7.6 Hz, 2H), 4.43 – 4.35 (m, 2H), 4.05 (s, 2H), 3.96 (t, *J* = 11.0 Hz, 1H), 3.92 (s, 3H), 3.90 (d, *J* = 13.6 Hz, 1H), 3.25 (q, *J* = 6.4 Hz, 2H), 3.02 (t, *J* = 12.8 Hz, 1H), 2.77 – 2.69 (m, 1H), 2.58 (t, *J* = 12.5 Hz, 1H), 2.29 (q, *J* = 7.6 Hz, 2H), 1.81 (t, *J* = 14.0 Hz, 2H), 1.65 (dd, *J* = 14.4, 9.8 Hz, 1H), 1.55 – 1.49 (m, 3H), 1.47 (t, *J* = 7.1 Hz, 2H), 1.30 – 1.23 (m, 14H), 1.17 (d, *J* = 2.1 Hz, 9H), 0.98 (d, *J* = 1.9 Hz, 9H). ^13^C NMR (150 MHz, DMSO-*d*_6_) *δ* 170.42, 169.86, 164.67, 160.63, 160.22, 159.75, 158.14, 158.09, 155.80, 154.16, 146.89, 144.55, 134.20, 134.13, 130.39, 130.37, 129.42, 129.32, 129.05, 128.56, 128.02, 125.51, 125.42, 125.05, 124.72, 124.68, 119.53, 119.34, 118.94, 118.81, 118.62, 118.56, 118.08, 117.07, 116.89, 116.73, 116.39, 108.96, 64.06, 62.87, 62.66, 62.62, 55.19, 49.57, 43.66, 43.29, 40.67, 39.70, 33.39, 31.71, 30.70, 30.32, 29.50, 28.91, 28.60, 28.50, 28.45, 28.39, 28.37, 28.32, 28.25, 28.22, 28.16, 28.11, 28.00, 27.81, 27.69, 27.21, 25.94, 24.31. HRMS (ESI) calculated for C_59_H_73_Cl_2_F_2_N_8_O_6_S_2_ [M + H]^+^: 1161.4434, found: 1161.4417. UPLC-retention time: 7.370 min, purity >95%.

***N*-(5-(((5-(*tert*-butyl)oxazol-2-yl)methyl)thio)thiazol-2-yl)-1-(12-(4-((2*R*,3*S*,4*R*,5*S*)-3-(3-chloro-2-fluorophenyl)-4-(4-chloro-2-fluorophenyl)-4-cyano-5-neopentylpyrrolidine-2-carboxamido)-3-methoxybenzamido)dodecanoyl)piperidine-4-carboxamide (10).** White solid; Yield: 55%. ^1^H NMR (600 MHz, DMSO-*d*_6_) *δ* 12.31 (s, 1H), 10.41 (s, 1H), 8.39 (t, *J* = 5.7 Hz, 1H), 8.32 (d, *J* = 8.4 Hz, 1H), 7.74 (t, *J* = 7.2 Hz, 1H), 7.61 – 7.45 (m, 4H), 7.42 – 7.32 (m, 4H), 6.71 (s, 1H), 4.60 (d, *J* = 7.4 Hz, 2H), 4.39 (dd, *J* = 12.8, 7.6 Hz, 2H), 4.05 (s, 2H), 3.98 – 3.88 (m, 5H), 3.25 (q, *J* = 6.7 Hz, 2H), 3.02 (t, *J* = 11.7 Hz, 1H), 2.77 – 2.69 (m, 1H), 2.58 (t, *J* = 11.3 Hz, 1H), 2.34 – 2.23 (m, 2H), 1.86 – 1.76 (m, 2H), 1.65 (dd, *J* = 14.3, 9.7 Hz, 1H), 1.50 (dt, *J* = 27.5, 7.2 Hz, 5H), 1.27 (d, *J* = 20.7 Hz, 16H), 1.17 (s, 9H), 0.98 (s, 9H). ^13^C NMR (150 MHz, DMSO-*d*_6_) δ 172.62, 170.42, 169.87, 164.67, 160.65, 160.22, 159.75, 158.14, 155.80, 154.16, 146.89, 144.54, 134.20, 134.13, 130.40, 129.42, 129.32, 128.56, 125.51, 125.42, 125.05, 124.72, 119.53, 119.34, 118.94, 118.81, 118.63, 118.56, 118.07, 117.07, 116.89, 116.73, 116.39, 108.95, 64.06, 62.89, 62.67, 55.19, 49.56, 43.67, 43.29, 40.67, 33.39, 31.71, 30.33, 29.50, 28.91, 28.59, 28.41, 28.22, 27.82, 27.69, 27.21, 25.93, 24.30. HRMS (ESI) calculated for C_60_H_75_Cl_2_F_2_N_8_O_6_S_2_ [M + H]^+^: 1175.4591, found: 1175.4566. UPLC-retention time: 7.551 min, purity >95%.

***General Procedure for Synthesis of Compound*** ***11-14****.*

DIPEA (5 eq.) and HATU (1.2 eq.) were added to a solution of compound **1** (381 mg, 1 mmol) and a series of linear acids containing varying numbers of PEG, referred to as compound **47** (1.1 eq.) in DMF (2 mL). After 30 min at rt, the mixture was subject to prep-HPLC to aﬀord the intermediates in 80−90% yields. A solution of the intermediate in 1:1 TFA/DCM was stirred at rt for 30 min. The solvents were evaporated under reduced pressure to give the corresponding deprotected intermediate **51** (TFA salt) that was used in the following reactions without further puriﬁcation (50% - 65% yields).

DIPEA (5 eq.) and HATU (1.2 eq.) were added to a solution of compound **51** (61.5 mg, 0.1 mmol) and compound **2** (1.1 eq.) in DMF (2 mL). After 30 min at rt, the mixture was subject to prep-HPLC to aﬀord compound **11**. Following the procedures used to prepare compound **11**, compounds **12**−**14** with diﬀerent numbers of PEG chain lengths were obtained by the same methods.

***N*-(5-(((5-(*tert*-butyl)oxazol-2-yl)methyl)thio)thiazol-2-yl)-1-(3-(2-(4-((2*R*,3*S*,4*R*,5*S*)-3-(3-chloro-2-fluorophenyl)-4-(4-chloro-2-fluorophenyl)-4-cyano-5-neopentylpyrrolidine-2-carboxamido)-3-methoxybenzamido)ethoxy)propanoyl)piperidine-4-carboxamide (11).** White solid; Yield: 60%. ^1^H NMR (600 MHz, DMSO-*d*_6_) *δ* 12.31 (s, 1H), 10.42 (s, 1H), 8.46 (t, *J* = 6.1 Hz, 1H), 8.33 (d, *J* = 8.4 Hz, 1H), 7.74 (t, *J* = 7.5 Hz, 1H), 7.60 – 7.47 (m, 4H), 7.44 – 7.32 (m, 4H), 6.71 (s, 1H), 4.60 (s, 2H), 4.38 (d, *J* = 6.8 Hz, 2H), 4.05 (s, 2H), 3.99 – 3.93 (m, 2H), 3.93 (s, 3H), 3.67 (t, *J* = 6.7 Hz, 2H), 3.53 (t, *J* = 6.1 Hz, 2H), 3.43 (d, *J* = 6.5 Hz, 2H), 3.08 – 2.98 (m, 1H), 2.72 (t, *J* = 11.3 Hz, 1H), 2.61 (dd, *J* = 10.9, 5.2 Hz, 2H), 1.80 (t, *J* = 12.5 Hz, 2H), 1.66 (d, *J* = 9.8 Hz, 1H), 1.57 (t, *J* = 12.4 Hz, 1H), 1.41 (t, *J* = 12.5 Hz, 1H), 1.31 – 1.22 (m, 2H), 1.16 (s, 9H), 0.97 (s, 9H). ^13^C NMR (150 MHz, DMSO-*d*_6_) *δ* 172.60, 170.44, 168.09, 164.92, 160.63, 160.22, 159.74, 158.14, 158.08, 155.79, 154.15, 146.89, 144.54, 134.19, 134.12, 130.39, 130.36, 129.42, 129.00, 128.70, 128.01, 125.49, 125.40, 125.03, 124.71, 119.51, 119.45, 118.93, 118.81, 118.61, 118.55, 118.07, 117.06, 116.88, 116.73, 116.40, 109.02, 68.11, 65.81, 65.78, 64.04, 62.86, 62.65, 62.61, 55.20, 49.56, 43.68, 43.28, 40.59, 39.71, 39.44, 33.39, 32.12, 30.31, 29.49, 28.90, 27.67. HRMS (ESI) calculated for C_53_H_61_Cl_2_F_2_N_8_O_7_S_2_ [M + H]^+^: 1093.3444, found: 1093.3407. UPLC-retention time: 6.415 min, purity >95%.

***N*-(5-(((5-(*tert*-butyl)oxazol-2-yl)methyl)thio)thiazol-2-yl)-1-(3-(2-(2-(4-((2*R*,3*S*,4*R*,5*S*)-3-(3-chloro-2-fluorophenyl)-4-(4-chloro-2-fluorophenyl)-4-cyano-5-neopentylpyrrolidine-2-carboxamido)-3-methoxybenzamido)ethoxy)ethoxy)propanoyl)piperidine-4-carboxamide (12).** White solid; Yield: 50%. ^1^H NMR (600 MHz, DMSO-*d*_6_) *δ* 12.31 (s, 1H), 10.42 (s, 1H), 8.50 (t, *J* = 5.7 Hz, 1H), 8.33 (d, *J* = 8.4 Hz, 1H), 7.74 (t, *J* = 7.2 Hz, 1H), 7.62 – 7.48 (m, 4H), 7.44 – 7.29 (m, 4H), 6.71 (s, 1H), 4.60 (d, *J* = 6.8 Hz, 2H), 4.38 (d, *J* = 12.5 Hz, 2H), 4.05 (s, 2H), 3.99 – 3.88 (m, 5H), 3.63 (t, *J* = 6.7 Hz, 2H), 3.56 – 3.50 (m, 6H), 3.42 (d, *J* = 5.7 Hz, 2H), 3.01 (t, *J* = 12.0 Hz, 1H), 2.75 – 2.69 (m, 1H), 2.61 – 2.53 (m, 2H), 1.80 (t, *J* = 11.4 Hz, 2H), 1.65 (dd, *J* = 14.4, 9.8 Hz, 1H), 1.62 – 1.52 (m, 1H), 1.45 – 1.36 (m, 1H), 1.29 – 1.23 (m, 2H), 1.17 (s, 9H), 0.98 (s, 9H). ^13^C NMR (150 MHz, DMSO-*d*_6_) *δ* 172.62, 170.43, 168.00, 164.95, 160.65, 159.75, 158.14, 158.08, 155.80, 154.16, 146.89, 144.54, 134.12, 130.36, 129.42, 129.00, 128.68, 128.01, 125.49, 125.05, 124.72, 124.68, 119.52, 119.48, 118.94, 118.81, 118.61, 118.55, 118.06, 117.06, 116.89, 116.73, 116.41, 109.00, 69.04, 68.41, 66.26, 64.04, 62.86, 62.65, 62.62, 55.20, 49.54, 43.72, 43.29, 40.61, 33.39, 32.16, 30.32, 29.50, 28.91, 28.11, 27.72, 27.56, 27.12. HRMS (ESI) calculated for C_55_H_65_Cl_2_F_2_N_8_O_8_S_2_ [M + H]^+^: 1137.3706, found: 1137.3675. UPLC-retention time: 6.189 min, purity >95%.

***N*-(5-(((5-(*tert*-butyl)oxazol-2-yl)methyl)thio)thiazol-2-yl)-1-(1-(4-((2*R*,3*S*,4*R*,5*S*)-3-(3-chloro-2-fluorophenyl)-4-(4-chloro-2-fluorophenyl)-4-cyano-5-neopentylpyrrolidine-2-carboxamido)-3-methoxyphenyl)-1-oxo-5,8,11-trioxa-2-azatetradecan-14-oyl)piperidine-4-carboxamide (13).** White solid; Yield: 55%. ^1^H NMR (600 MHz, DMSO-*d*_6_) *δ* 12.29 (s, 1H), 10.41 (s, 1H), 8.49 (s, 1H), 8.32 (s, 1H), 7.73 (s, 1H), 7.60 – 7.47 (m, 4H), 7.37 (d, *J* = 23.1 Hz, 4H), 6.71 (s, 1H), 4.59 (s, 2H), 4.38 (s, 2H), 4.04 (s, 2H), 3.95 (s, 2H), 3.92 (s, 3H), 3.60 (s, 2H), 3.51 (d, *J* = 16.9 Hz, 10H), 3.41 (s, 2H), 3.01 (t, *J* = 14.8 Hz, 1H), 2.71 (s, 1H), 2.58 (s, 2H), 1.79 (s, 2H), 1.65 (d, *J* = 16.0 Hz, 1H), 1.55 (d, *J* = 12.5 Hz, 1H), 1.39 (d, *J* = 12.7 Hz, 1H), 1.29 – 1.21 (m, 2H), 1.17 (s, 9H), 0.98 (s, 9H). ^13^C NMR (150 MHz, DMSO-*d*_6_) *δ* 173.66, 171.50, 169.06, 166.01, 161.68, 161.29, 160.81, 159.20, 159.15, 156.86, 155.22, 147.95, 145.61, 135.26, 135.18, 131.42, 130.48, 130.06, 129.76, 129.08, 126.56, 126.46, 126.10, 125.78, 120.58, 120.53, 120.00, 119.87, 119.68, 119.62, 119.15, 118.13, 117.95, 117.79, 117.47, 110.06, 70.24, 70.20, 70.14, 69.46, 67.32, 65.11, 63.86, 63.73, 63.68, 56.27, 50.62, 44.79, 44.35, 41.67, 40.75, 34.45, 33.23, 31.39, 30.56, 29.97, 28.79, 28.75, 28.17. HRMS (ESI) calculated for C_57_H_69_Cl_2_F_2_N_8_O_9_S_2_ [M + H]^+^: 1181.3969, found: 1181.3917. UPLC-retention time: 6.147 min, purity >95%.

***N*-(5-(((5-(*tert*-butyl)oxazol-2-yl)methyl)thio)thiazol-2-yl)-1-(1-(4-((2*R*,3*S*,4*R*,5*S*)-3-(3-chloro-2-fluorophenyl)-4-(4-chloro-2-fluorophenyl)-4-cyano-5-neopentylpyrrolidine-2-carboxamido)-3-methoxyphenyl)-1-oxo-5,8,11,14-tetraoxa-2-azaheptadecan-17-oyl)piperidine-4-carboxamide** **(14).** White solid; Yield: 55%. ^1^H NMR (600 MHz, DMSO-*d*_6_) *δ* 12.31 (s, 1H), 10.42 (s, 1H), 8.50 (t, *J* = 6.0 Hz, 1H), 8.33 (d, *J* = 8.4 Hz, 1H), 7.74 (t, *J* = 7.2 Hz, 1H), 7.63 – 7.48 (m, 4H), 7.44 – 7.31 (m, 4H), 6.71 (d, *J* = 2.0 Hz, 1H), 4.60 (d, *J* = 6.8 Hz, 2H), 4.44 – 4.34 (m, 2H), 4.05 (s, 2H), 3.99 – 3.93 (m, 2H), 3.92 (s, 3H), 3.61 (t, *J* = 6.8 Hz, 2H), 3.53 (d, *J* = 5.7 Hz, 6H), 3.49 (d, *J* = 8.7 Hz, 8H), 3.43 (t, *J* = 6.0 Hz, 2H), 3.02 (t, *J* = 12.4 Hz, 1H), 2.77 – 2.68 (m, 1H), 2.60 (q, *J* = 7.6 Hz, 2H), 1.80 (t, *J* = 12.4 Hz, 2H), 1.65 (dd, *J* = 14.3, 9.7 Hz, 1H), 1.60 – 1.52 (m, 1H), 1.40 (p, *J* = 12.8 Hz, 1H), 1.29 – 1.23 (m, 2H), 1.17 (s, 9H), 0.98 (s, 9H). ^13^C NMR (150 MHz, DMSO-*d*_6_) *δ* 173.68, 171.50, 169.07, 166.00, 161.73, 161.28, 160.81, 159.20, 159.14, 156.86, 155.22, 147.95, 145.60, 135.26, 135.18, 131.45, 131.42, 130.48, 130.11, 130.06, 129.74, 129.07, 126.55, 126.46, 126.10, 125.77, 125.74, 120.57, 120.53, 120.00, 119.87, 119.67, 119.61, 119.10, 118.12, 117.95, 117.79, 117.47, 110.06, 70.25, 70.21, 70.12, 69.46, 67.32, 65.10, 63.91, 63.71, 63.68, 56.26, 50.61, 44.80, 44.35, 41.68, 40.75, 34.45, 33.23, 31.38, 30.56, 29.97, 29.50, 29.31, 29.05, 28.81, 28.74, 28.17, 27.02. HRMS (ESI) calculated for C_59_H_73_Cl_2_F_2_N_8_O_10_S_2_ [M + H]^+^: 1225.4231, found: 1225.4183. UPLC-retention time: 6.461 min, purity >95%.

***General Procedure for Synthesis of Compounds*** ***15-17****.*

To a solution of compound **3** (100 mg, 0.17 mmol) in DMF (2 mL) was added NaH (2 eq, 60%) at 0 °C for 0.5 h, followed by addition of **55** (1.2 eq) in DMF (2 mL) dropwise. After stirring at room temperature for 3 h, the reaction was quenched with water (30 mL) and the mixture was subject to prep-HPLC to aﬀord the intermediates in 30% yield. A solution of the intermediate in 1:1 TFA/DCM was stirred at rt for 30 min. The solvents were evaporated under reduced pressure to give the corresponding deprotected intermediate **56** (TFA salt) that was used in the following reactions without further puriﬁcation (35% yield).

DIPEA (5 eq.) and HATU (1.2 eq.) were added to a solution of compound **56** (32 mg, 0.05 mmol) and **51** (1.1 eq.) in DMF (2 mL). After 30 min at rt, the mixture was subject to prep-HPLC to aﬀord compound **15**. Following the procedures used to prepare compound **15**, compound **16-17** with diﬀerent numbers of PEG chain length was obtained by the same method.

**1-(3-(2-(2-(4-((4*R*,5*S*)-4,5-bis(4-chlorophenyl)-2-(2-isopropoxy-4-methoxyphenyl)-4,5-dihydro-1*H*-imidazole-1-carbonyl)-2-oxopiperazin-1-yl)acetamido)ethoxy)propanoyl)-*N*-(5-(((5-(*tert*-butyl)oxazol-2-yl)methyl)thio)thiazol-2-yl)piperidine-4-carboxamide (15)** White solid; Yield: 55%. 1H NMR (500 MHz, DMSO-*d*_6_) *δ* 8.41 (s, 1H), 7.94 (t, J = 5.6 Hz, 1H), 7.53 (d, J = 9.2 Hz, 1H), 7.38 (s, 1H), 7.13 (dd, J = 17.6, 8.5 Hz, 4H), 7.01 (dd, J = 31.7, 8.3 Hz, 4H), 6.71 (s, 1H), 6.61 (d, J = 7.2 Hz, 2H), 5.69 – 5.56 (m, 2H), 4.72 (p, J = 6.0 Hz, 1H), 4.05 (s, 2H), 3.94 – 3.86 (m, 2H), 3.83 (s, 3H), 3.75 – 3.67 (m, 2H), 3.59 (q, J = 6.6 Hz, 4H), 3.37 (d, J = 6.0 Hz, 4H), 3.21 – 3.15 (m, 3H), 3.00 (d, J = 6.4 Hz, 2H), 2.72 (d, J = 22.9 Hz, 1H), 2.56 (d, J = 7.0 Hz, 2H), 2.04 – 1.94 (m, 2H), 1.84 – 1.74 (m, 2H), 1.23 (d, J = 6.0 Hz, 6H), 1.17 (s, 9H).HRMS (ESI) calculated for C_54_H_64_Cl_2_N_9_O_9_S_2_ [M + H]^+^: 1116.3640, found: 1116.3610. UPLC-retention time: 5.638 min, purity >95%.

**1-(3-(2-(2-(2-(4-((4*R*,5*S*)-4,5-bis(4-chlorophenyl)-2-(2-isopropoxy-4-methoxyphenyl)-4,5-dihydro-1*H*-imidazole-1-carbonyl)-2-oxopiperazin-1-yl)acetamido)ethoxy)ethoxy)propanoyl)-*N*-(5-(((5-(*tert*-butyl)oxazol-2-yl)methyl)thio)thiazol-2-yl)piperidine-4-carboxamide (16)**. White solid; Yield: 60%. 1H NMR (500 MHz, DMSO-*d*_6_) *δ* 8.42 (s, 1H), 7.95 (t, J = 5.7 Hz, 1H), 7.53 (d, J = 9.0 Hz, 1H), 7.38 (s, 1H), 7.13 (dd, J = 17.9, 8.5 Hz, 4H), 7.04 (d, J = 8.5 Hz, 2H), 6.97 (d, J = 8.4 Hz, 2H), 6.71 (s, 1H), 6.61 (d, J = 7.2 Hz, 2H), 5.70 – 5.53 (m, 2H), 4.72 (q, J = 6.0 Hz, 1H), 4.05 (s, 2H), 3.83 (s, 3H), 3.75 – 3.65 (m, 2H), 3.61 (t, J = 6.8 Hz, 3H), 3.48 (s, 4H), 3.38 (t, J = 6.0 Hz, 6H), 3.18 (q, J = 6.0 Hz, 4H), 3.00 (t, J = 6.1 Hz, 2H), 2.79 – 2.69 (m, 1H), 2.61 – 2.53 (m, 2H), 1.99 (dd, J = 10.5, 6.8 Hz, 2H), 1.79 (s, 2H), 1.23 (s, 6H), 1.17 (s, 9H). HRMS (ESI) calculated for C_56_H_68_Cl_2_N_9_O_10_S_2_ [M + H]^+^: 1160.3902, found: 1160.3887. UPLC-retention time: 5.701 min, purity >95%.

**1-(1-(4-((4*R*,5*S*)-4,5-bis(4-chlorophenyl)-2-(2-isopropoxy-4-methoxyphenyl)-4,5-dihydro-1*H*-imidazole-1-carbonyl)-2-oxopiperazin-1-yl)-2-oxo-6,9,12-trioxa-3-azapentadecan-15-oyl)-*N*-(5-(((5-(*tert*-butyl)oxazol-2-yl)methyl)thio)thiazol-2-yl)piperidine-4-carboxamide (17).** White solid; Yield: 65%. 1H NMR (500 MHz, DMSO-*d*_6_) *δ* 8.39 (s, 1H), 7.95 (t, J = 5.5 Hz, 1H), 7.53 (d, J = 9.2 Hz, 1H), 7.38 (s, 1H), 7.13 (dd, J = 17.9, 8.6 Hz, 4H), 7.01 (dd, J = 31.9, 8.5 Hz, 4H), 6.71 (s, 1H), 6.61 (d, J = 7.1 Hz, 2H), 5.67 – 5.56 (m, 2H), 4.72 (p, J = 6.0 Hz, 1H), 4.05 (s, 2H), 3.83 (s, 4H), 3.74 – 3.67 (m, 2H), 3.62 (s, 2H), 3.49 (s, 10H), 3.38 (t, J = 5.9 Hz, 6H), 3.18 (d, J = 5.8 Hz, 2H), 2.99 (t, J = 6.2 Hz, 2H), 2.76 – 2.69 (m, 1H), 2.59 (d, J = 6.7 Hz, 2H), 2.08 – 1.94 (m, 2H), 1.79 (s, 2H), 1.23 (d, J = 5.5 Hz, 6H), 1.17 (s, 9H). HRMS (ESI) calculated for C_58_H_72_Cl_2_N_9_O_11_S_2_ [M + H]^+^: 1204.4164, found: 1204.4107. UPLC-retention time: 4.647 min, purity >95%.

***General Procedure for Synthesis of Compound*** ***18-19****.*

DIPEA (5 eq.) and HATU (1.2 eq.) were added to a solution of compound **1** (381 mg, 1 mmol) and compound **32** (1.1 eq.) in DMF (2 mL). After 30 min at rt, the mixture was subject to prep-HPLC to aﬀord the intermediate in 80% yield. A solution of the intermediate in 1:1 TFA/DCM was stirred at rt for 30 min. The solvents were evaporated under reduced pressure to give the corresponding deprotected intermediate **57** (TFA salt) that was used in the following reactions without further puriﬁcation (60% yield). ^1^H NMR (500 MHz, DMSO-*d*_6_) *δ* 8.37 (s, 1H), 7.39 (s, 1H), 6.72 (s, 1H), 4.39 (d, J = 11.1 Hz, 1H), 4.05 (s, 3H), 3.23 (s, 2H), 2.97 – 2.56 (m, 6H), 1.89 – 1.34 (m, 9H), 1.18 (s, 9H). HRMS (ESI) calculated for C_23_H_34_N_5_O_3_S_2_ [M + H]^+^: 492.2098, found: 492.2058.

K_2_CO_3_ (3 eq.) was added to a solution of the intermediate **57** (49.1 mg, 0.1 mmol) and a series of linear acids containing varying numbers of PEG, referred to as compound **58** (1.2 eq.) in CH_3_CN was added. Then, the mixture was reﬂuxed overnight. After the solvents were evaporated under reduced pressure to aﬀord the corresponding crude that was subject to prep-HPLC to aﬀord the intermediates in 75% yields. A solution of the intermediate in 1:1 TFA/DCM was stirred at rt for 30 min. The solvents were evaporated under reduced pressure to give the corresponding deprotected intermediate **60** (TFA salt) that was used in the following reactions without further puriﬁcation (65% yield).

DIPEA (5 eq.) and HATU (1.2 eq.) were added to a solution of compound **60** (61.5 mg, 0.1 mmol) and compound **2** (1.1 eq.) in DMF (2 mL). After 30 min at rt, the mixture was subject to prep-HPLC to aﬀord compound **18**. Following the procedures used to prepare compound **18**, compound **19** with diﬀerent numbers of PEG chain length was obtained by the same method.

***N*-(5-(((5-(*tert*-butyl)oxazol-2-yl)methyl)thio)thiazol-2-yl)-1-(1-(2-(2-(4-((2*R*,3*S*,4*R*,5*S*)-3-(3-chloro-2-fluorophenyl)-4-(4-chloro-2-fluorophenyl)-4-cyano-5-neopentylpyrrolidine-2-carboxamido)-3-methoxybenzamido)ethoxy)ethyl)piperidine-4-carbonyl)piperidine-4-carboxamide** **(18).** White solid; Yield: 60%. ^1^H NMR (400 MHz, DMSO-*d*_6_) *δ* 12.32 (s, 1H), 10.42 (s, 1H), 8.52 (t, *J* = 5.7 Hz, 1H), 8.33 (d, *J* = 8.4 Hz, 1H), 7.74 (t, *J* = 6.5 Hz, 1H), 7.61 – 7.48 (m, 4H), 7.43 – 7.32 (m, 4H), 6.71 (s, 1H), 4.60 (d, *J* = 5.6 Hz, 2H), 4.38 (d, *J* = 11.7 Hz, 2H), 4.05 (s, 2H), 3.93 (s, 5H), 3.56 (dt, *J* = 20.4, 5.6 Hz, 4H), 3.43 (q, *J* = 5.6 Hz, 2H), 3.01 (t, *J* = 10.7 Hz, 3H), 2.77 – 2.54 (m, 5H), 2.24 (d, *J* = 12.1 Hz, 2H), 1.88 – 1.77 (m, 2H), 1.69 – 1.49 (m, 6H), 1.30 – 1.22 (m, 2H), 1.18 (s, 9H), 0.98 (s, 9H). ^13^C NMR (125 MHz, DMSO-*d*_6_) *δ* 173.64, 172.69, 171.50, 166.03, 163.98, 161.66, 161.29, 159.20, 158.99, 157.03, 155.06, 147.97, 145.60, 135.18, 131.45, 130.48, 130.07, 129.77, 129.07, 126.54, 126.44, 126.10, 125.76, 120.59, 120.53, 120.01, 119.86, 119.68, 119.60, 119.19, 118.14, 117.93, 117.79, 117.49, 110.08, 69.35, 67.79, 65.10, 63.68, 57.44, 56.31, 53.13, 50.63, 44.49, 44.36, 41.79, 40.92, 40.59, 40.50, 40.42, 40.33, 40.25, 40.16, 40.09, 40.00, 39.83, 39.66, 39.60, 39.50, 37.14, 34.47, 31.39, 30.56, 29.97, 29.56, 29.11, 28.76, 28.32, 0.57. HRMS (ESI) calculated for C_58_H_70_Cl_2_F_2_N_9_O_7_S_2_ [M + H]^+^: 1176.4179, found: 1176.4112. UPLC-retention time: 4.912 min, purity >95%.

***N*-(5-(((5-(*tert*-butyl)oxazol-2-yl)methyl)thio)thiazol-2-yl)-1-(1-(2-(2-(2-(4-((2*R*,3*S*,4*R*,5*S*)-3-(3-chloro-2-fluorophenyl)-4-(4-chloro-2-fluorophenyl)-4-cyano-5-neopentylpyrrolidine-2-carboxamido)-3-methoxybenzamido)ethoxy)ethoxy)ethyl)piperidine-4-carbonyl)piperidine-4-carboxamide (19)**. White solid; Yield: 55%. ^1^H NMR (500 MHz, DMSO-*d*_6_) *δ* 12.32 (s, 1H), 10.41 (s, 1H), 8.53 (t, *J* = 5.6 Hz, 1H), 8.33 (d, *J* = 8.5 Hz, 1H), 7.74 (t, *J* = 6.6 Hz, 1H), 7.60 – 7.49 (m, 4H), 7.42 – 7.32 (m, 4H), 6.71 (s, 1H), 4.60 (d, *J* = 6.4 Hz, 2H), 4.39 (d, *J* = 11.4 Hz, 2H), 4.05 (s, 2H), 3.97 (d, *J* = 11.4 Hz, 2H), 3.93 (s, 3H), 3.62 (t, *J* = 5.6 Hz, 2H), 3.57 – 3.54 (m, 5H), 3.43 (q, *J* = 6.1 Hz, 2H), 3.18 – 3.11 (m, 2H), 3.05 (t, *J* = 12.2 Hz, 1H), 2.78 (d, *J* = 13.0 Hz, 2H), 2.76 – 2.68 (m, 2H), 2.57 (d, *J* = 22.9 Hz, 2H), 2.47 (s, 2H), 1.88 – 1.78 (m, 2H), 1.65 (dd, *J* = 14.5, 9.9 Hz, 4H), 1.54 (d, *J* = 11.0 Hz, 1H), 1.41 (d, *J* = 11.0 Hz, 1H), 1.25 (d, *J* = 13.3 Hz, 2H), 1.17 (s, 9H), 0.98 (s, 9H). 13C NMR (125 MHz, DMSO-*d*_6_) *δ* 173.63, 172.33, 171.52, 166.02, 163.68, 161.65, 161.30, 159.20, 158.98, 155.06, 147.97, 145.61, 135.28, 135.18, 131.47, 130.49, 130.06, 129.77, 129.09, 126.56, 126.45, 126.12, 125.78, 120.58, 120.54, 120.01, 119.86, 119.68, 119.19, 118.15, 117.93, 117.79, 117.50, 110.09, 70.07, 69.96, 69.42, 67.04, 65.12, 63.95, 63.73, 63.68, 56.85, 56.31, 52.68, 50.61, 44.51, 44.36, 41.75, 36.42, 34.47, 31.39, 30.56, 29.97, 29.05, 28.76, 28.31. HRMS (ESI) calculated for C_60_H_74_Cl_2_F_2_N_9_O_8_S_2_ [M + H]^+^: 1220.4414, found: 1220.4402. UPLC-retention time: 4.806 min, purity >95%.

***General Procedure for Synthesis of Compound*** ***20****.*

K_2_CO_3_ (3 eq.) were added to a solution of **1** (38.1 mg, 0.1 mmol) and compound **55** (1.2 eq.) in DMF was added. After LC−MS demonstrated the full conversion of starting materials, H_2_O was added and the mixture was extracted with EtOAc three times. The combined organic layers were washed with brine, and then dried over anhydrous Na_2_SO_4_. The solvent was removed under reduced pressure to aﬀord the corresponding crude that was subject to prep-HPLC to aﬀord the intermediates. The desired compound **62** was obtained by deprotection with TFA in DCM in 70% yield.

DIPEA (5 eq.) and HATU (1.2 eq.) were added to a solution of compound **62** (428 mg, 1 mmol) and compound **63** (1.1 eq.) in DMF (2 mL). After 30 min at rt, the mixture was subject to prep-HPLC to aﬀord the intermediates in 80% yield. A solution of the intermediate in 1:1 TFA/DCM was stirred at rt for 30 min. The solvents were evaporated under reduced pressure to give the corresponding deprotected intermediate **64** (TFA salt) that was used in the following reactions without further puriﬁcation (65% yield).

DIPEA (5 eq.) and HATU (1.2 eq.) were added to a solution of compound **2** (61.5 mg, 0.1 mmol) and compound **64** (1.1 eq.) in DMF (2 mL). After 30 min at rt, the mixture was subject to prep-HPLC to aﬀord compound **20**.

***N*-(5-(((5-(*tert*-butyl)oxazol-2-yl)methyl)thio)thiazol-2-yl)-1-(2-(4-(2-(4-((2*R*,3*S*,4*R*,5*S*)-3-(3-chloro-2-fluorophenyl)-4-(4-chloro-2-fluorophenyl)-4-cyano-5-neopentylpyrrolidine-2-carboxamido)-3-methoxybenzamido)ethyl)piperazin-1-yl)-2-oxoethyl)piperidine-4-carboxamide** **(20)**. White solid; Yield: 50%. ^1^H NMR (500 MHz, DMSO-*d*_6_) *δ* 12.33 (s, 1H), 10.41 (s, 1H), 8.54 (d, *J* = 5.0 Hz, 1H), 8.33 (d, *J* = 8.4 Hz, 1H), 7.75 (t, *J* = 6.6 Hz, 1H), 7.63 – 7.47 (m, 4H), 7.44 – 7.32 (m, 4H), 6.72 (s, 1H), 4.60 (d, *J* = 6.0 Hz, 2H), 4.38 (d, *J* = 8.7 Hz, 1H), 4.06 (s, 2H), 3.94 (s, 4H), 3.60 (p, *J* = 6.7 Hz, 2H), 3.55 – 3.41 (m, 6H), 3.12 (q, *J* = 7.3 Hz, 2H), 2.63 (d, *J* = 10.8 Hz, 3H), 1.93 – 1.71 (m, 4H), 1.28 (q, *J* = 6.6 Hz, 8H), 1.18 (s, 9H), 0.98 (s, 9H). ^13^C NMR (125 MHz, DMSO-*d*_6_) *δ* 170.45, 164.88, 162.41, 160.53, 160.23, 159.91, 158.12, 157.91, 155.96, 154.00, 146.92, 144.53, 134.21, 134.12, 130.39, 129.42, 129.05, 128.71, 128.03, 125.51, 125.40, 125.04, 124.71, 119.52, 119.48, 118.94, 118.79, 118.64, 118.56, 118.14, 117.08, 116.86, 116.71, 116.46, 109.07, 64.07, 62.86, 62.68, 62.63, 56.08, 55.28, 52.69, 52.07, 51.36, 49.56, 43.31, 40.96, 33.42, 30.33, 29.49, 28.91, 28.43, 27.98, 27.70. HRMS (ESI) calculated for C_56_H_67_Cl_2_F_2_N_10_O_6_S_2_ [M + H]^+^: 1147.4026, found: 1147.3995. UPLC-retention time: 4.847 min, purity >95%.

***General Procedure for Synthesis of Compound*** ***21****.*

K_2_CO_3_ (3 eq.) was added to a solution of the compound **1** (381 mg, 1 mmol) and compound **65** (1.2 eq.) in CH_3_CN was added. Then, the mixture was reﬂuxed overnight. After the solvents were evaporated under reduced pressure to aﬀord the corresponding crude that was subject to prep-HPLC to aﬀord the intermediates in 70% yield. The desired compound **66** was obtained by deprotection with TFA in DCM in 90% yield. ^1^H NMR (500 MHz, DMSO-*d*_6_) *δ* 8.30 (s, 1H), 7.38 (s, 1H), 6.72 (s, 1H), 4.05 (s, 2H), 2.85 (t, *J* = 14.0 Hz, 4H), 2.45 (d, *J* = 12.2 Hz, 2H), 2.12 (d, *J* = 6.9 Hz, 2H), 1.97 – 1.50 (m, 10H), 1.24 (s, 3H), 1.18 (s, 9H). HRMS (ESI) calculated for C_23_H_36_N_5_O_2_S_2_ [M + H]^+^: 478.2305, found: 478.2279.

K_2_CO_3_ (3 eq.) were added to a solution of the compound **66** (47.7 mg, 0.1 mmol) and compound **67** (1.2 eq.) in CH_3_CN was added. Then, the mixture was reﬂuxed overnight. After the solvents were evaporated under reduced pressure to aﬀord the corresponding crude that was subject to prep-HPLC to aﬀord the intermediates in 80 yields. A solution of the intermediate in 1:1 TFA/DCM was stirred at rt for 30 min. The solvents were evaporated under reduced pressure to give the corresponding deprotected intermediate **68** (TFA salt) that was used in the following reactions without further puriﬁcation (65% yield).

DIPEA (5 eq.) and HATU (1.2 eq.) were added to a solution of compound **2** (61.5 mg, 0.1 mmol) and compound **68** (1.1 eq.) in DMF (2 mL). After 30 min at rt, the mixture was subject to prep-HPLC to aﬀord compound **21**.

***N*-(5-(((5-(*tert*-butyl)oxazol-2-yl)methyl)thio)thiazol-2-yl)-1-((1-(2-(1-(4-((2*R*,3*S*,4*R*,5*S*)-3-(3-chloro-2-fluorophenyl)-4-(4-chloro-2-fluorophenyl)-4-cyano-5-neopentylpyrrolidine-2-carboxamido)-3-methoxybenzoyl)piperidin-4-yl)ethyl)piperidin-4-yl)methyl)piperidine-4-carboxamide (21).** White solid; Yield: 55%. ^1^H NMR (500 MHz, DMSO-*d*_6_) *δ* 12.26 (s, 1H), 10.37 (s, 1H), 8.31 (d, *J* = 8.2 Hz, 1H), 8.17 (s, 1H), 7.75 (t, *J* = 6.6 Hz, 1H), 7.64 – 7.50 (m, 2H), 7.43 – 7.32 (m, 4H), 7.10 (d, *J* = 1.7 Hz, 1H), 6.96 (dd, *J* = 8.2, 1.7 Hz, 1H), 6.72 (s, 1H), 4.60 (d, *J* = 10.7 Hz, 2H), 4.40 (t, *J* = 10.1 Hz, 1H), 4.05 (s, 2H), 3.94 (t, *J* = 11.3 Hz, 1H), 3.89 (s, 3H), 3.32 (d, *J* = 9.3 Hz, 2H), 2.81 (d, *J* = 108.0 Hz, 8H), 2.48 (d, *J* = 14.2 Hz, 1H), 2.21 (d, *J* = 6.7 Hz, 2H), 2.03 – 1.95 (m, 2H), 1.90 – 1.53 (m, 13H), 1.34 – 1.23 (m, 4H), 1.19 (s, 9H), 1.11 (s, 2H), 0.98 (s, 9H). ^13^C NMR (125 MHz, DMSO-*d*_6_) *δ* 174.16, 171.41, 168.95, 163.77, 161.70, 161.29, 159.21, 158.95, 157.02, 155.05, 148.13, 145.60, 135.29, 135.20, 132.15, 131.51, 130.50, 129.11, 128.18, 126.63, 126.52, 126.14, 125.80, 120.59, 120.00, 119.86, 119.75, 119.70, 119.63, 119.06, 118.17, 117.87, 117.77, 110.19, 65.16, 63.75, 56.32, 53.21, 50.59, 44.37, 41.60, 34.47, 33.89, 31.39, 30.65, 30.55, 29.96, 29.56, 28.76, 28.35. HRMS (ESI) calculated for C_61_H_76_Cl_2_F_2_N_9_O_5_S_2_ [M + H]^+^: 1186.4751, found:1186.4711. UPLC-retention time: 3.569 min, purity >95%.

***General Procedure for Synthesis of Compound*** ***22****.*

K_2_CO_3_ (3 eq.) was added to a solution of the compound **1** (381 mg, 1 mmol) and compound **67** (1.2 eq.) in CH_3_CN was added. Then, the mixture was reﬂuxed overnight. The solvents were evaporated under reduced pressure to aﬀord the corresponding crude compound that was subject to prep-HPLC to aﬀord the intermediates in 80% yields. A solution of the intermediate in 1:1 TFA/DCM was stirred at rt for 30 min. The solvents were evaporated under reduced pressure to give the corresponding deprotected compound **69** (TFA salt) that was used in the following reactions without further puriﬁcation (55% yield).

K_2_CO_3_ (3 eq.) was added to a solution of the compound **69** (49.1 mg, 0.1 mmol) and compound **70** (1.2 eq.) in CH_3_CN was added. Then, the mixture was reﬂuxed overnight. The solvents were evaporated under reduced pressure to aﬀord the corresponding crude that was subject to prep-HPLC to aﬀord the intermediates in 80 yields. A solution of the intermediate in 1:1 TFA/DCM was stirred at rt for 30 min. The solvents were evaporated under reduced pressure to give the corresponding deprotected intermediate **71** (TFA salt) that was used in the following reactions without further puriﬁcation (65% yield).

DIPEA (5 eq.) and HATU (1.2 eq.) were added to a solution of compound **2** (61.5 mg, 0.1 mmol) and compound **71** (1.1 eq.) in DMF (2 mL). After 30 min at rt, the mixture was subject to prep-HPLC to aﬀord compound **22**.

***N*-(5-(((5-(*tert*-butyl)oxazol-2-yl)methyl)thio)thiazol-2-yl)-1-(2-(1-(2-(4-((2*R*,3*S*,4*R*,5*S*)-3-(3-chloro-2-fluorophenyl)-4-(4-chloro-2-fluorophenyl)-4-cyano-5-neopentylpyrrolidine-2-carboxamido)-3-methoxybenzamido)ethyl)piperidin-4-yl)ethyl)piperidine-4-carboxamide (22).** White solid; Yield: 60%. ^1^H NMR (500 MHz, DMSO-*d*_6_) *δ* 12.30 (s, 1H), 10.42 (s, 1H), 8.55 (s, 1H), 8.33 (d, *J* = 8.4 Hz, 1H), 7.78 – 7.69 (m, 1H), 7.62 – 7.44 (m, 4H), 7.43 – 7.30 (m, 4H), 6.72 (s, 1H), 4.60 (d, *J* = 6.1 Hz, 2H), 4.39 (s, 1H), 4.05 (s, 2H), 3.93 (s, 4H), 3.47 (d, *J* = 5.6 Hz, 2H), 3.14 – 2.92 (m, 4H), 2.69 (s, 2H), 2.50 – 2.35 (m, 2H), 2.25 (t, *J* = 10.6 Hz, 2H), 2.14 (t, *J* = 10.8 Hz, 2H), 1.82 (d, *J* = 15.1 Hz, 2H), 1.77 – 1.62 (m, 5H), 1.43 (d, *J* = 5.8 Hz, 2H), 1.38 – 1.23 (m, 5H), 1.17 (s, 9H), 0.98 (s, 9H). ^13^C NMR (125 MHz, DMSO-*d*_6_) *δ* 173.90, 171.53, 165.99, 164.12, 161.67, 161.29, 159.21, 158.98, 155.06, 147.97, 145.60, 135.28, 135.19, 131.46, 130.50, 130.03, 129.80, 129.09, 126.56, 126.12, 125.78, 120.60, 120.56, 120.02, 119.86, 119.69, 119.61, 119.11, 118.15, 117.94, 117.79, 117.52, 110.11, 65.13, 63.90, 63.69, 57.18, 56.32, 55.49, 53.35, 52.44, 50.61, 44.36, 36.69, 34.47, 33.15, 32.39, 31.40, 31.33, 30.56, 29.97, 28.77, 28.73, 27.79. HRMS (ESI) calculated for C_57_H_70_Cl_2_F_2_N_9_O_5_S_2_ [M + H]^+^: 1132.4281, found: 1132.4246. UPLC-retention time: 3.545 min, purity >95%.

***General Procedure for Synthesis of Compound*** ***23****.*

A solution of compound **1** (381 mg, 1 mmol) and compound **72** (1 eq.) in 1:1 MeOH/DCM was stirred at rt for 30 min. Sodium cyanotrihydridoborate (2 eq.) was added and the mixture was stirred at rt for 4 h. After LC−MS demonstrated the full conversion of starting materials, H_2_O was added and the mixture was extracted with EtOAc three times. The combined organic layers were washed with brine, and then dried over anhydrous Na_2_SO_4_. The solvent was removed under reduced pressure. The desired compound **73** was obtained by deprotection with TFA in DCM in 60% yield.

K_2_CO_3_ (3 eq.) was added to a solution of the compound **73** (49.1 mg, 0.1 mmol) and compound **58** (1.2 eq.) in CH_3_CN was added. Then, the mixture was reﬂuxed overnight. The solvents were evaporated under reduced pressure to aﬀord the corresponding crude that was subject to prep-HPLC to aﬀord the intermediates in 80 yields. A solution of the intermediate in 1:1 TFA/DCM was stirred at rt for 30 min. The solvents were evaporated under reduced pressure to give the corresponding deprotected intermediate **74** (TFA salt) that was used in the following reactions without further puriﬁcation (55% yield).

DIPEA (5 eq.) and HATU (1.2 eq.) were added to a solution of compound **2** (52.2 mg, 0.1 mmol) and compound **74** (1.1 eq.) in DMF (2 mL). After 30 min at rt, the mixture was subject to prep-HPLC to aﬀord compound **23**.

***N*-(5-(((5-(*tert*-butyl)oxazol-2-yl)methyl)thio)thiazol-2-yl)-1'-(2-(2-(4-((2*R*,3*S*,4*R*,5*S*)-3-(3-chloro-2-fluorophenyl)-4-(4-chloro-2-fluorophenyl)-4-cyano-5-neopentylpyrrolidine-2-carboxamido)-3-methoxybenzamido)ethoxy)ethyl)-[1,4'-bipiperidine]-4-carboxamide (23)**. White solid; Yield: 60%.^1^H NMR (600 MHz, DMSO-*d*_6_) *δ* 12.45 (s, 1H), 10.42 (s, 1H), 8.61 (s, 1H), 8.35 (d, *J* = 8.4 Hz, 1H), 7.73 (t, *J* = 7.1 Hz, 1H), 7.62 – 7.50 (m, 4H), 7.43 – 7.30 (m, 4H), 6.72 (s, 1H), 4.60 (s, 2H), 4.39 (d, *J* = 12.5 Hz, 1H), 4.07 (s, 2H), 3.94 (s, 4H), 3.78 (s, 2H), 3.61 (t, *J* = 5.7 Hz, 3H), 3.52 – 3.44 (m, 4H), 3.26 (s, 2H), 2.96 (s, 2H), 2.76 (s, 1H), 2.28 – 1.83 (m, 9H), 1.64 (dd, *J* = 14.3, 9.9 Hz, 1H), 1.30 – 1.22 (m, 4H), 1.18 (s, 9H), 0.97 (s, 9H). ^13^C NMR (150 MHz, DMSO-*d*_6_) *δ* 170.49, 165.04, 160.44, 160.23, 159.72, 158.14, 158.05, 155.77, 154.13, 146.92, 144.55, 134.22, 134.14, 130.40, 129.42, 129.04, 128.90, 128.74, 127.97, 125.40, 125.31, 125.07, 124.69, 119.52, 118.91, 118.79, 118.53, 118.47, 118.38, 117.06, 116.88, 116.69, 116.46, 109.06, 68.47, 64.01, 62.91, 62.63, 62.59, 55.28, 54.61, 50.08, 49.47, 47.44, 43.27, 33.41, 30.32, 29.47, 28.87, 27.70. HRMS (ESI) calculated for C_57_H_70_Cl_2_F_2_N_9_O_6_S_2_ [M + H]^+^: 1148.4230, found: 1148.4209. UPLC-retention time: 6.993 min, purity >95%.

**Part 3. ^1^H and ^13^C NMR Spectra**


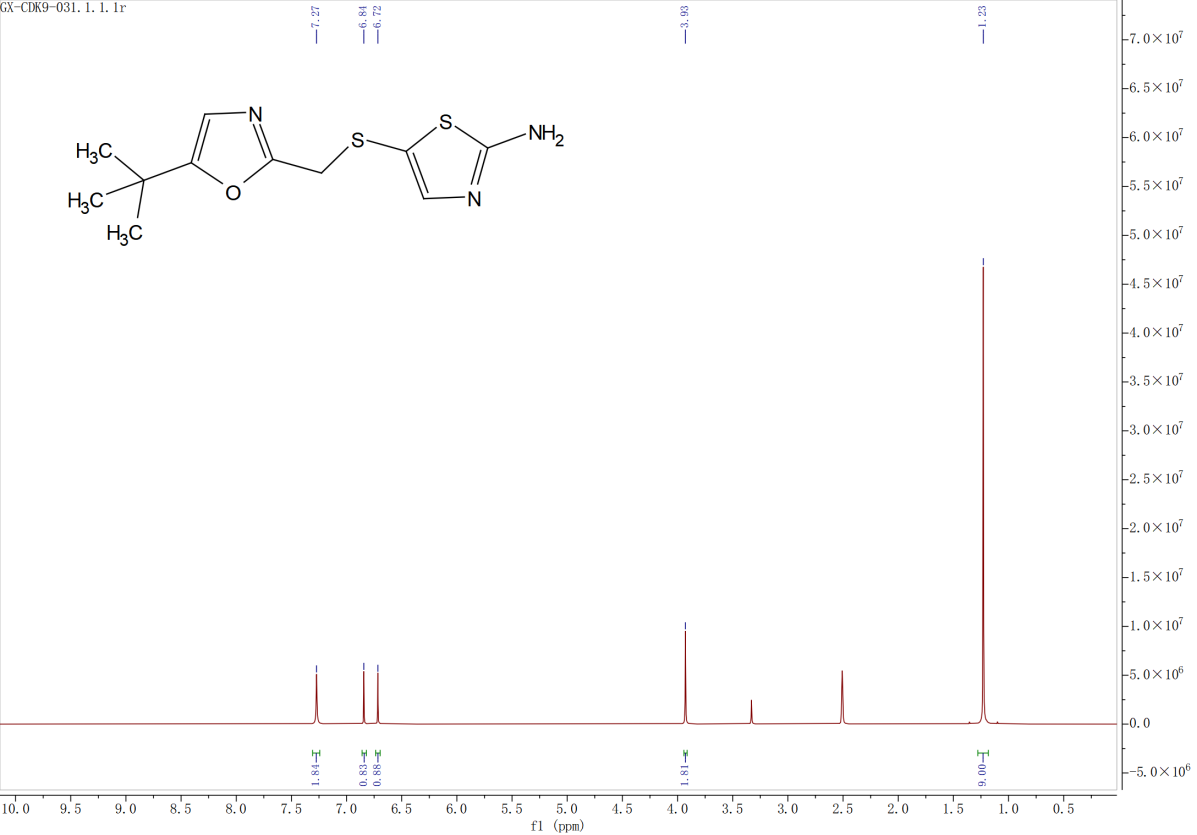


^1^H NMR Spectra of **INT 31** in DMSO-*d*_6_


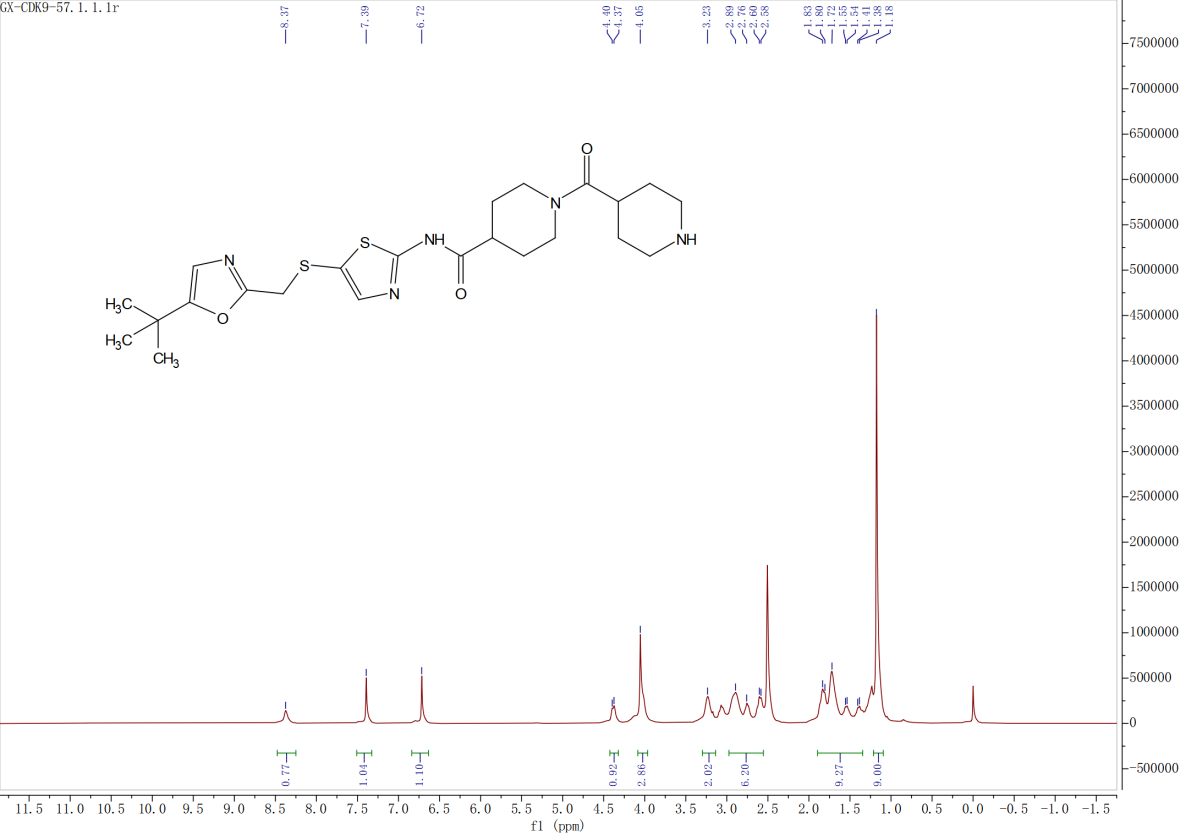


^1^H NMR Spectra of **INT 57** in DMSO-*d*_6_


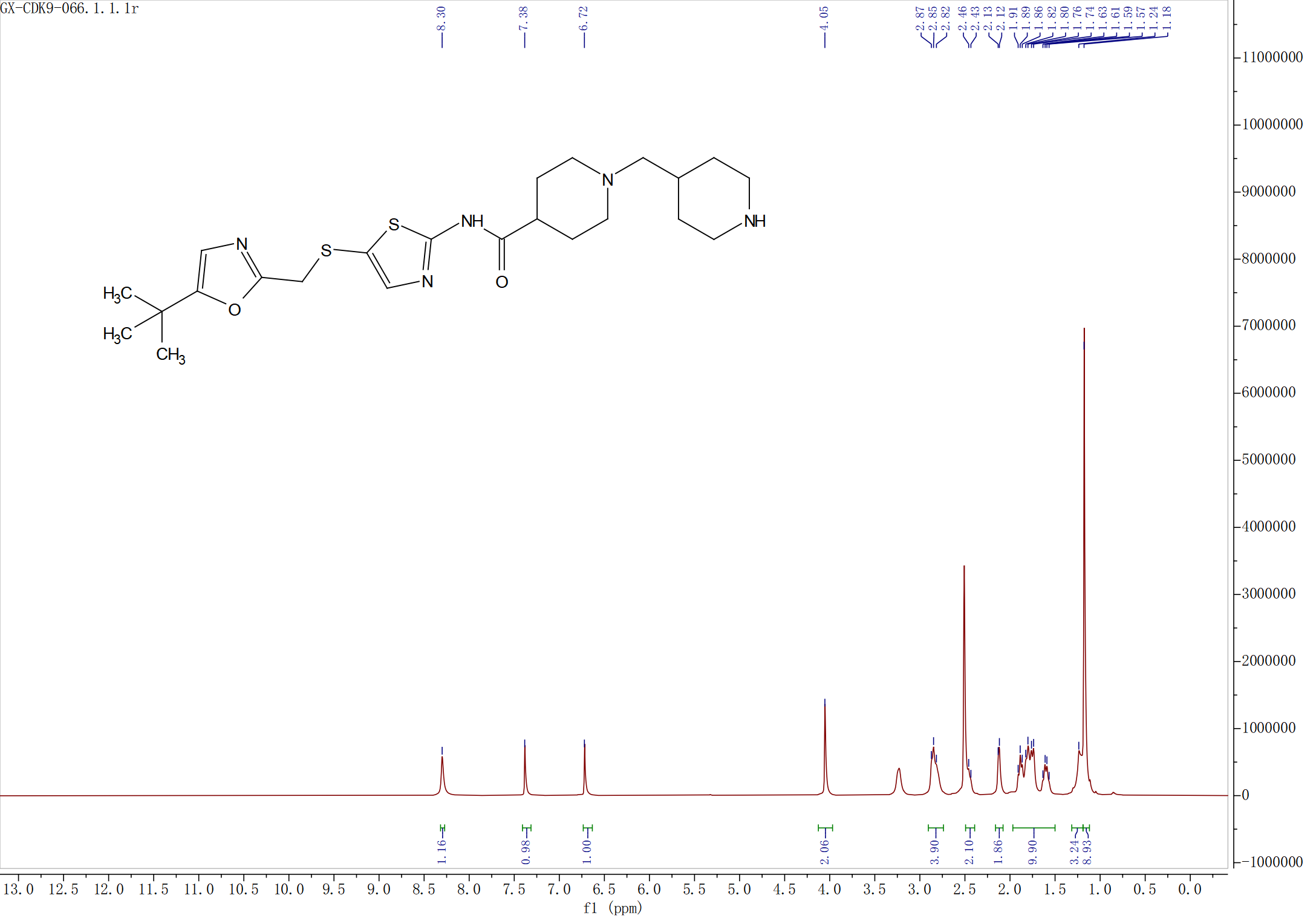


^1^H NMR Spectra of **INT 66** in DMSO-*d*_6_


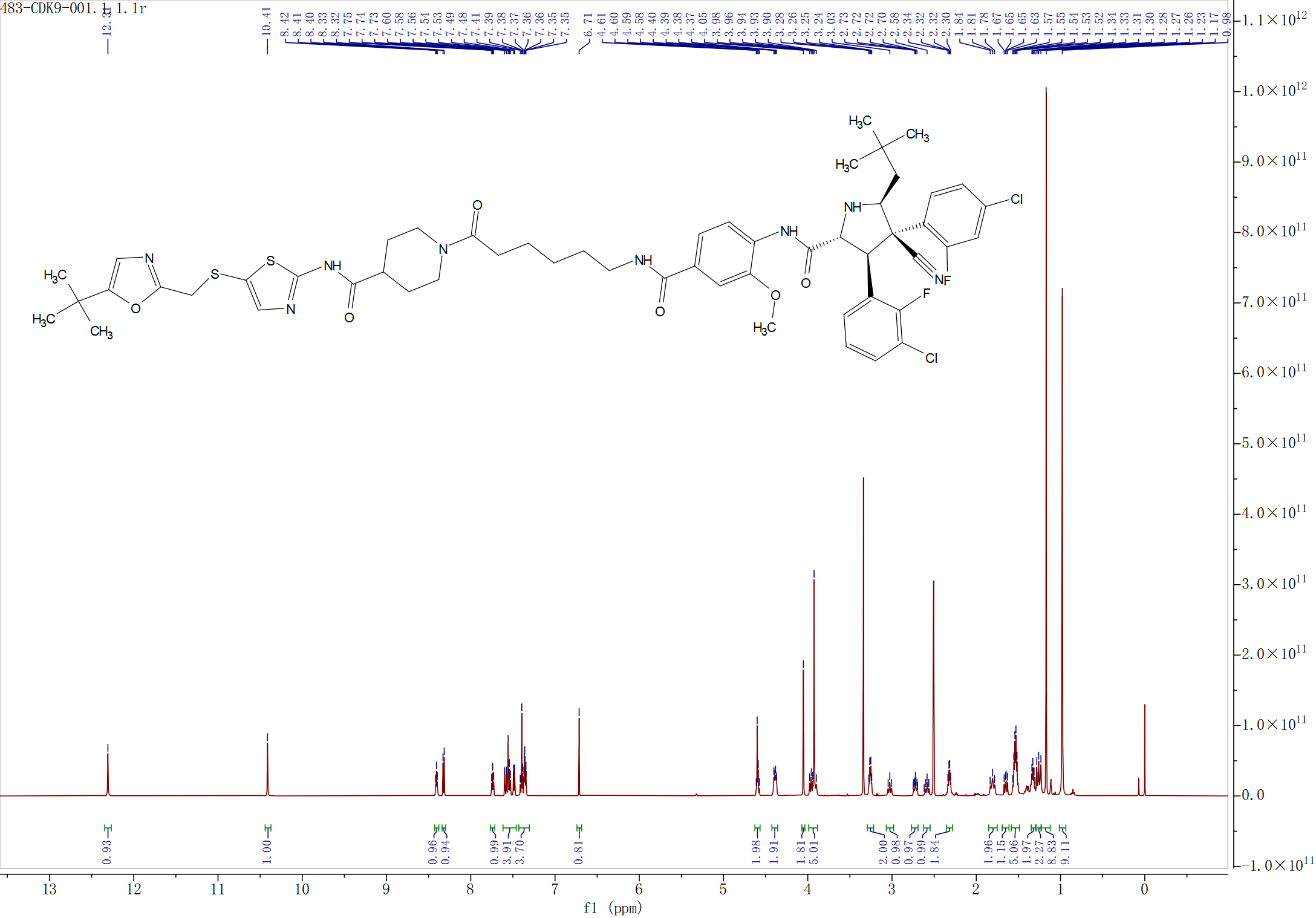


^1^H NMR Spectra of **4** (**dCDK9**-**001**) in DMSO-*d*_6_


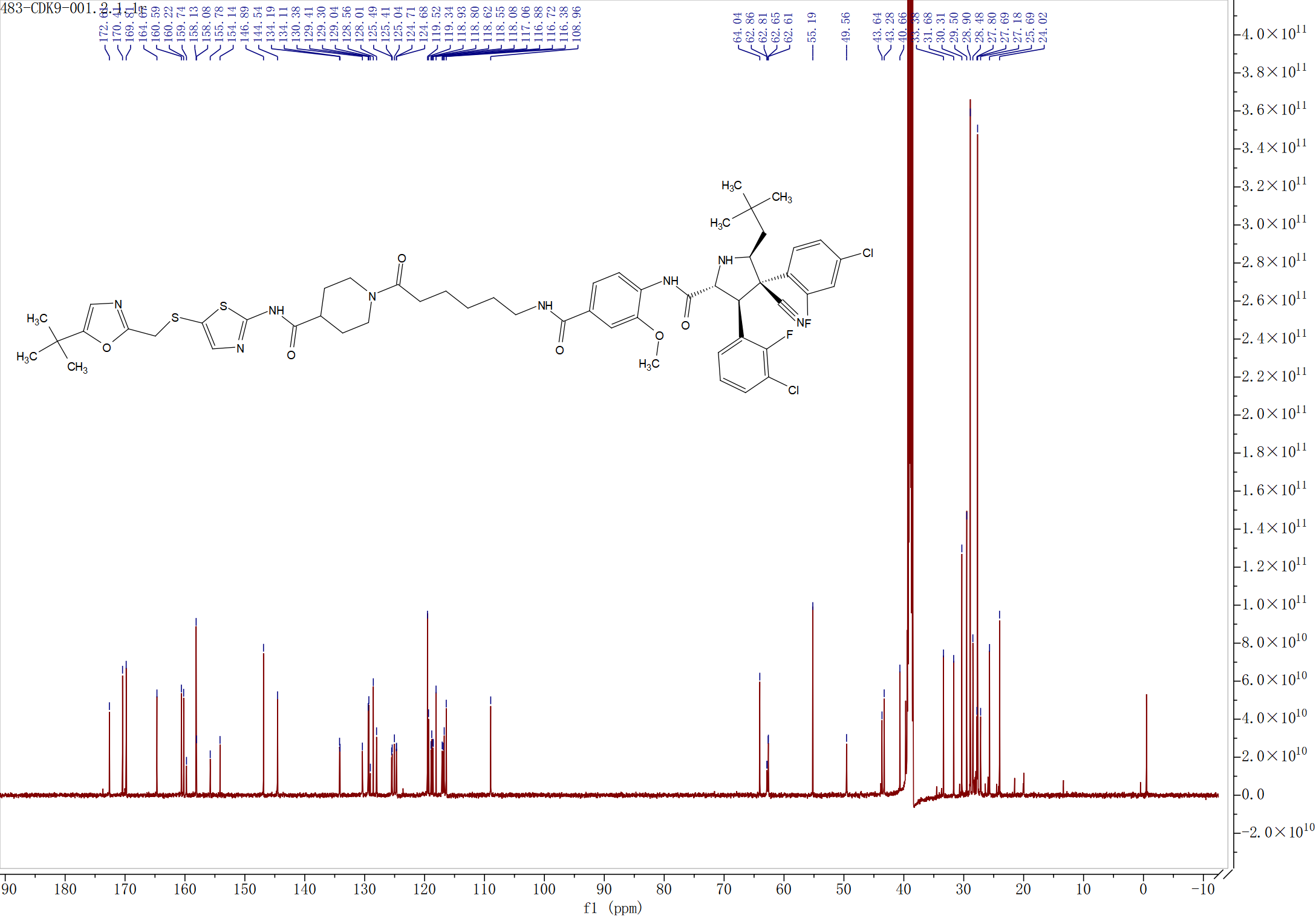


^13^C NMR Spectra of **4** (**dCDK9**-**001**) in DMSO-*d*_6_


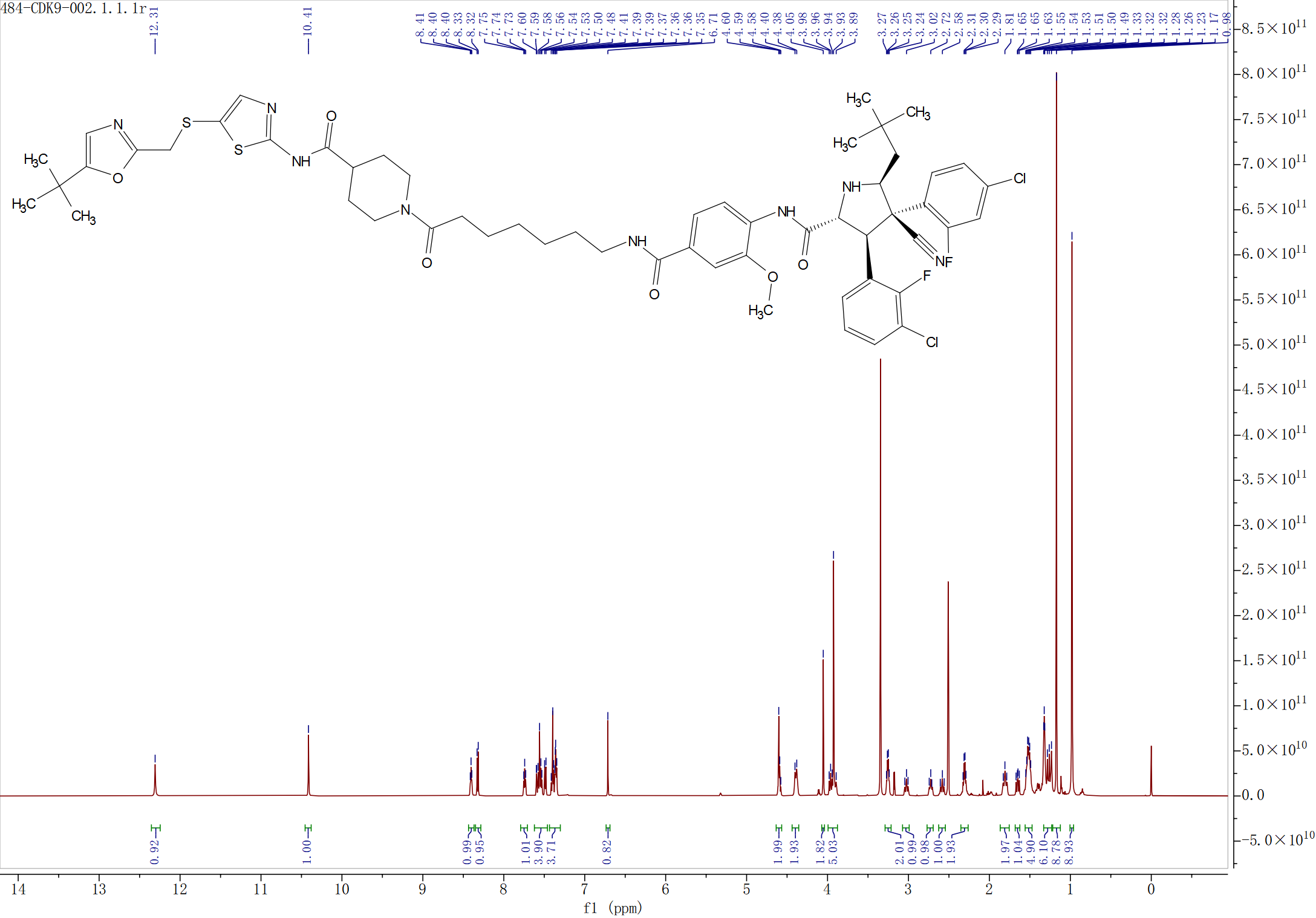


^1^H NMR Spectra of **5** (**dCDK9**-**002**) in DMSO-*d*_6_


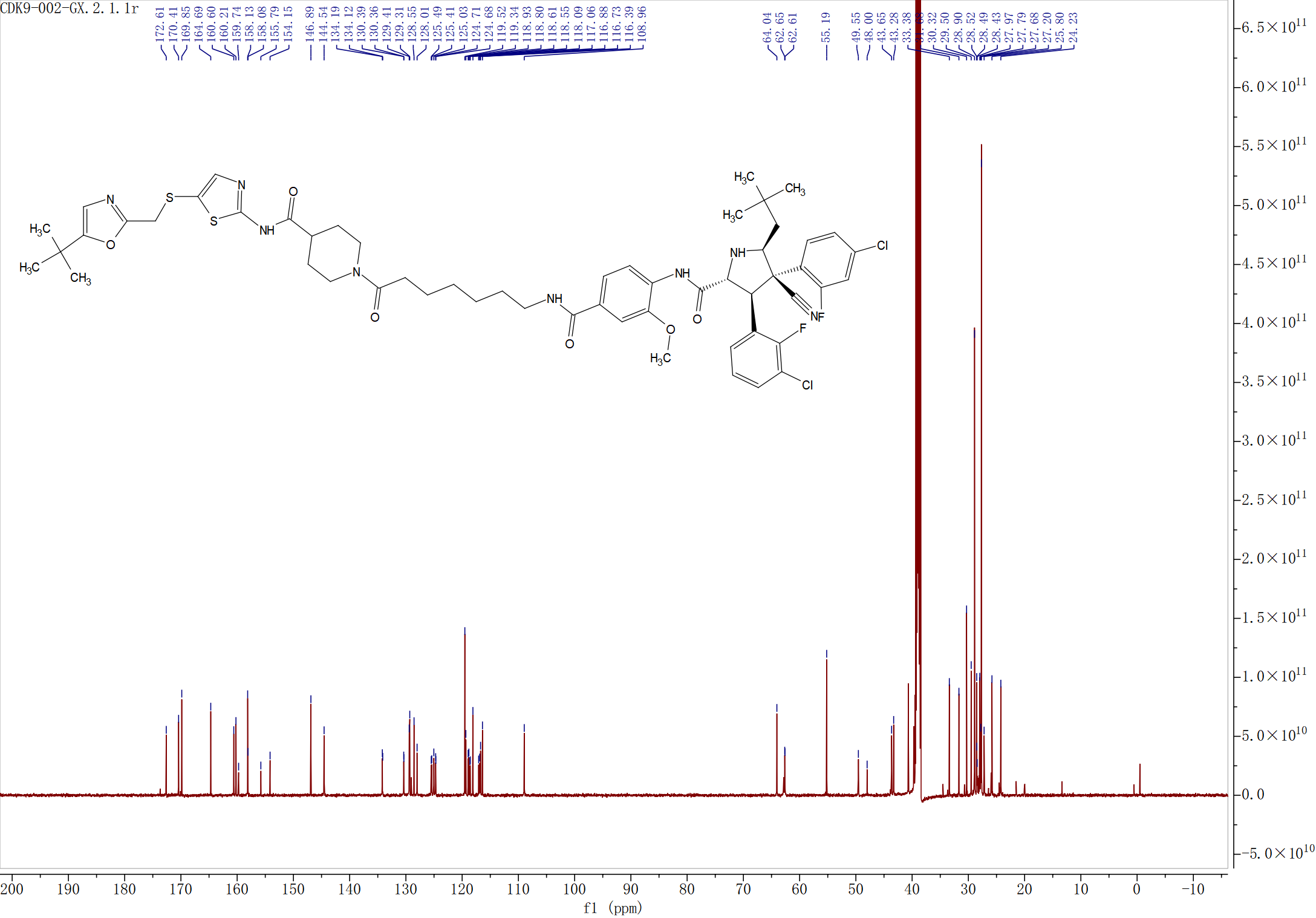


^13^C NMR Spectra of **5** (**dCDK9**-**002**) in DMSO-*d*_6_


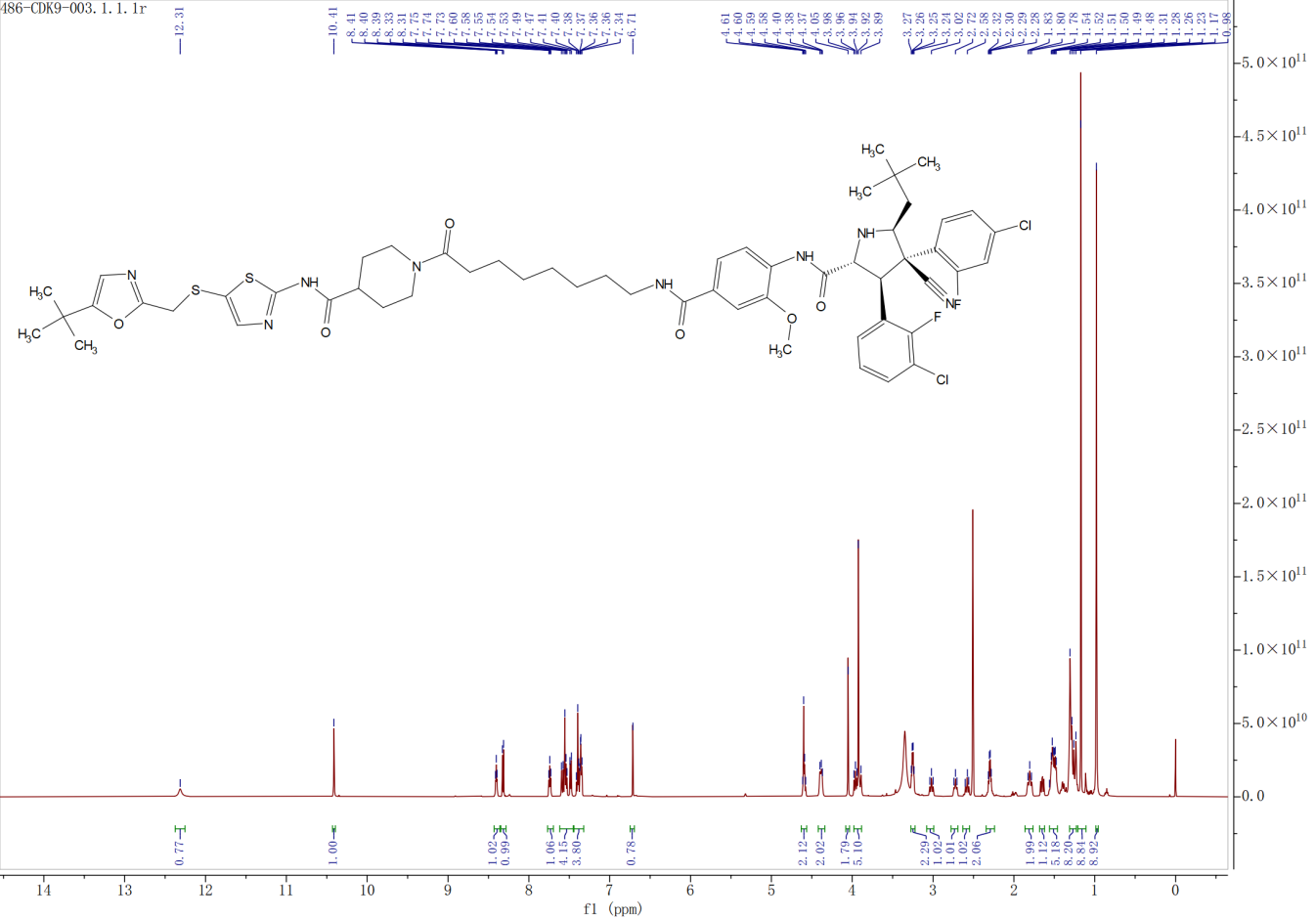


^1^H NMR Spectra of **6** (**dCDK9**-**003**) in DMSO-*d*_6_


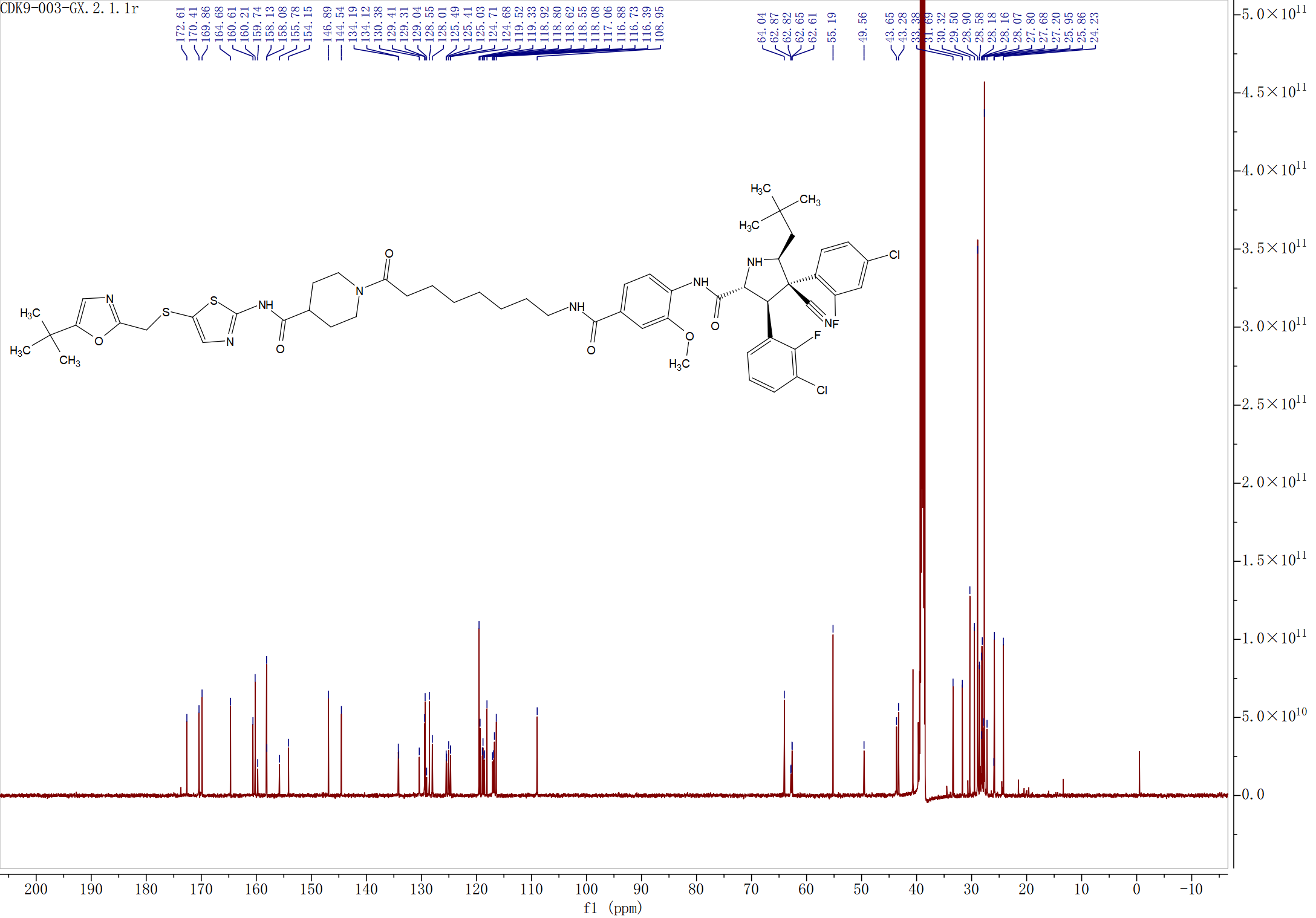


^13^C NMR Spectra of **6** (**dCDK9**-**003**) in DMSO-*d*_6_


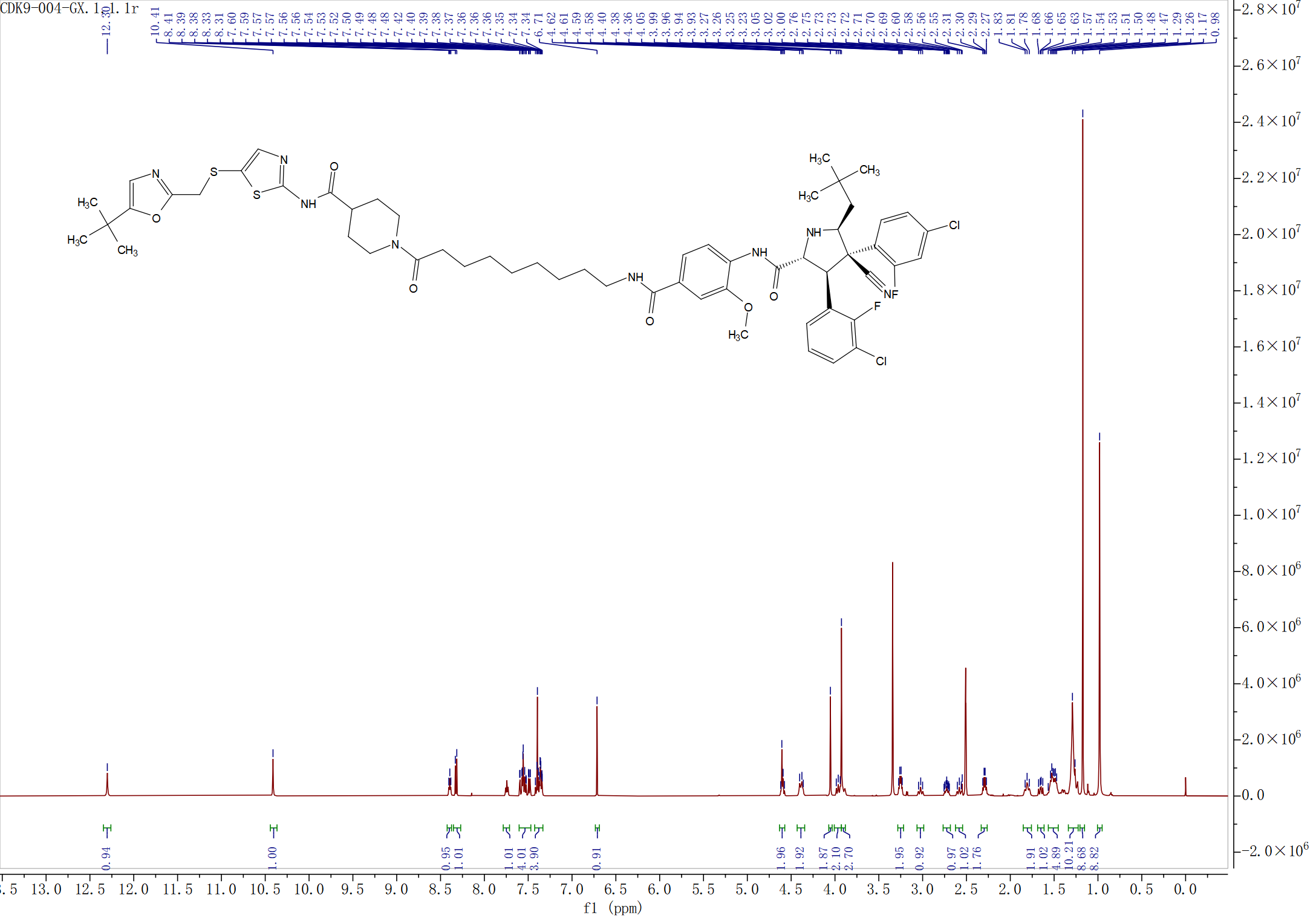


^1^H NMR Spectra of **7** (**dCDK9**-**004**) in DMSO-*d*_6_


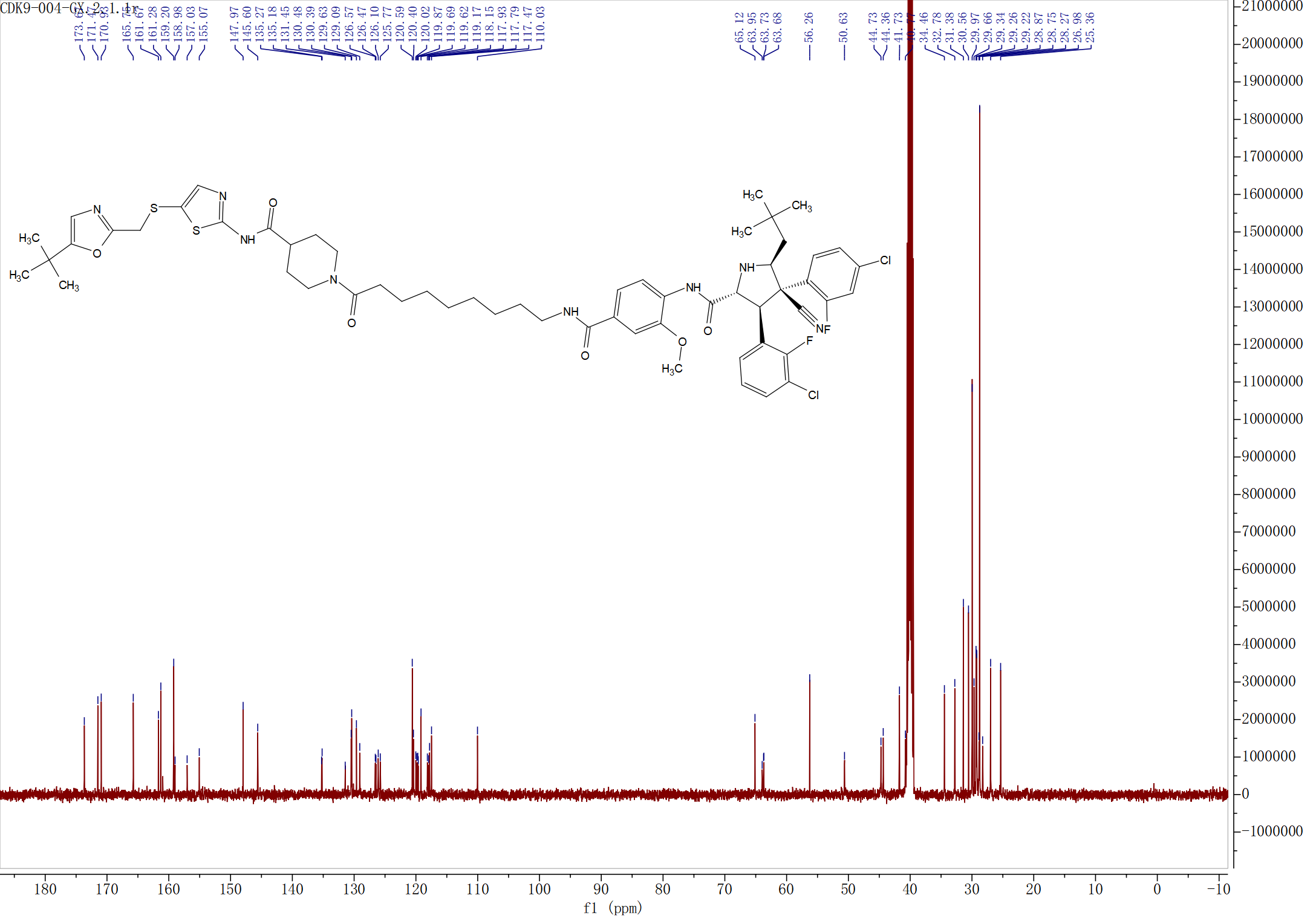


^13^C NMR Spectra of **7** (**dCDK9**-**004**) in DMSO-*d*_6_


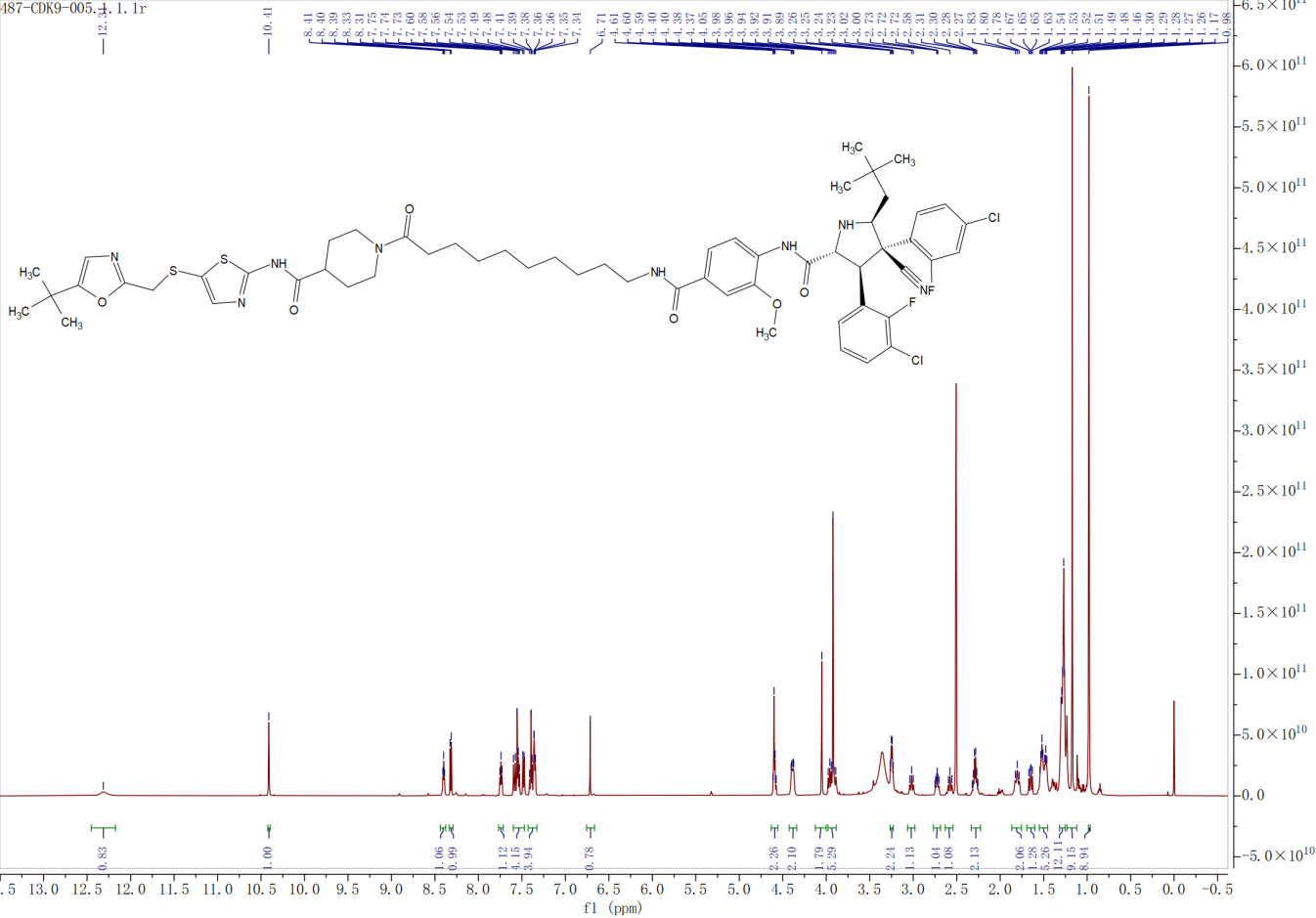


^1^H NMR Spectra of **8** (**dCDK9**-**005**) in DMSO-*d*_6_


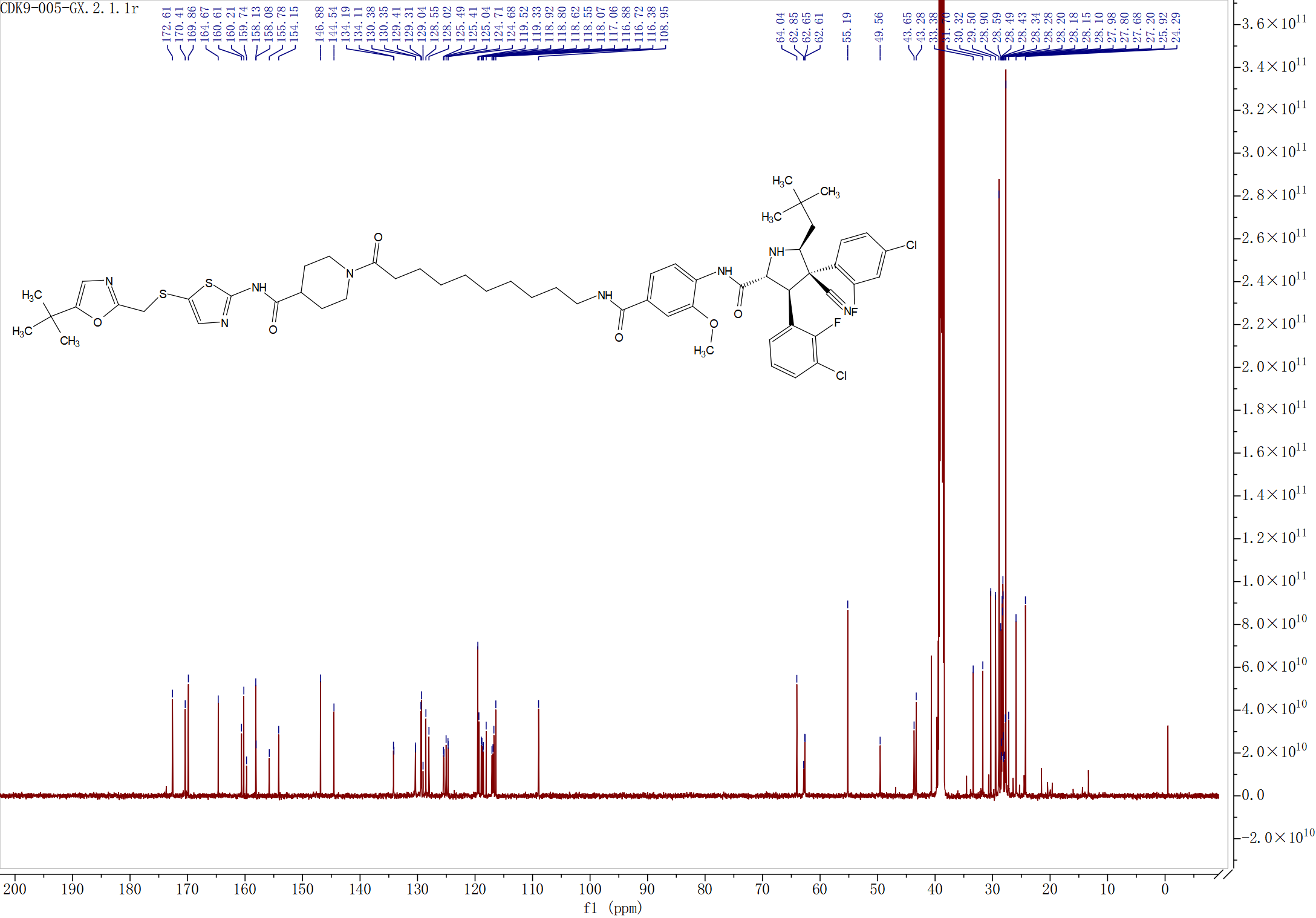


^13^C NMR Spectra of **8** (**dCDK9**-**005**) in DMSO-*d*_6_


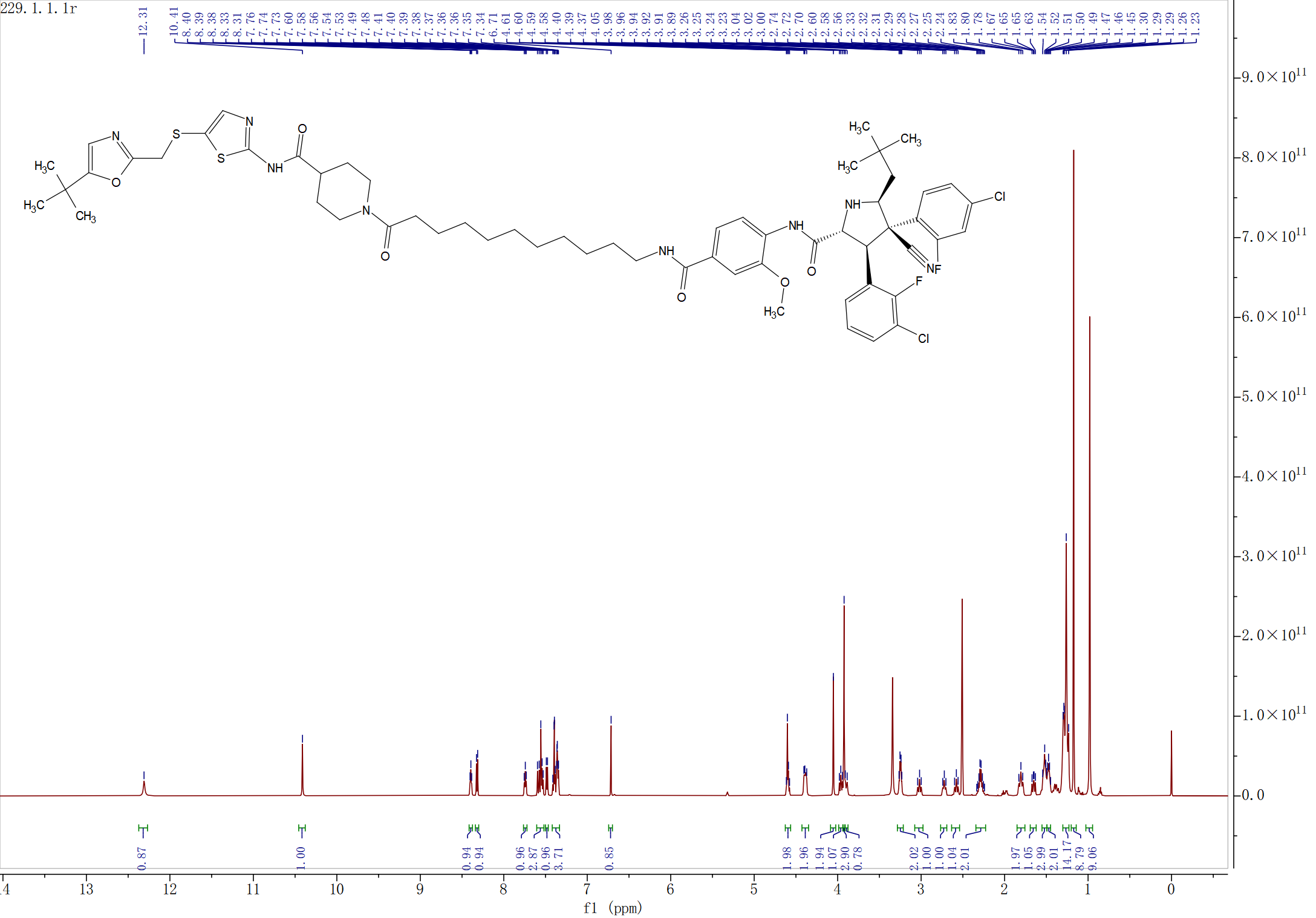


^1^H NMR Spectra of **9** (**dCDK9**-**006**) in DMSO-*d*_6_


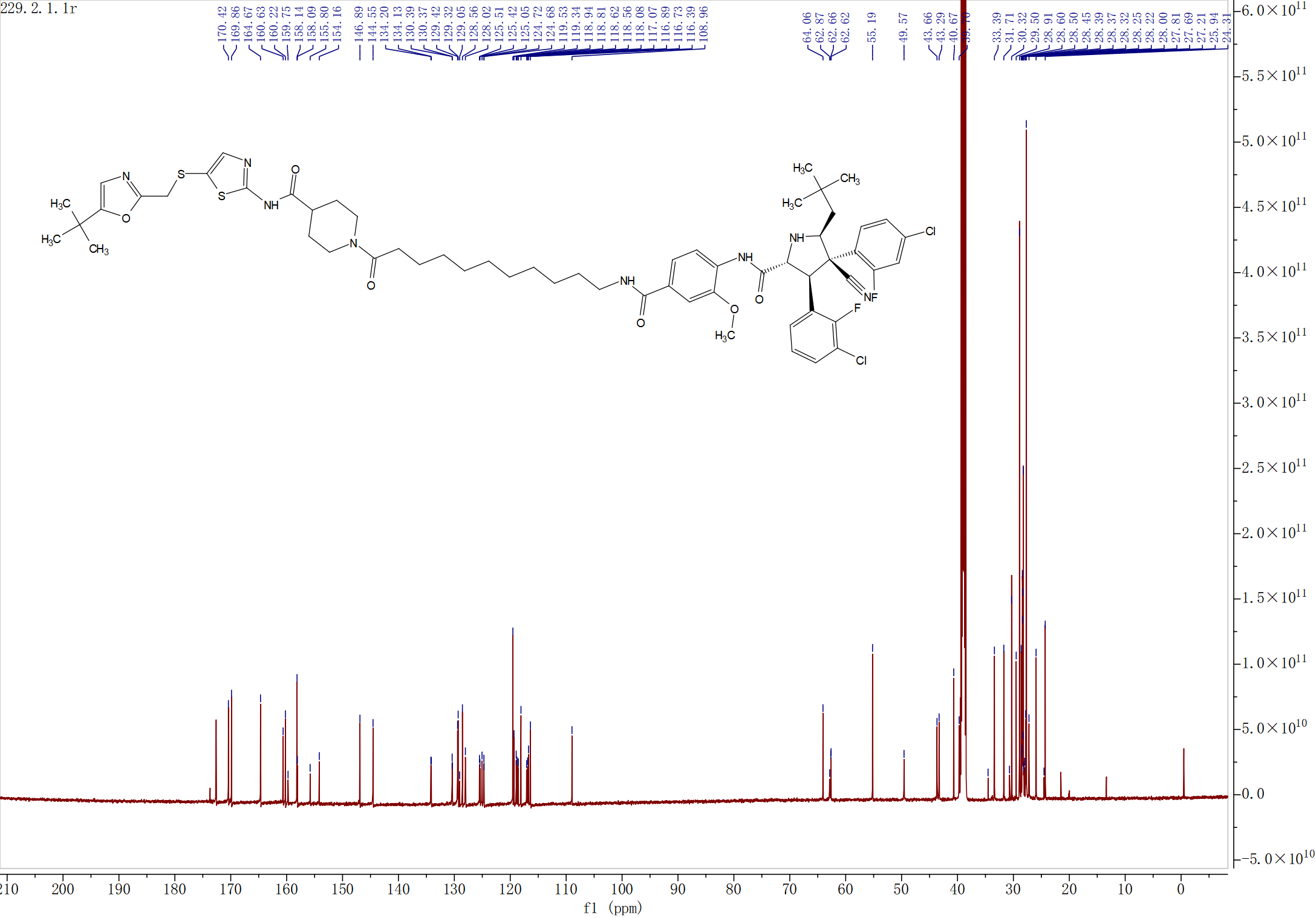


^13^C NMR Spectra of **9** (**dCDK9**-**006**) in DMSO-*d*_6_


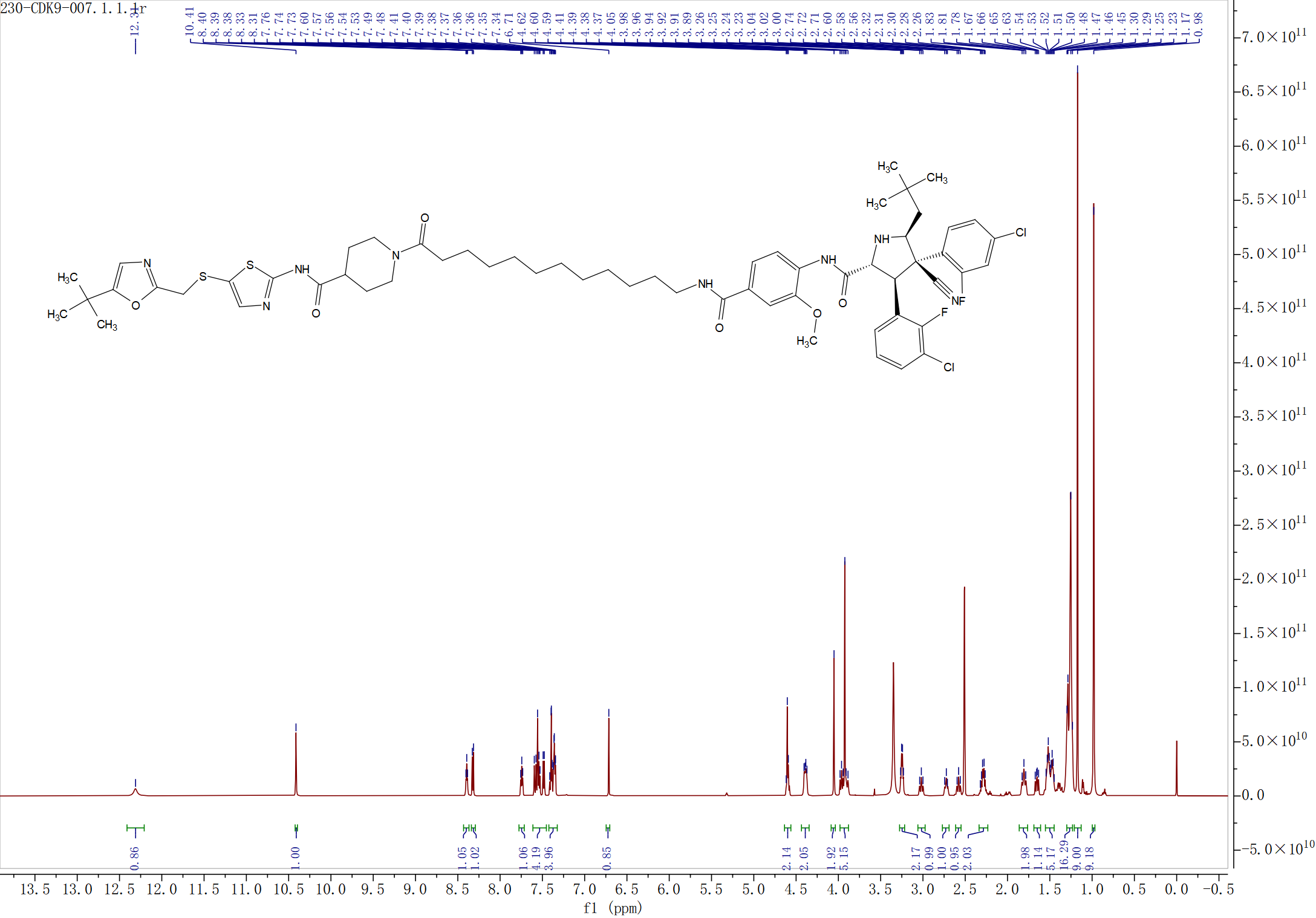


^1^H NMR Spectra of **10** (**dCDK9**-**007**) in DMSO-*d*_6_


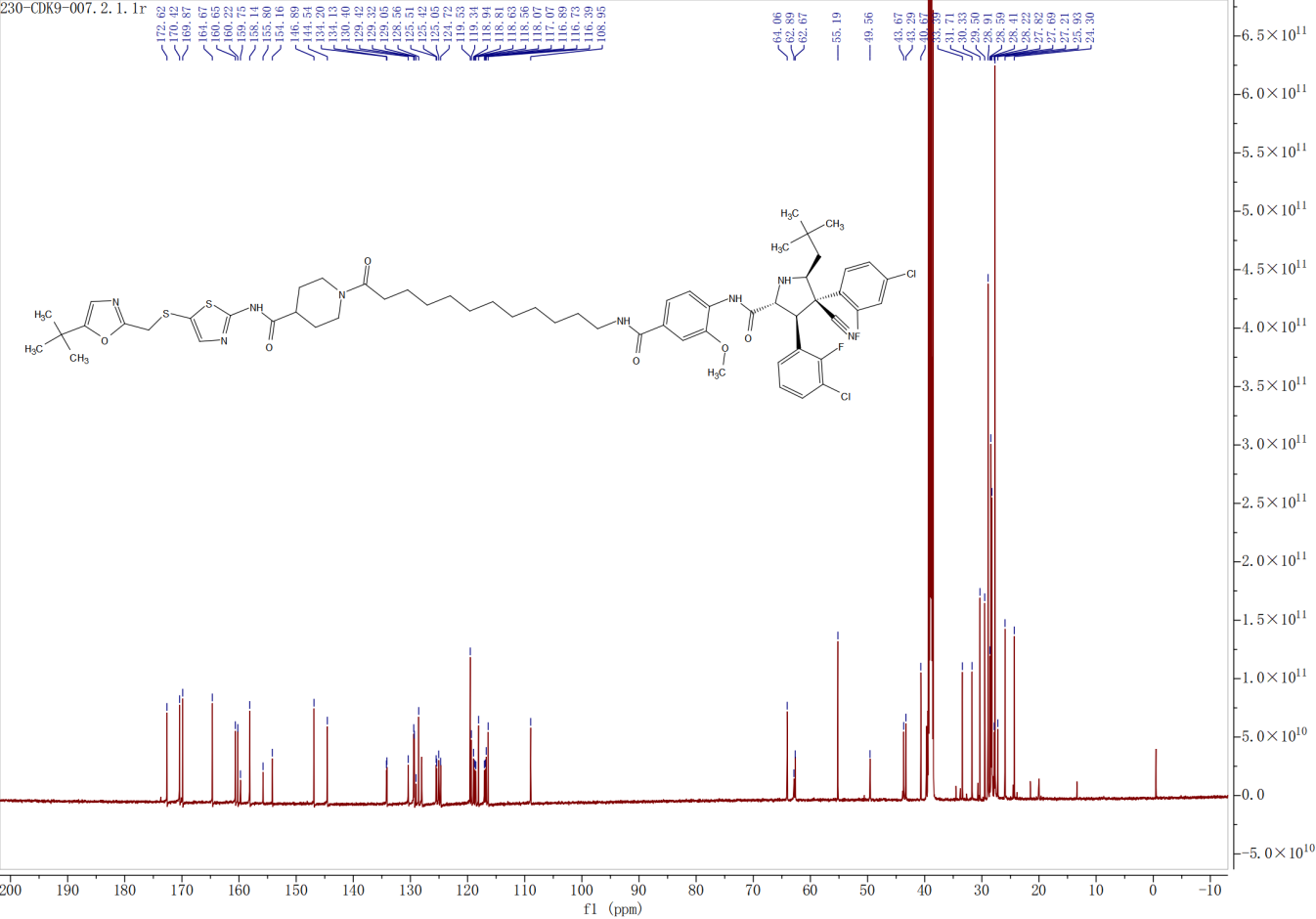


^13^C NMR Spectra of **10** (**dCDK9**-**007**) in DMSO-*d*_6_

**
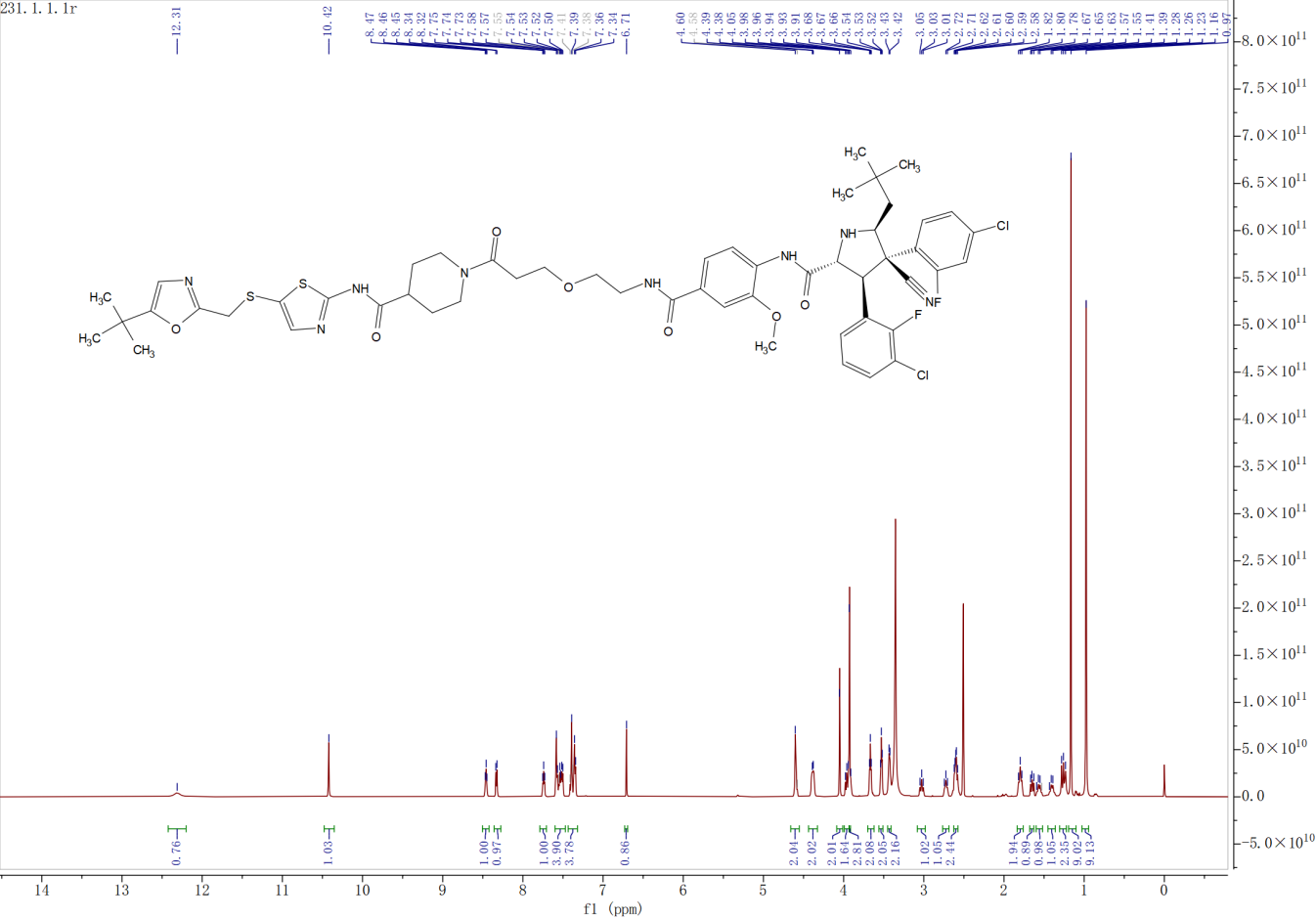
**

^1^H NMR Spectra of **11** (**dCDK9**-**008**) in DMSO-*d*_6_

**
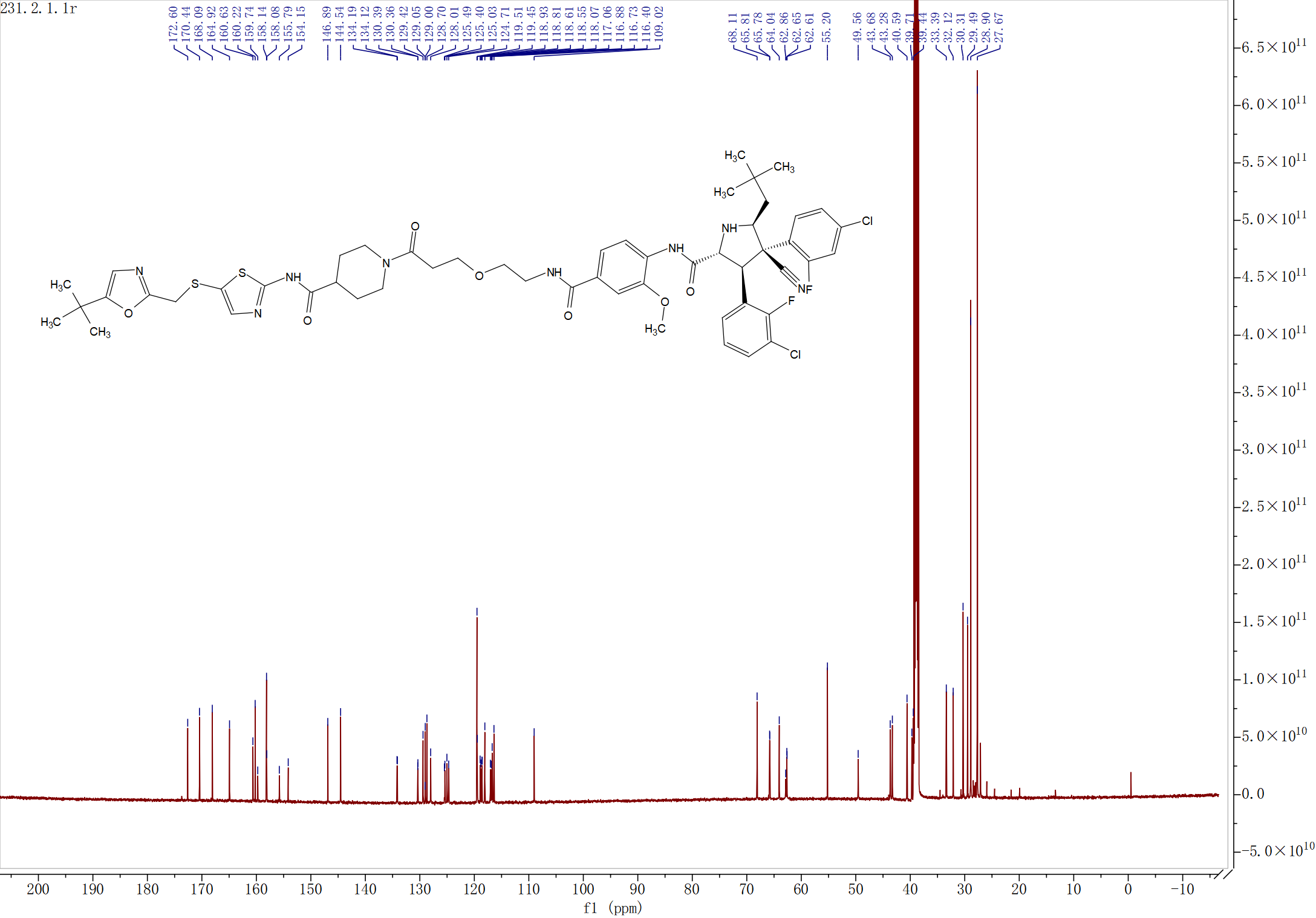
**

^13^C NMR Spectra of **11** (**dCDK9**-**008**) in DMSO-*d*_6_

**
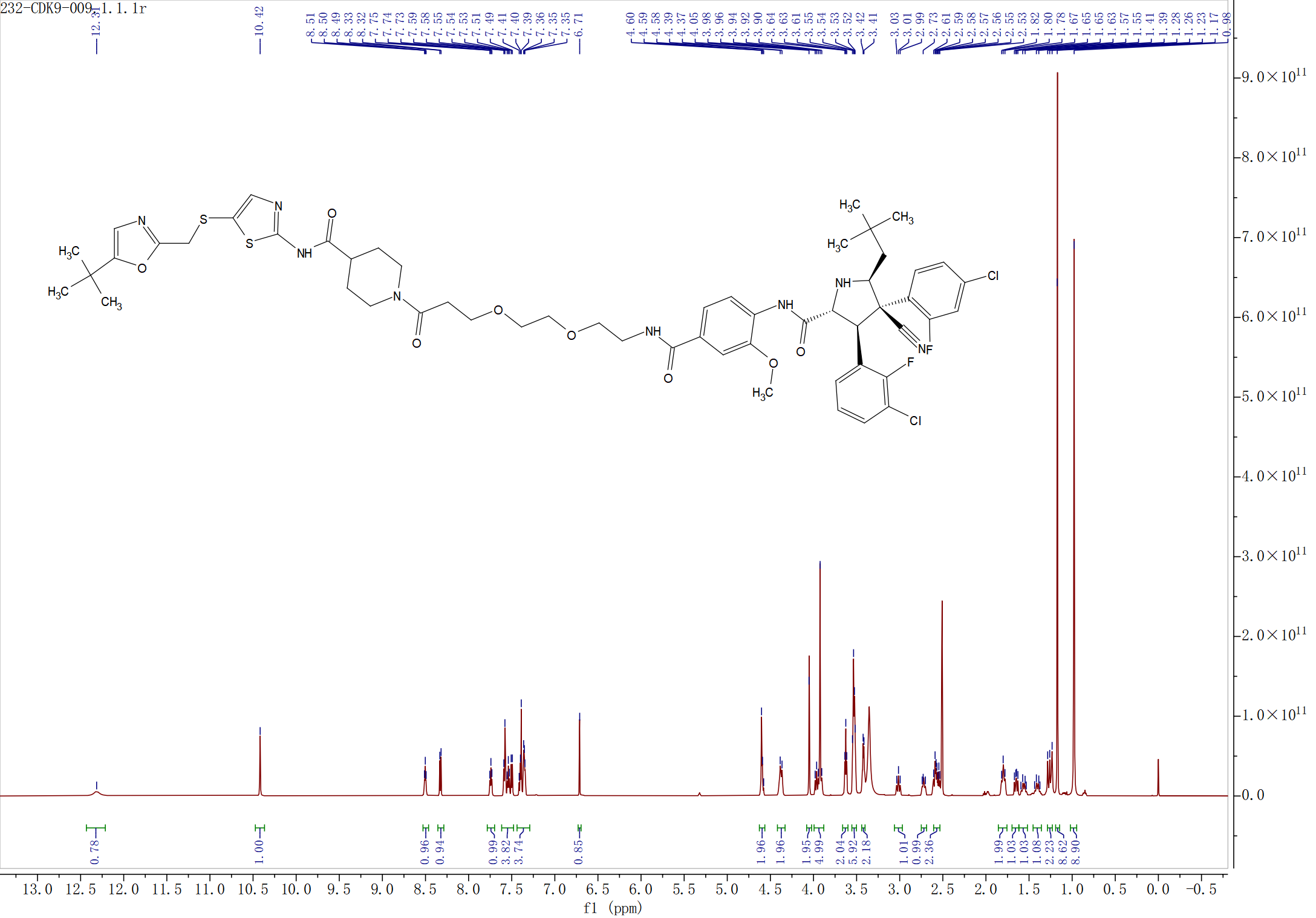
**

^1^H NMR Spectra of **12** (**dCDK9**-**009**) in DMSO-*d*_6_


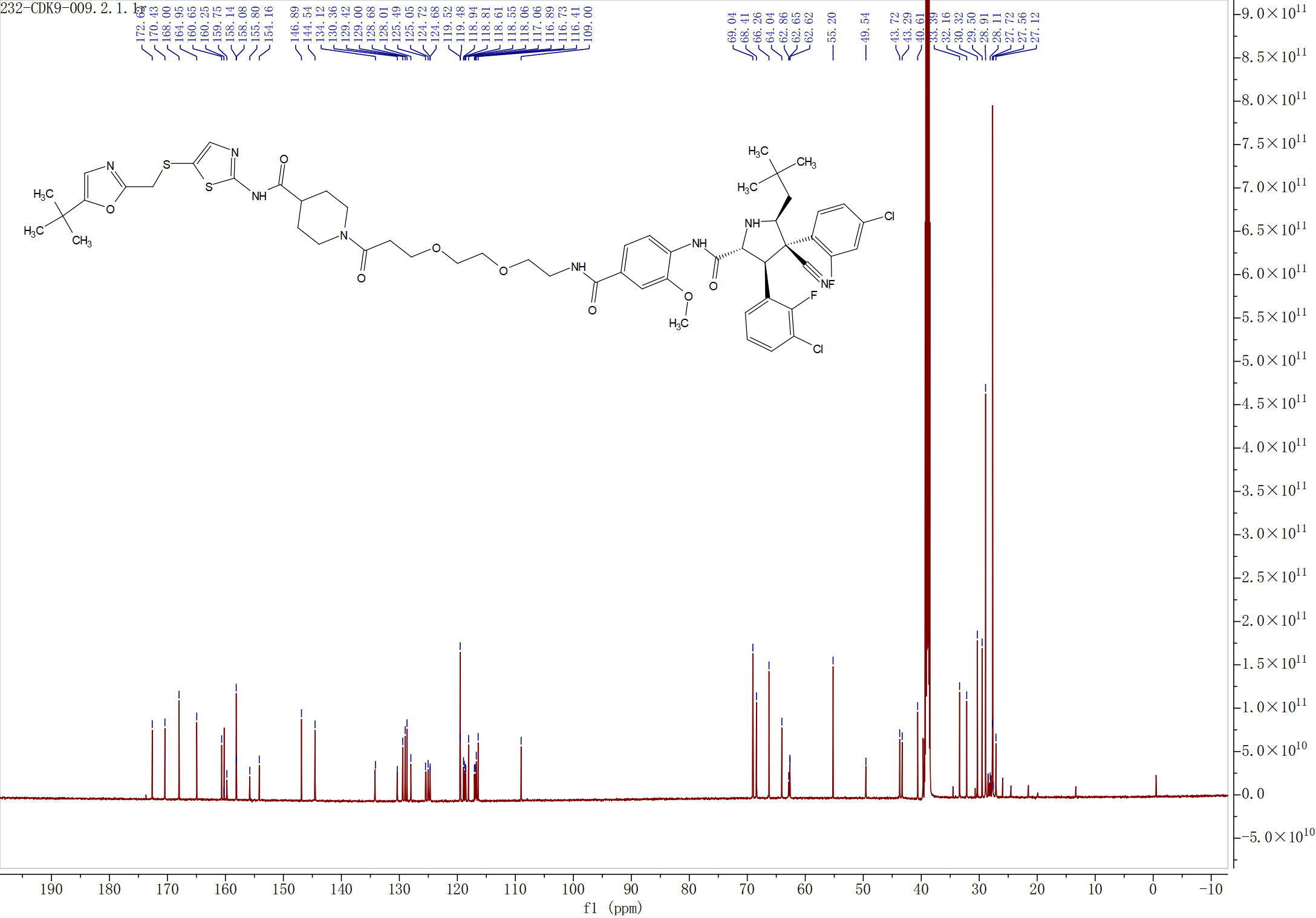


^13^C NMR Spectra of **12** (**dCDK9**-**009**) in DMSO-*d*_6_

**
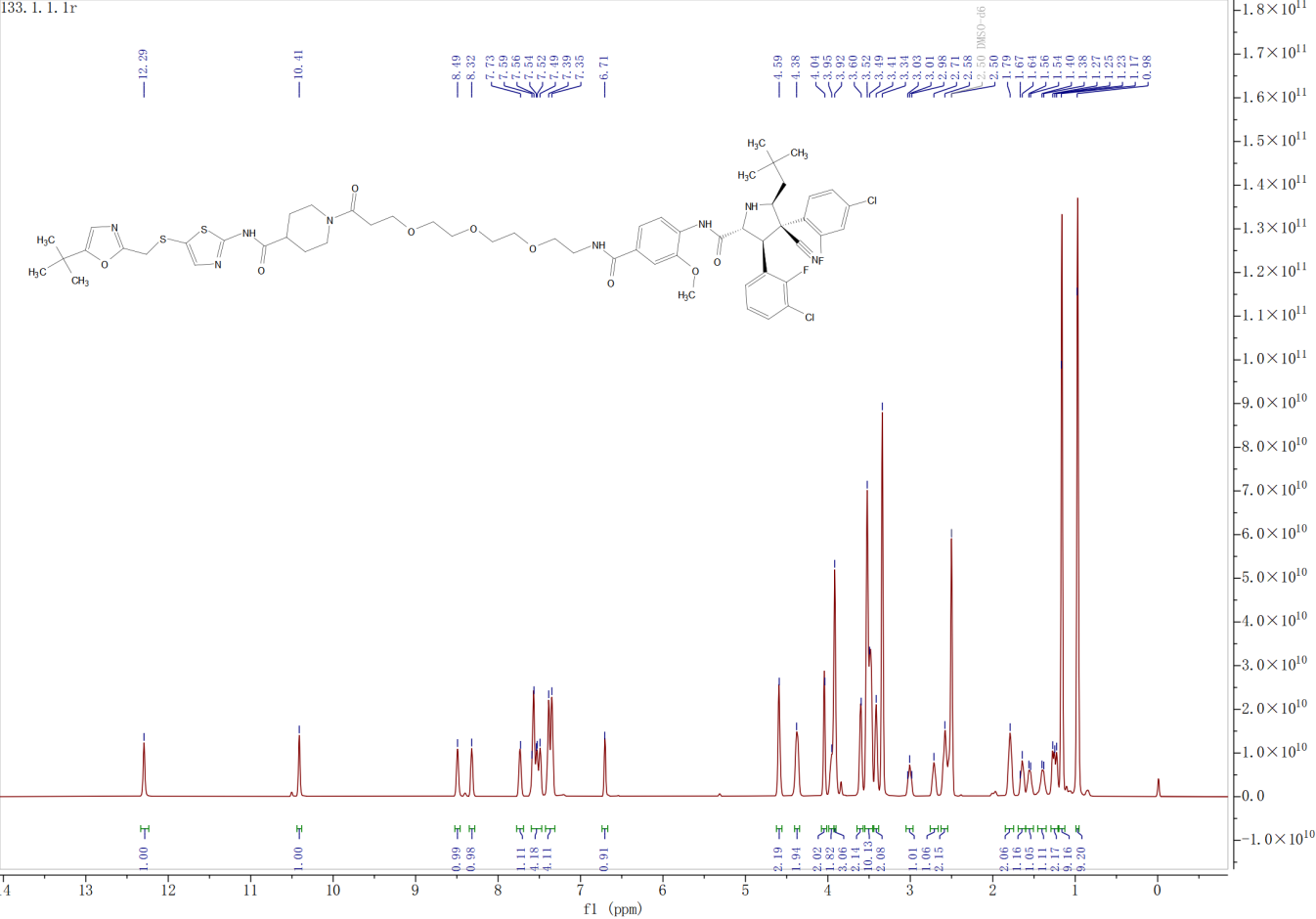
**

^1^H NMR Spectra of **13** (**dCDK9**-**010**) in DMSO-*d*_6_

**
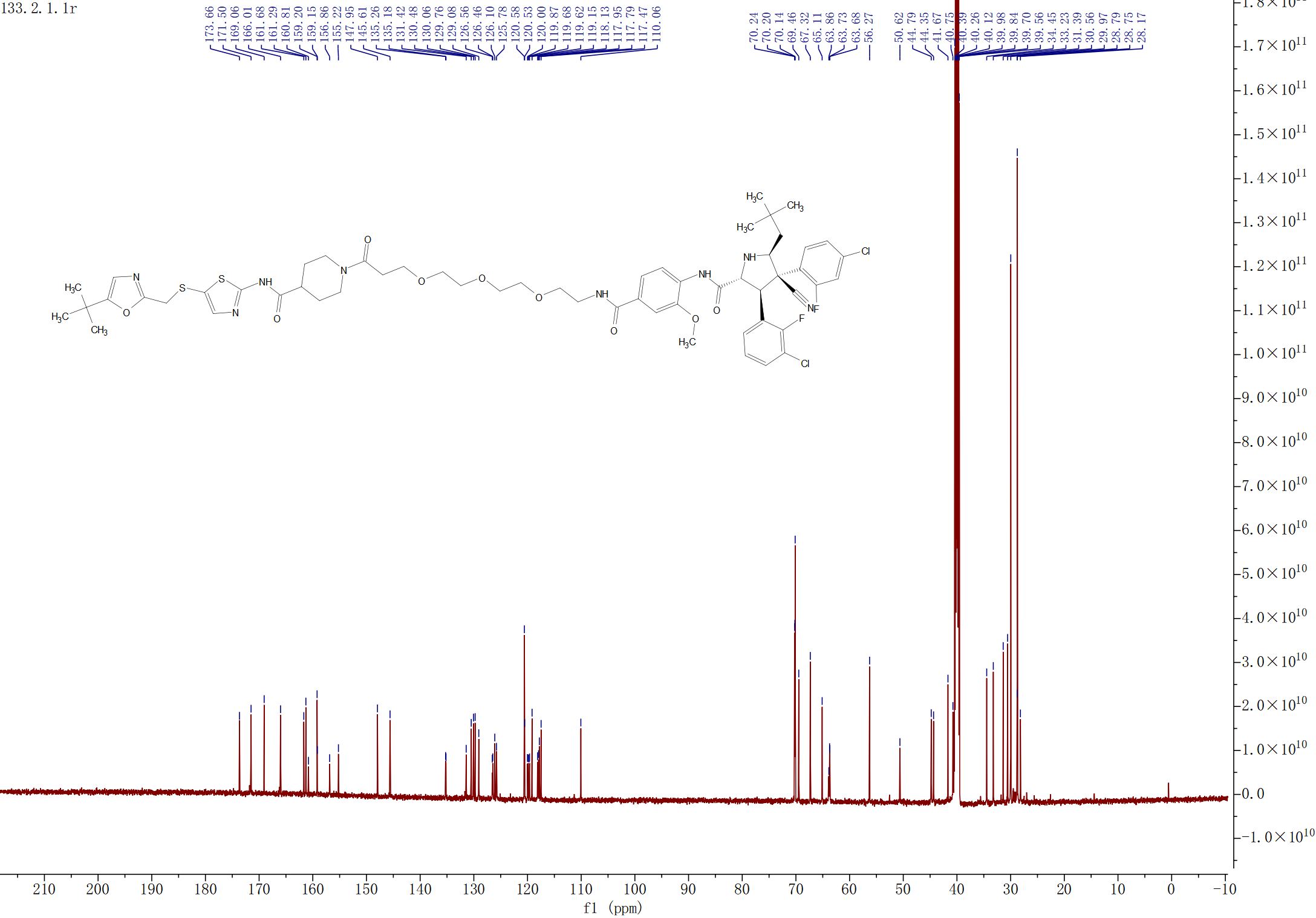
**

^13^C NMR Spectra of **13** (**dCDK9**-**010**) in DMSO-*d*_6_

**
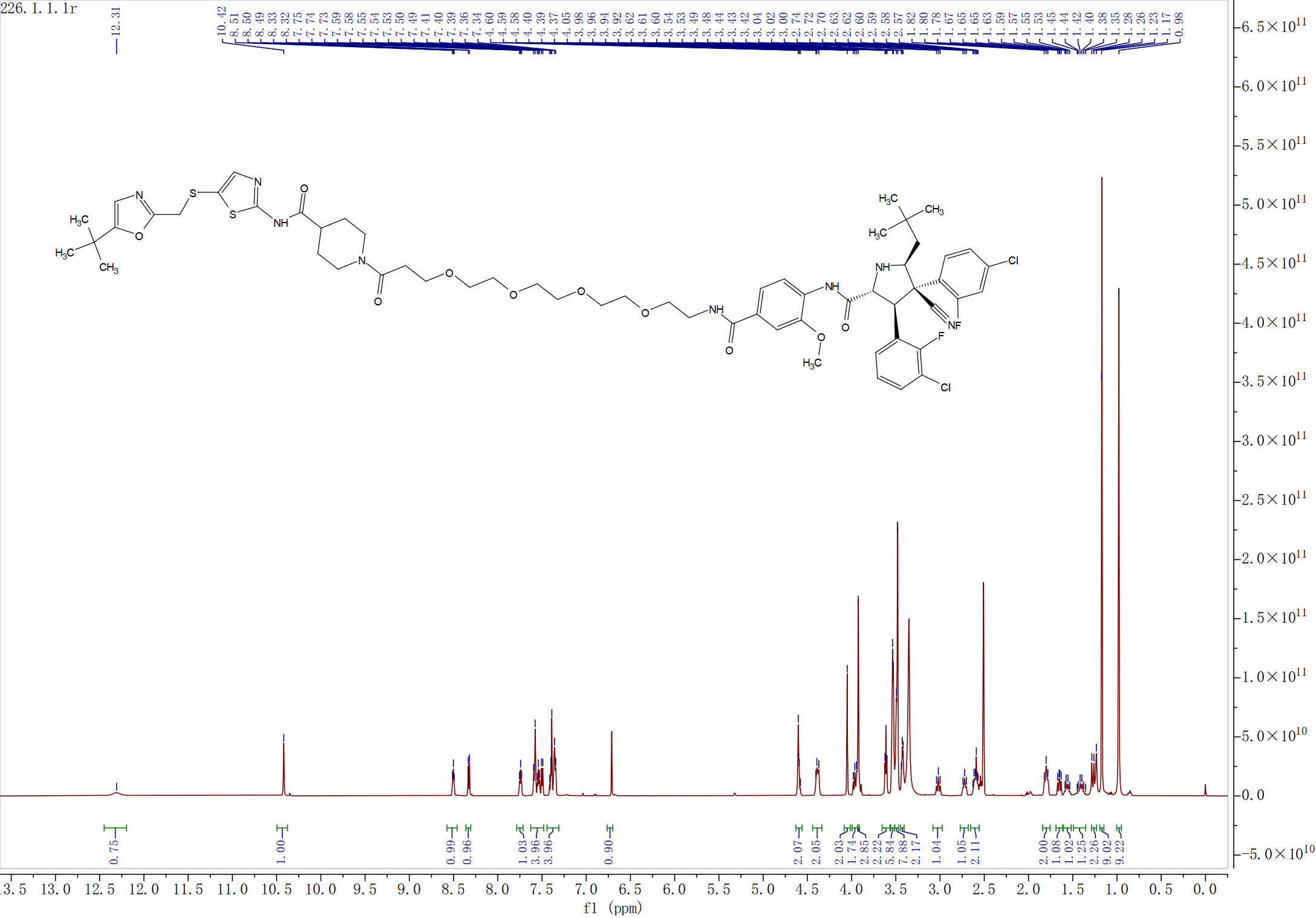
**

^1^H NMR Spectra of **14** (**dCDK9**-**012**) in DMSO-*d*_6_

**
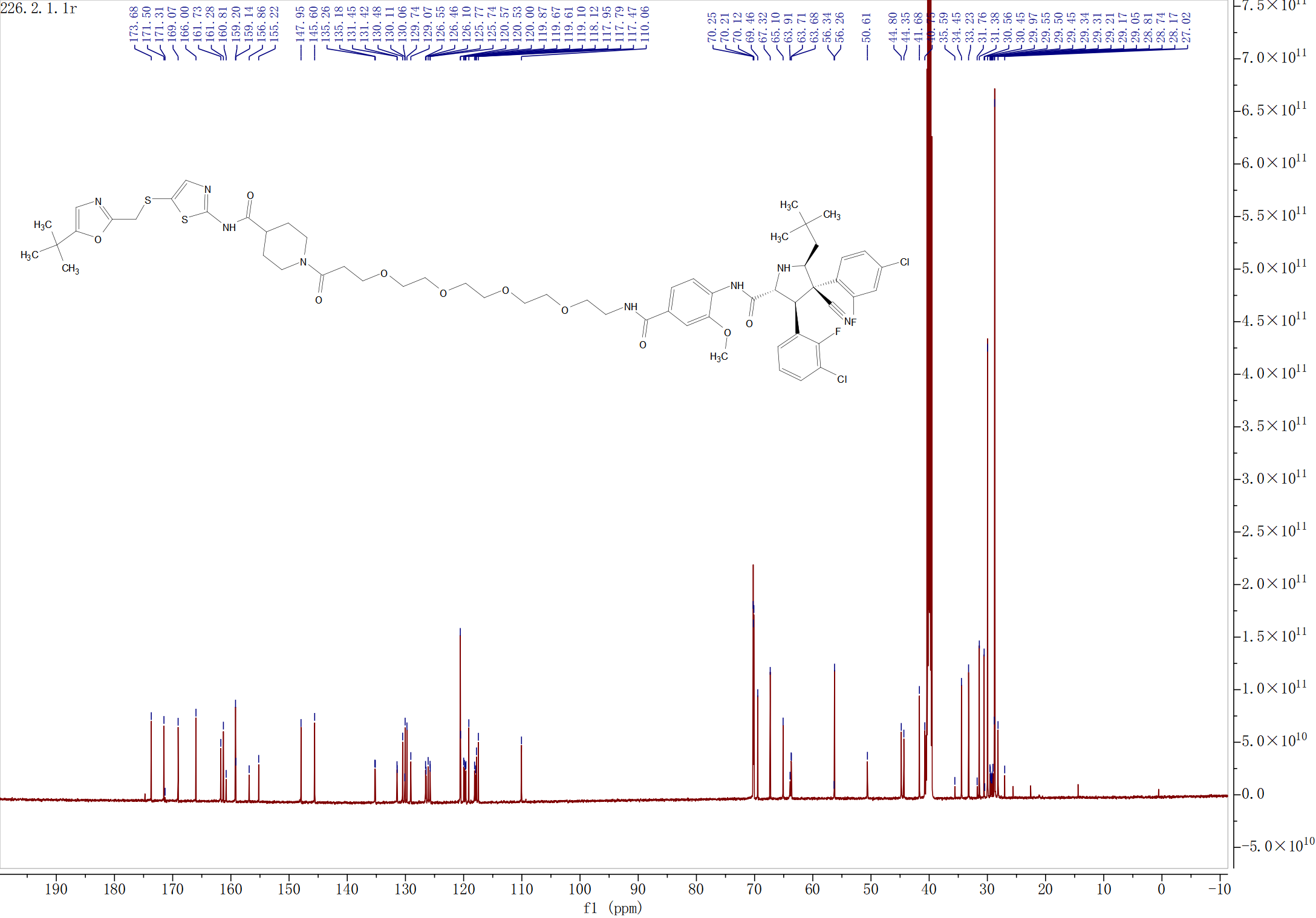
**

^13^C NMR Spectra of **14** (**dCDK9**-**012**) in DMSO-*d*_6_

**
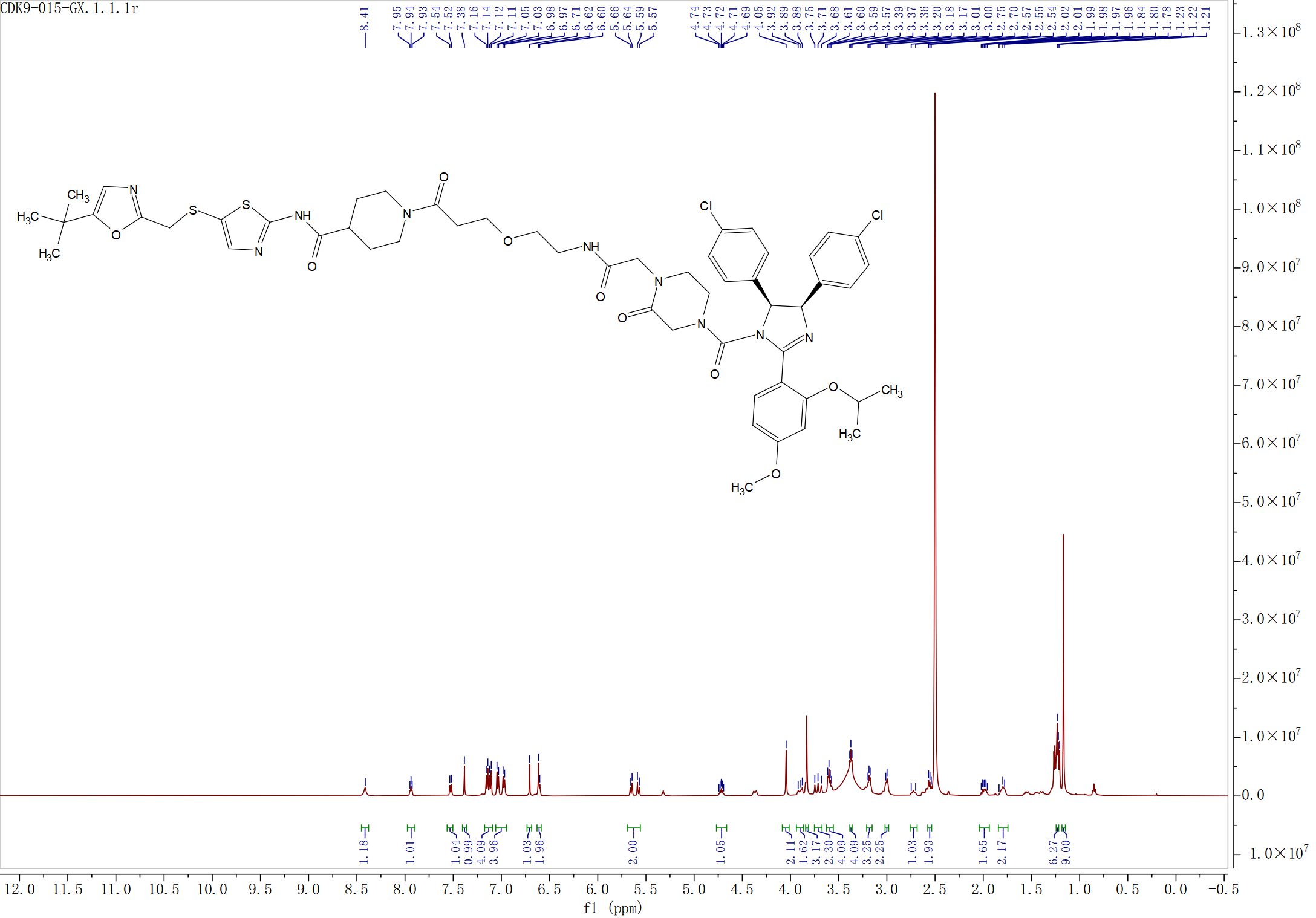
**

^1^H NMR Spectra of **15** (**dCDK9**-**015**) in DMSO-*d*_6_

**
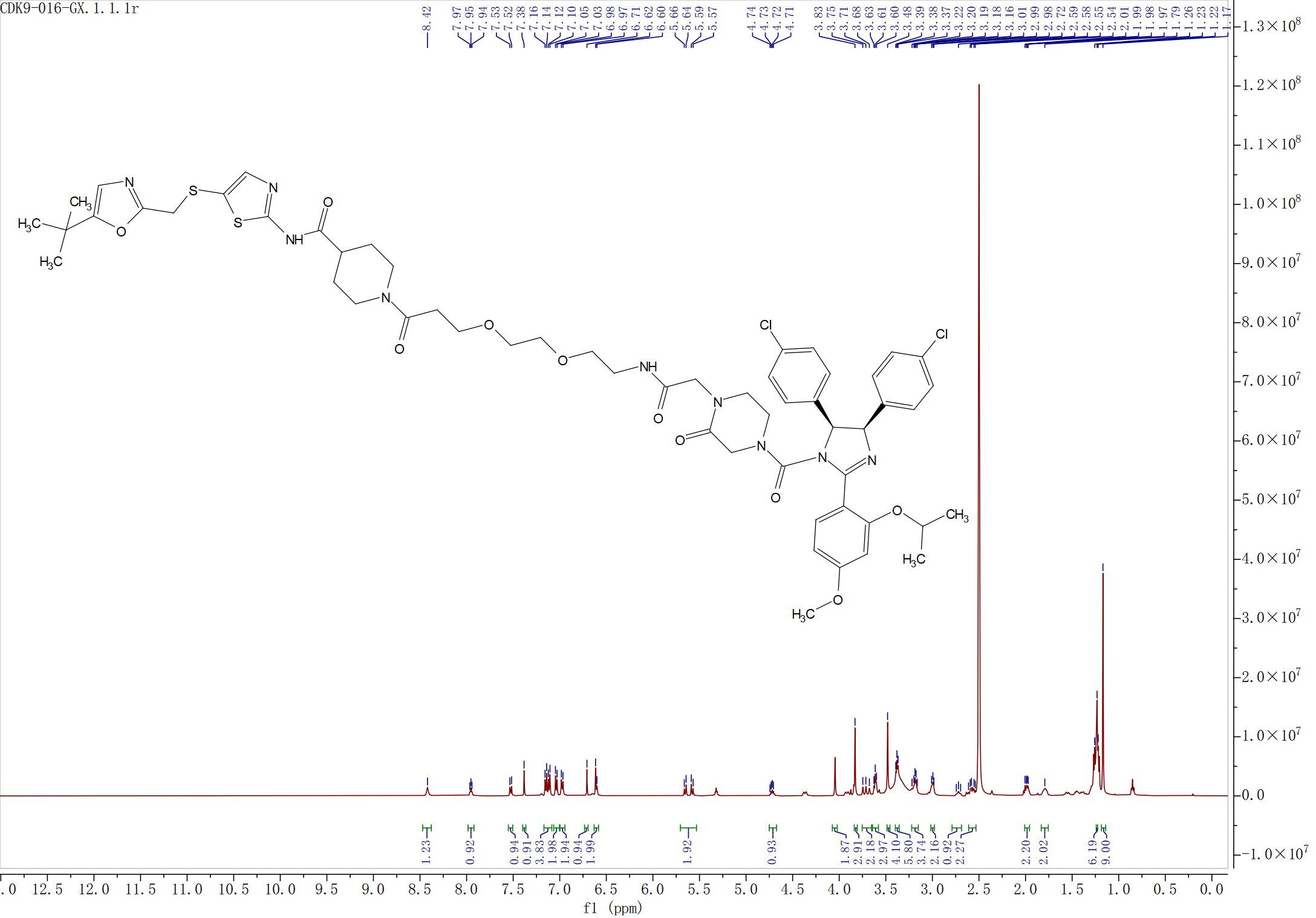
**

^1^H NMR Spectra of **16** (**dCDK9**-**016**) in DMSO-*d*_6_

**
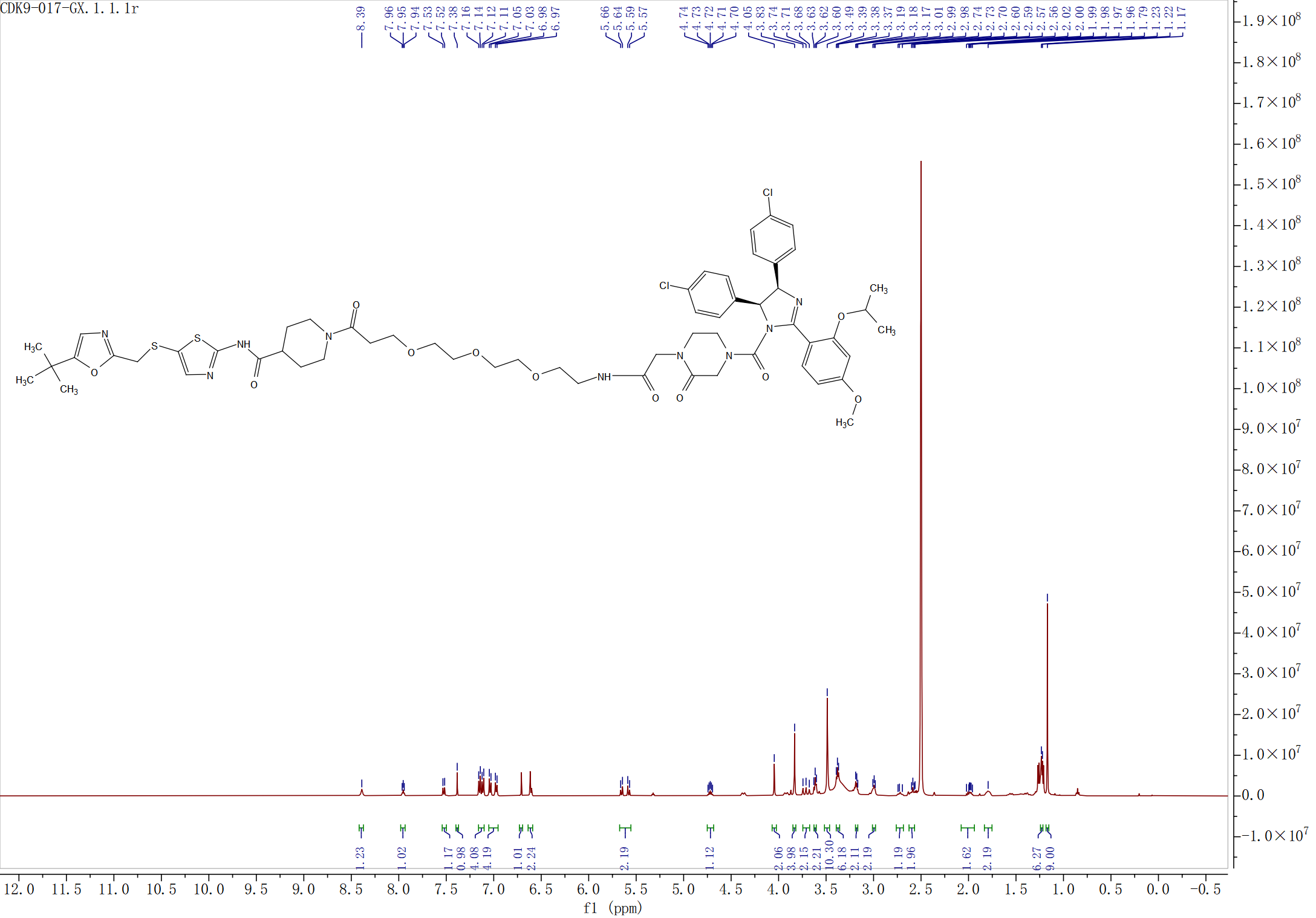
**

^1^H NMR Spectra of **17** (**dCDK9**-**017**) in DMSO-*d*_6_

**
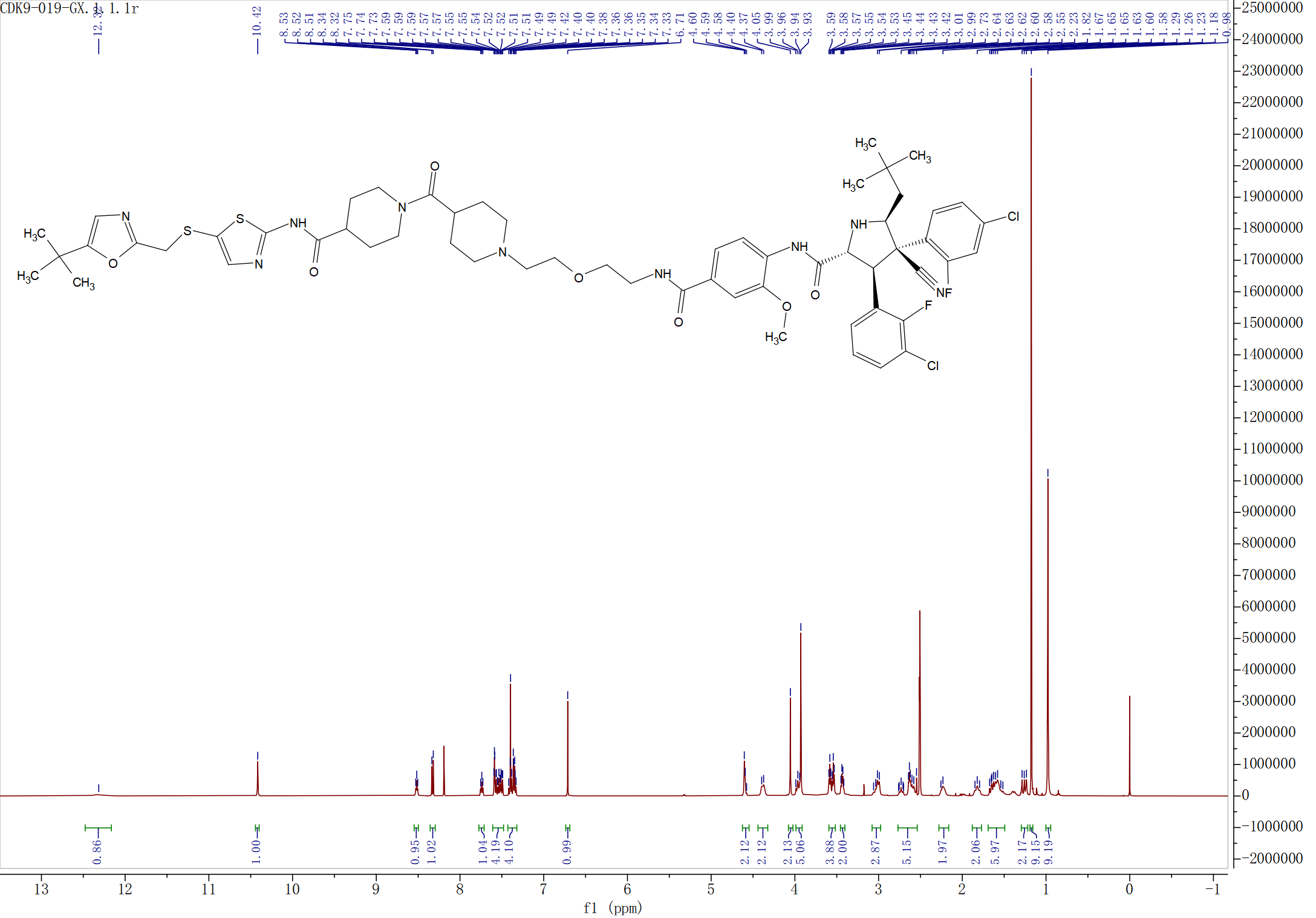
**

^1^H NMR Spectra of **18** (**dCDK9**-**019**) in DMSO-*d*_6_

**
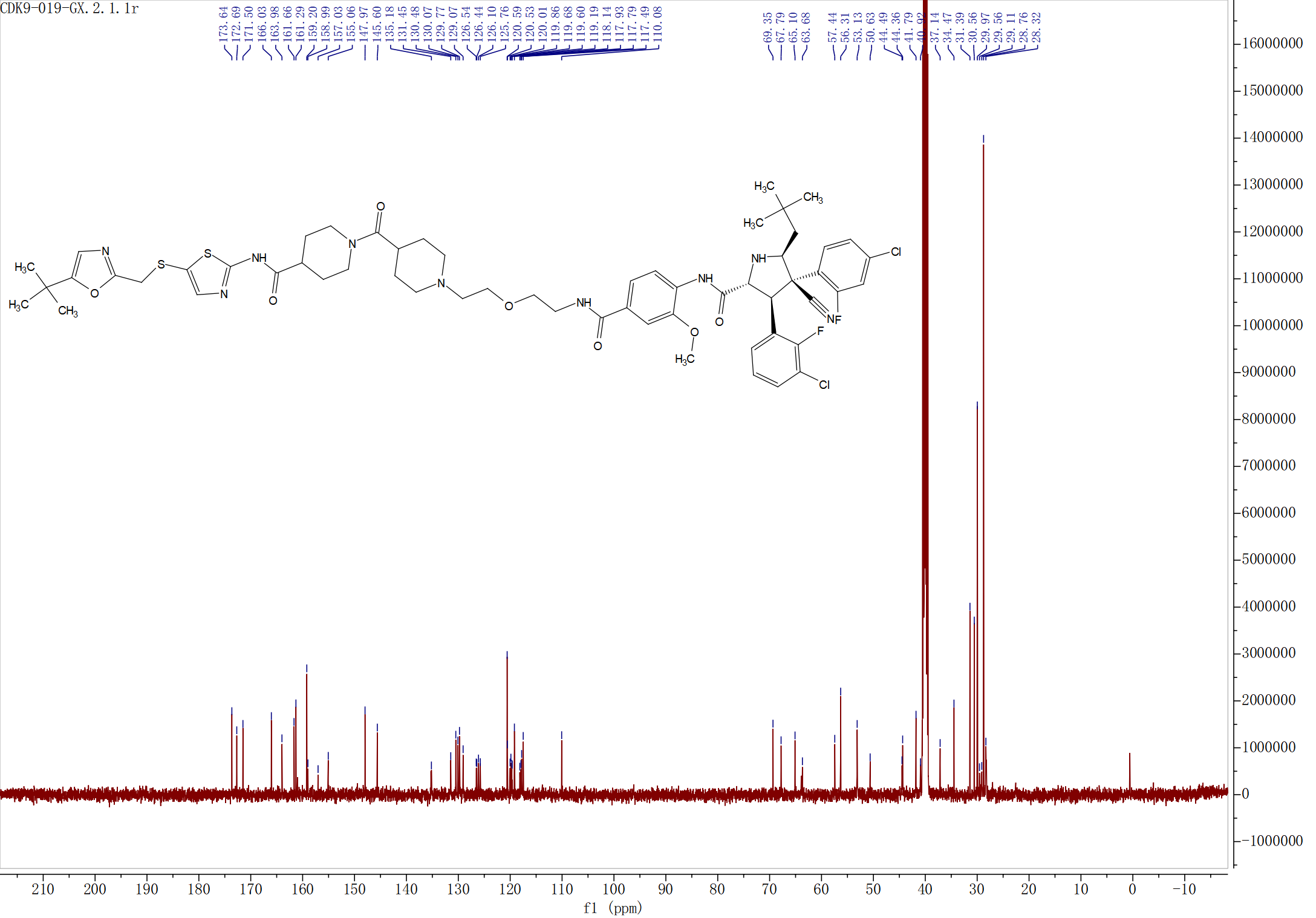
**

^13^C NMR Spectra of **18** (**dCDK9**-**019**) in DMSO-*d*_6_

**
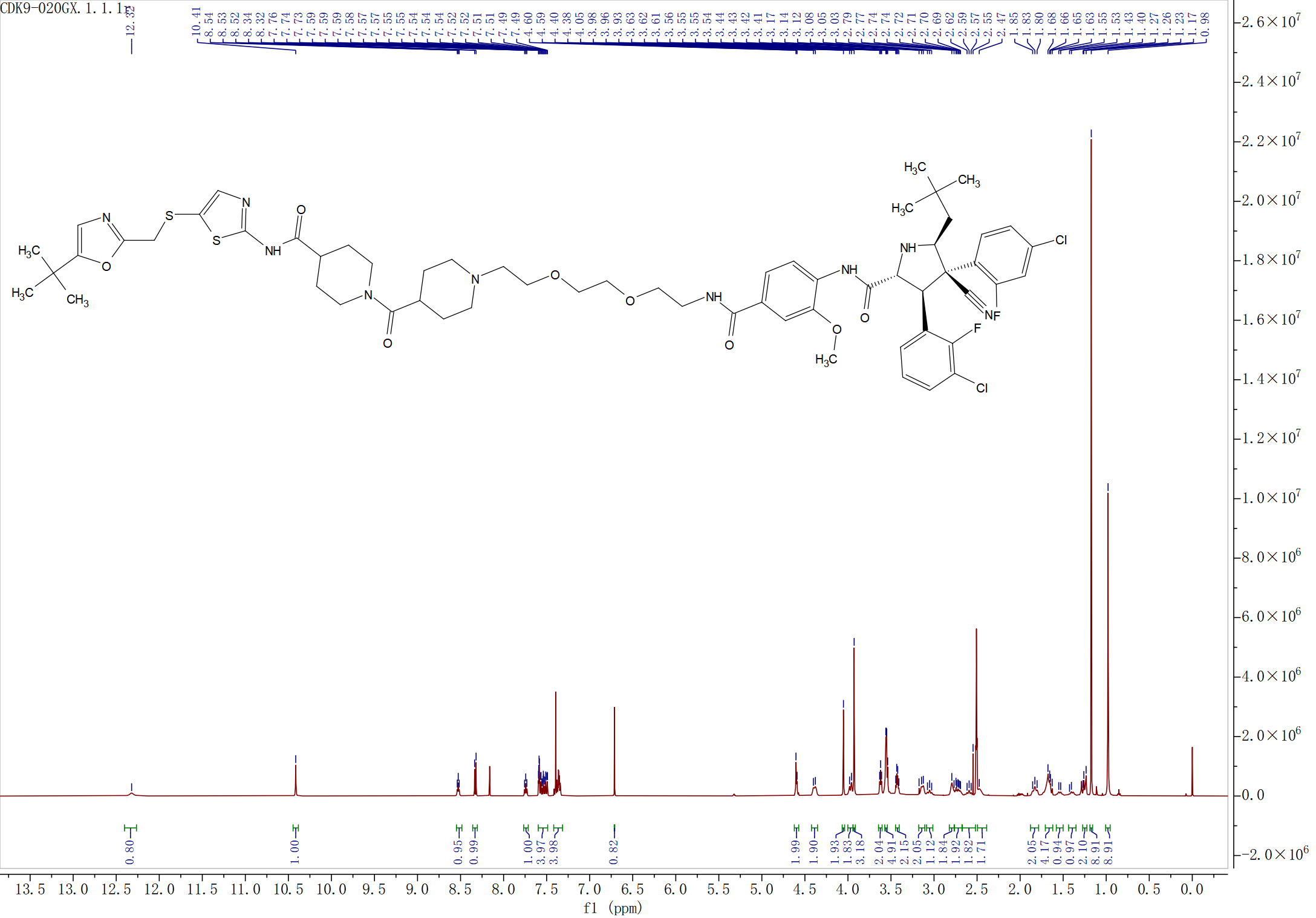
**

^1^H NMR Spectra of **19** (**dCDK9**-**020**) in DMSO-*d*_6_

**
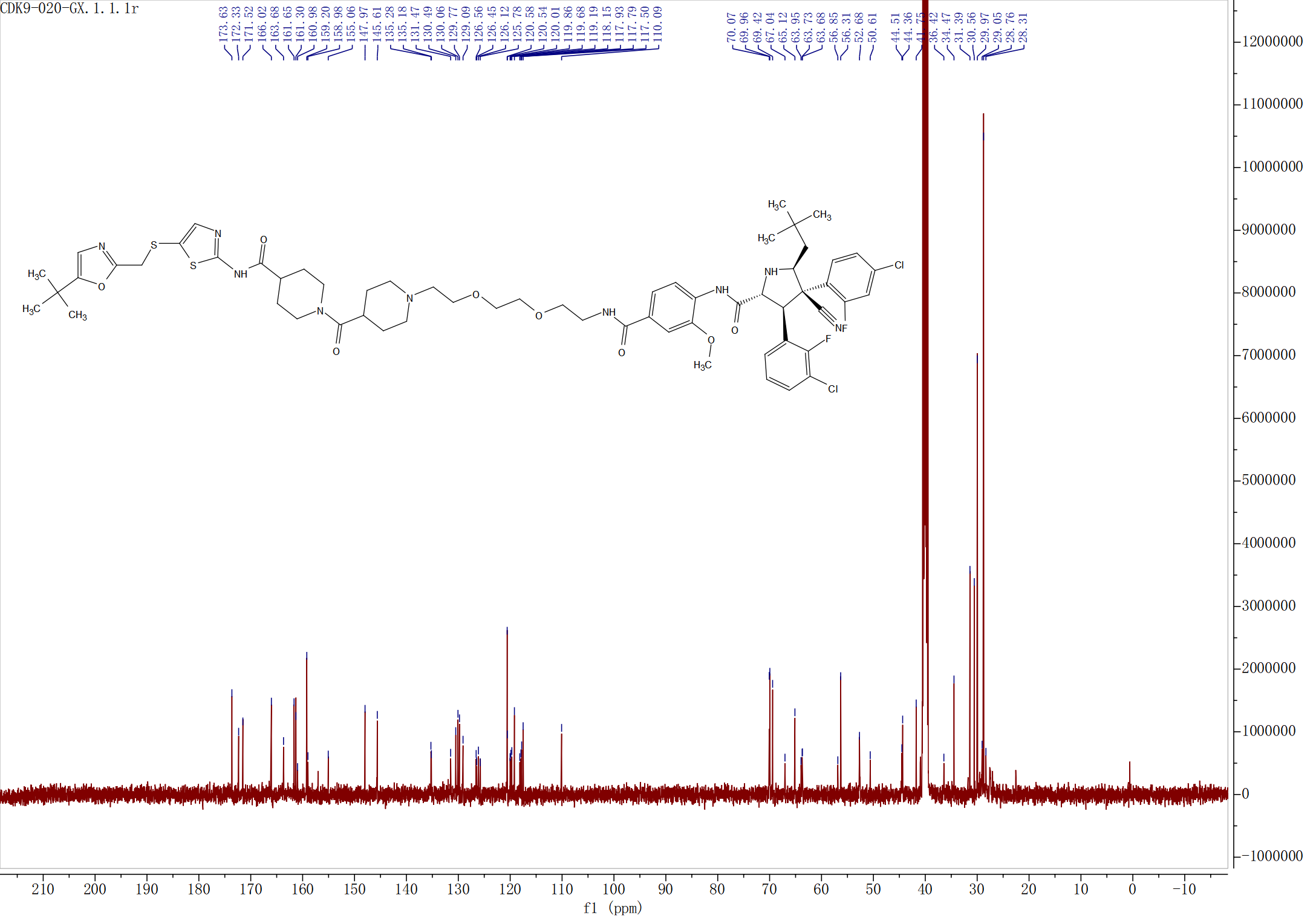
**

^13^C NMR Spectra of **19** (**dCDK9**-**020**) in DMSO-*d*_6_


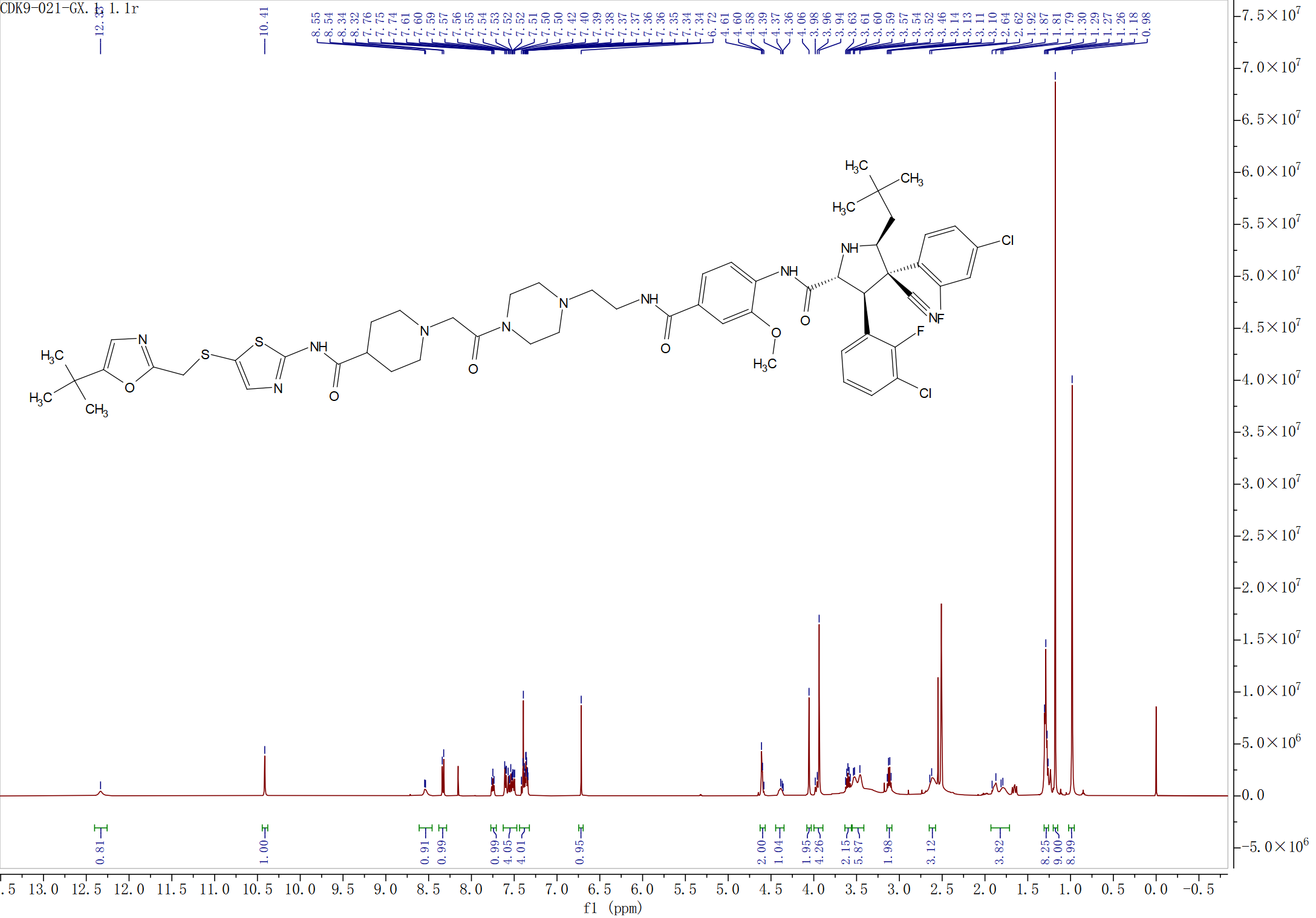


^1^H NMR Spectra of **20** (**dCDK9**-**021**) in DMSO-*d*_6_

**
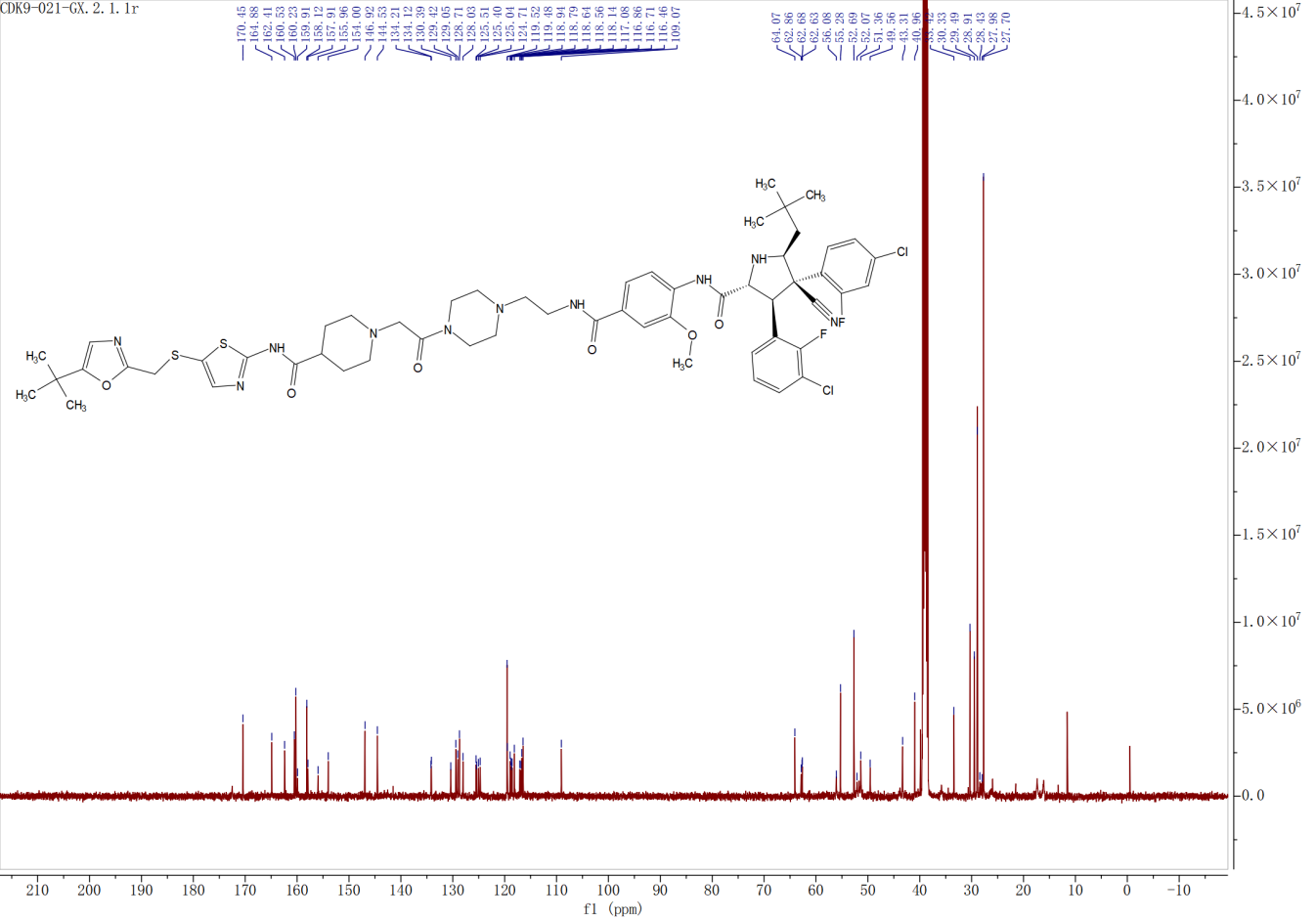
**

^13^C NMR Spectra of **20** (**dCDK9**-**021**) in DMSO-*d*_6_

**
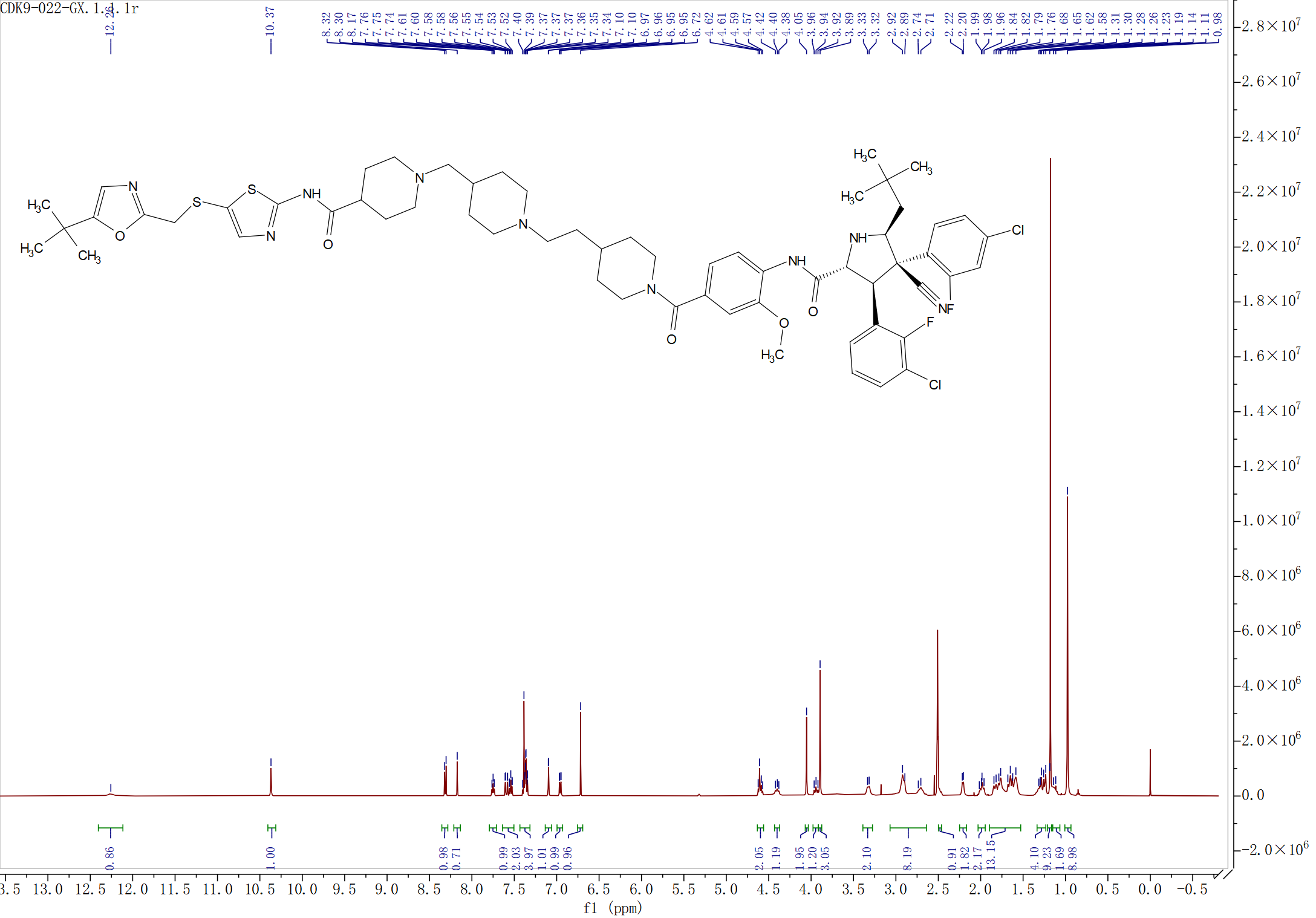
**

^1^H NMR Spectra of **21** (**dCDK9**-**022**) in DMSO-*d*_6_

**
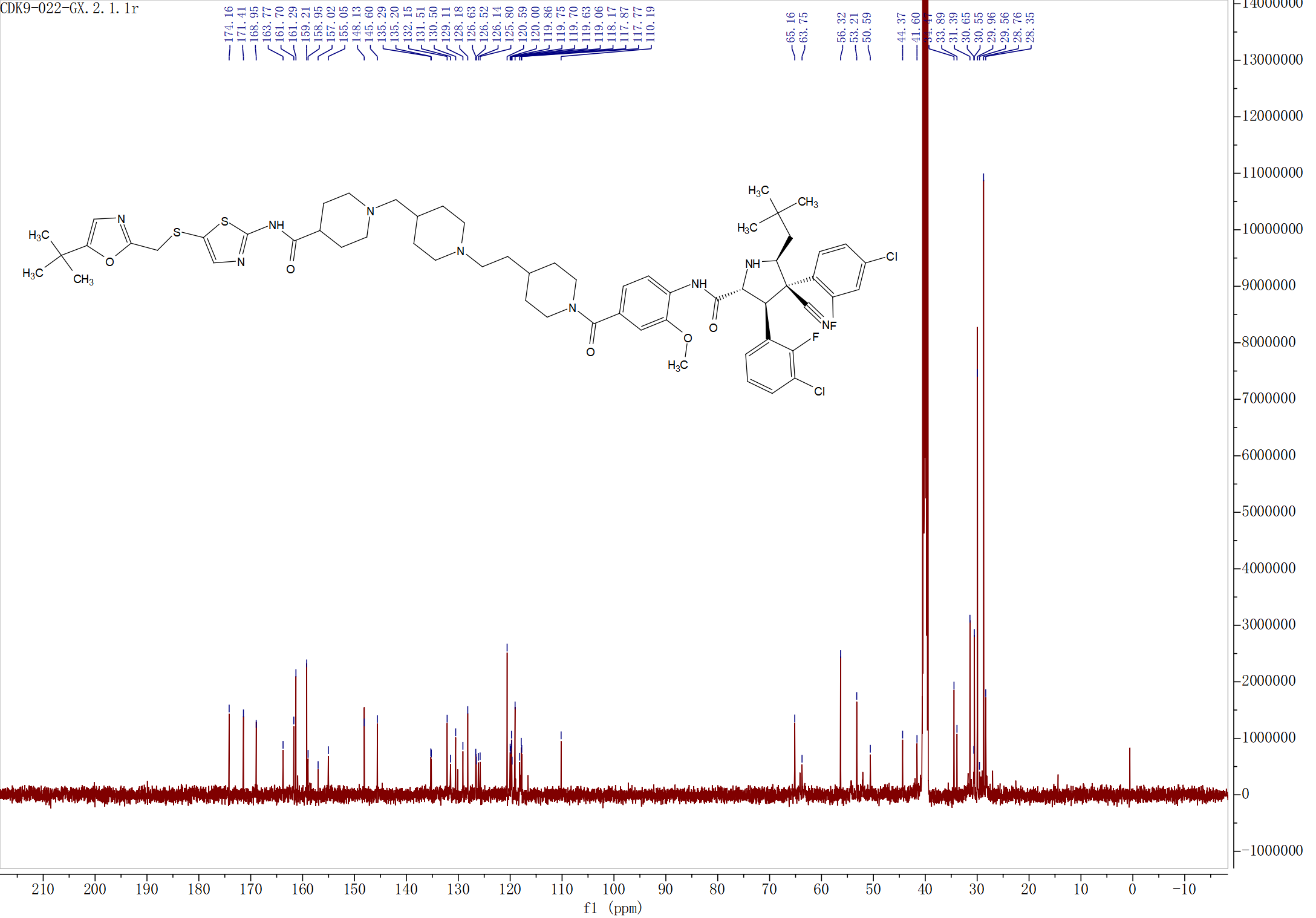
**

^13^C NMR Spectra of **21** (**dCDK9**-**022**) in DMSO-*d*_6_

**
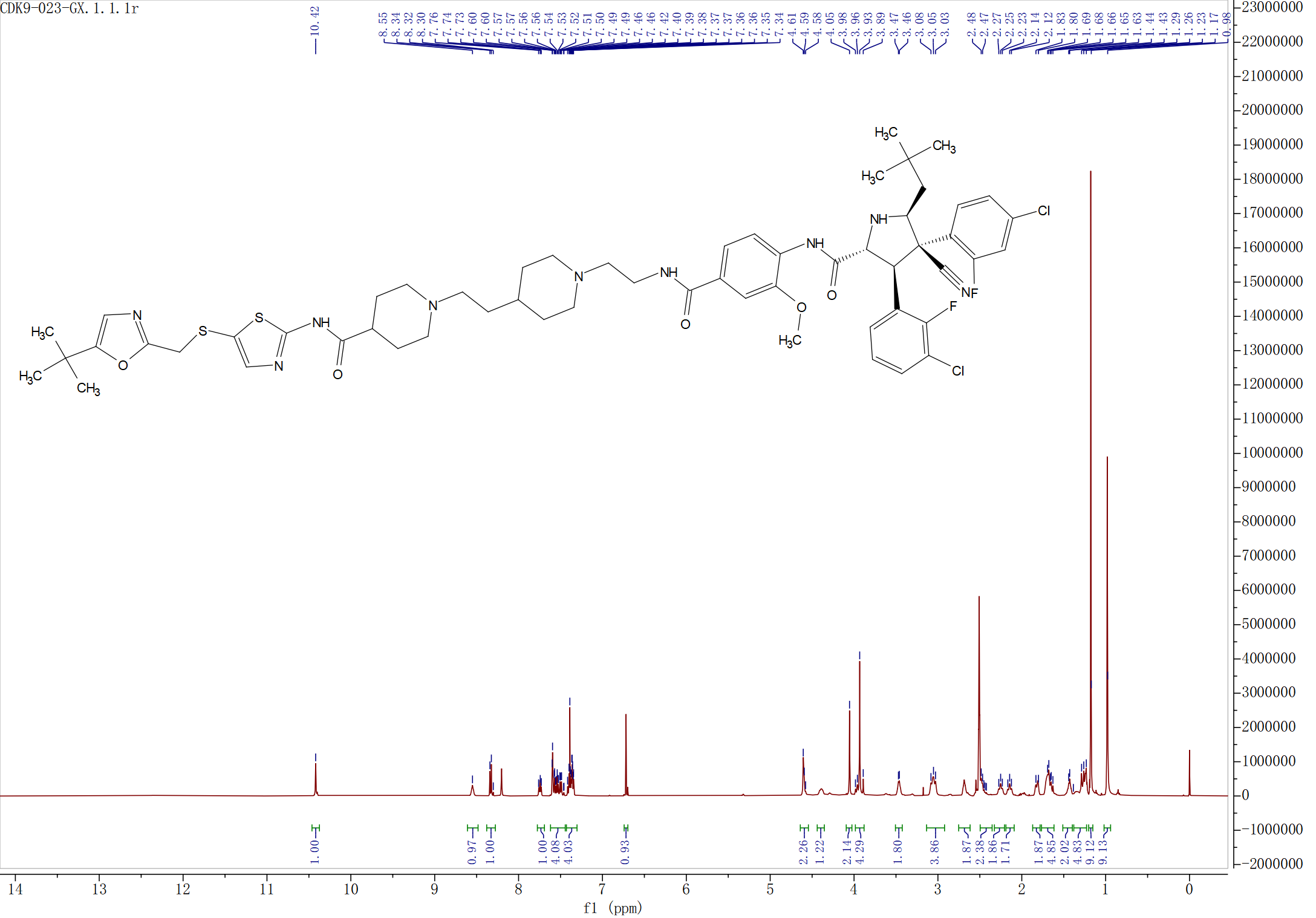
**

^1^H NMR Spectra of **22** (**dCDK9**-**023**) in DMSO-*d*_6_

**
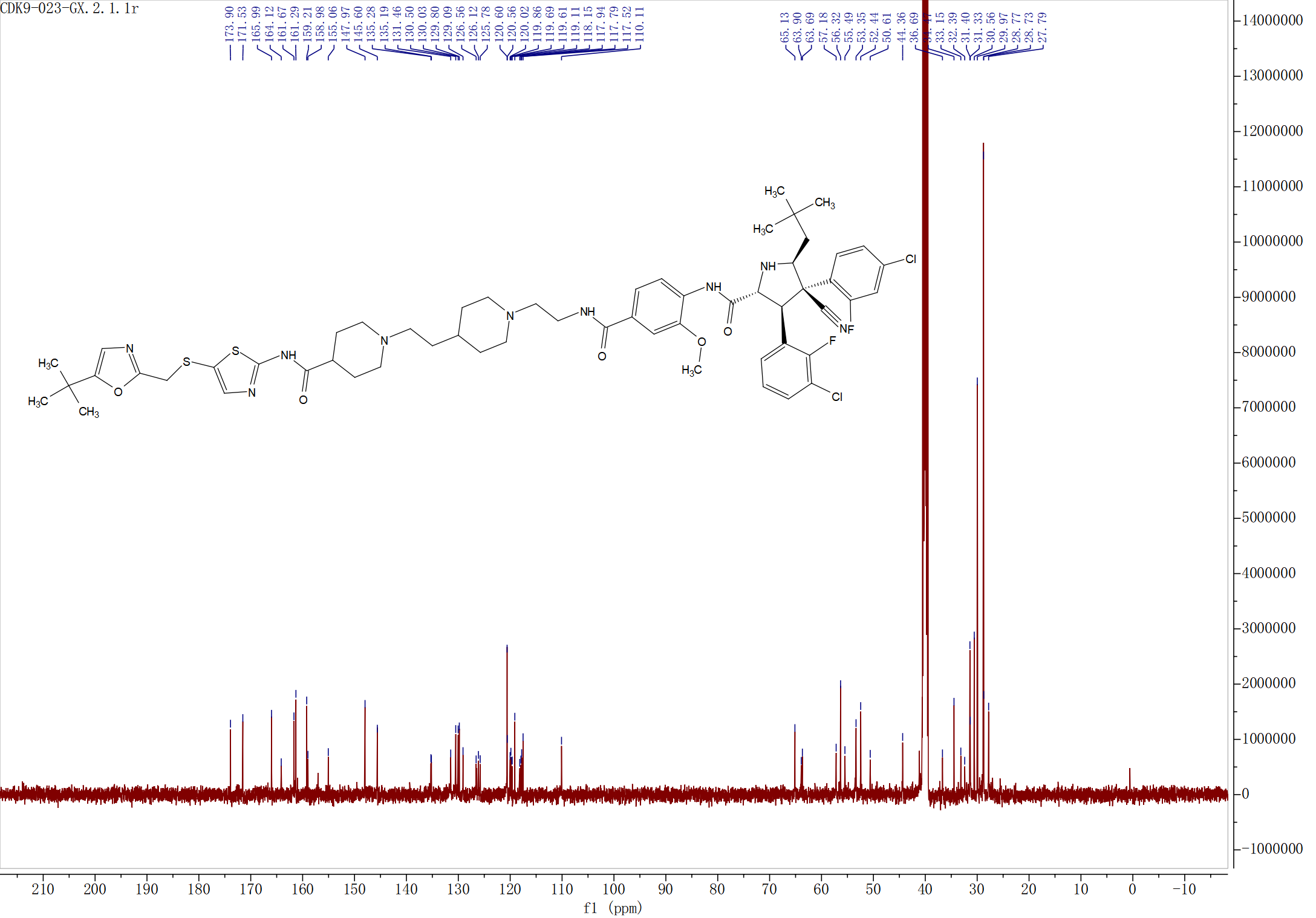
**

^13^C NMR Spectra of **22** (**dCDK9**-**023**) in DMSO-*d*_6_

**
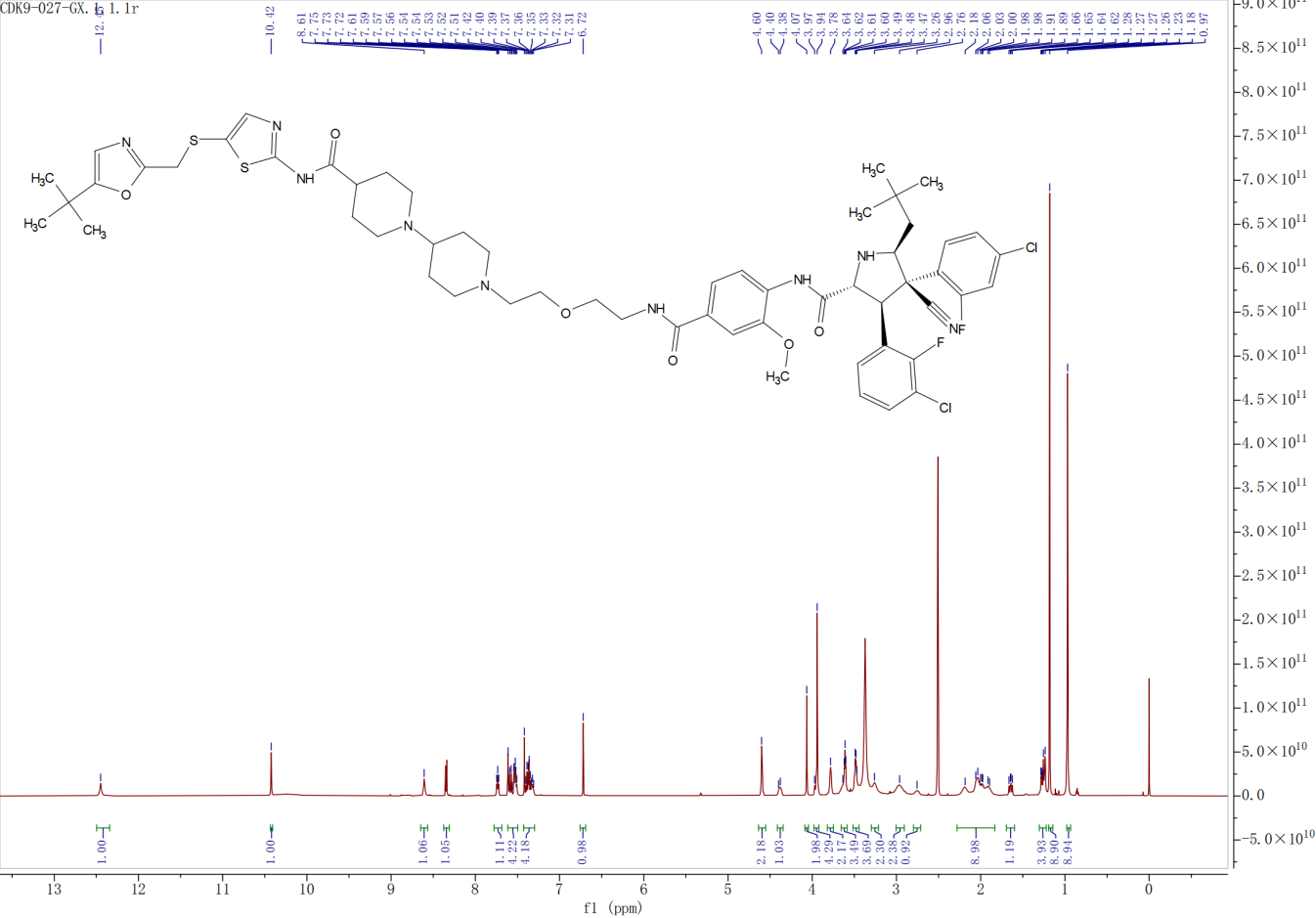
**

^1^H NMR Spectra of **23** (**dCDK9**-**027**) in DMSO-*d*_6_

**
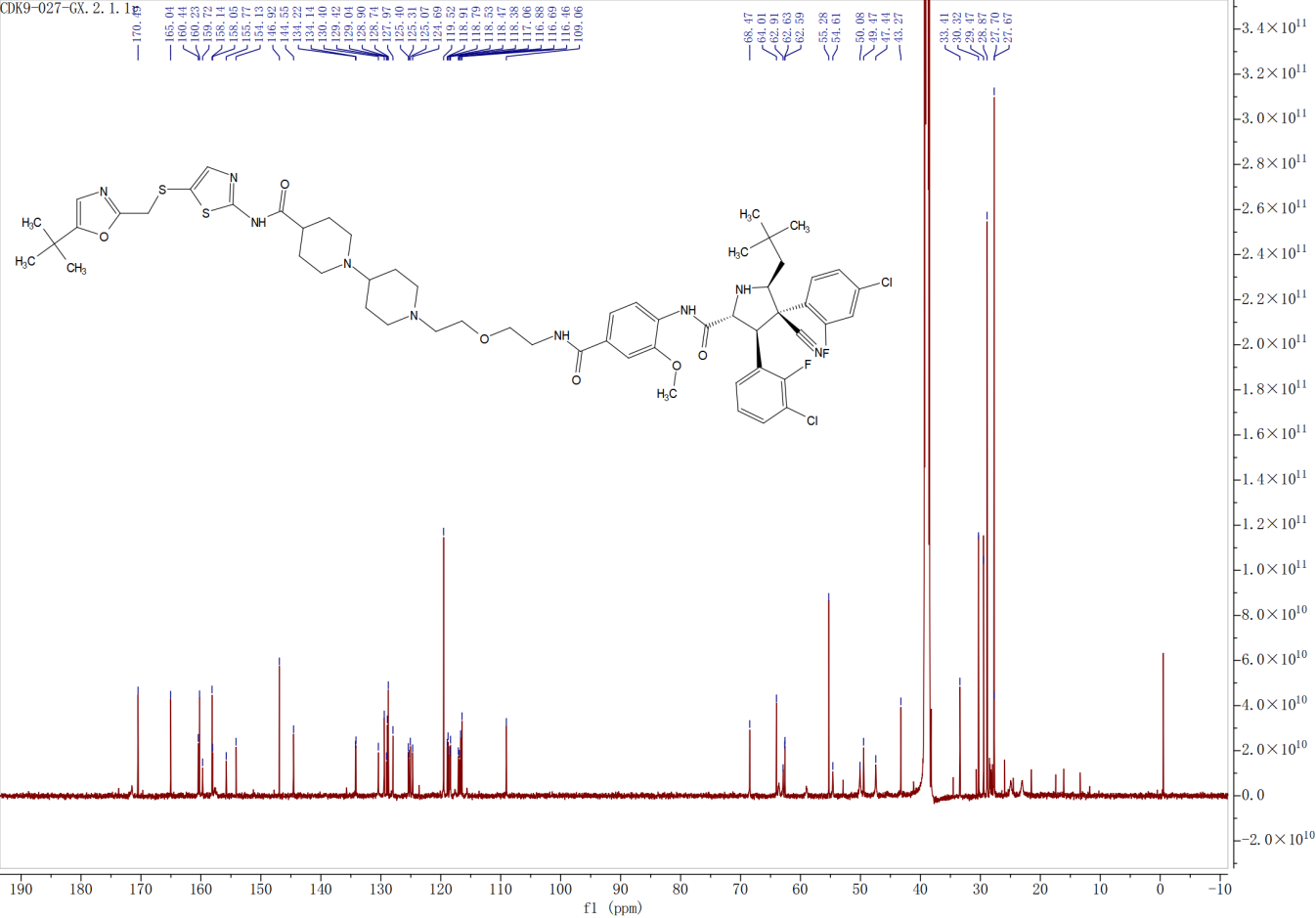
**

^13^C NMR Spectra of **23** (**dCDK9**-**027**) in DMSO-*d*_6_

**Part 4. UPLC-MS results**

**
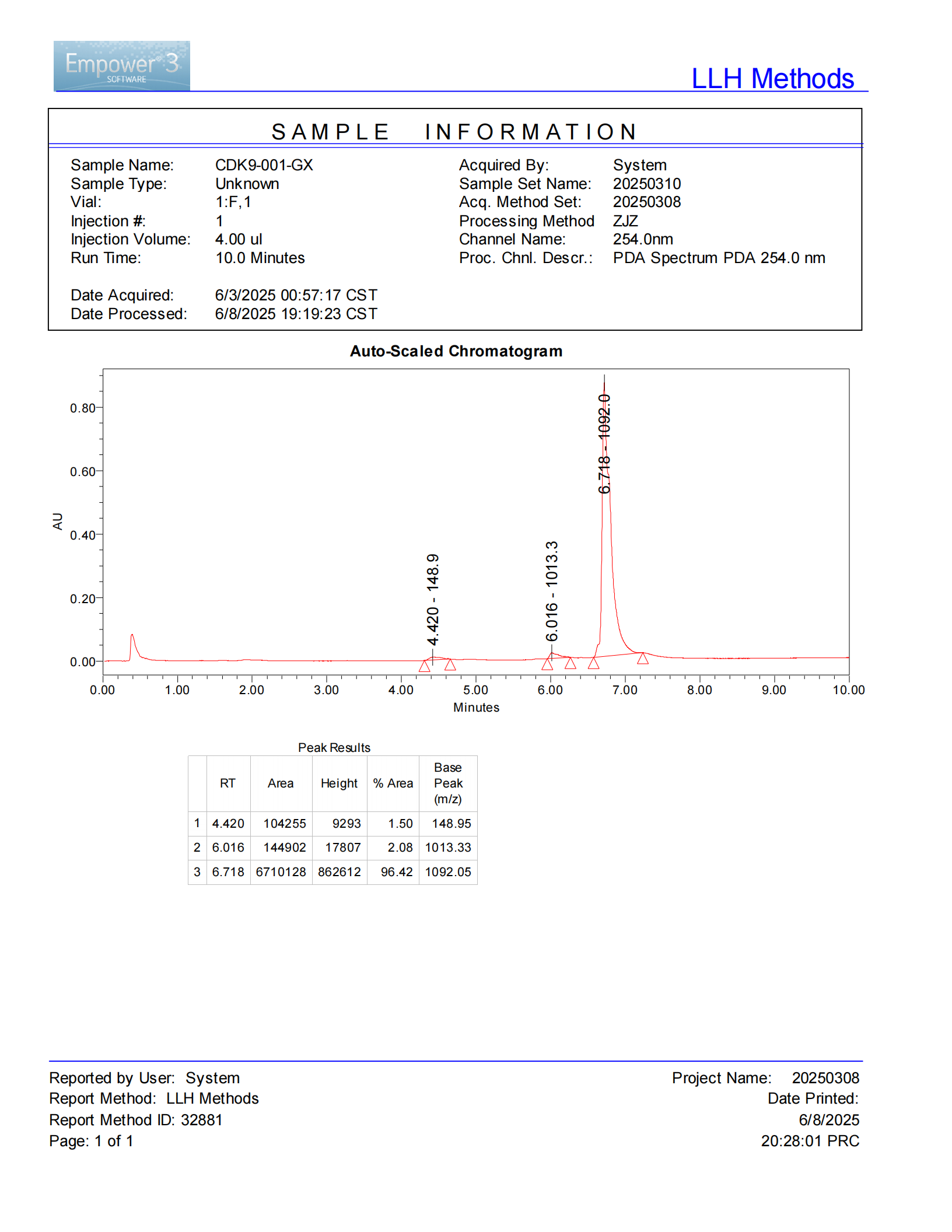
**

**
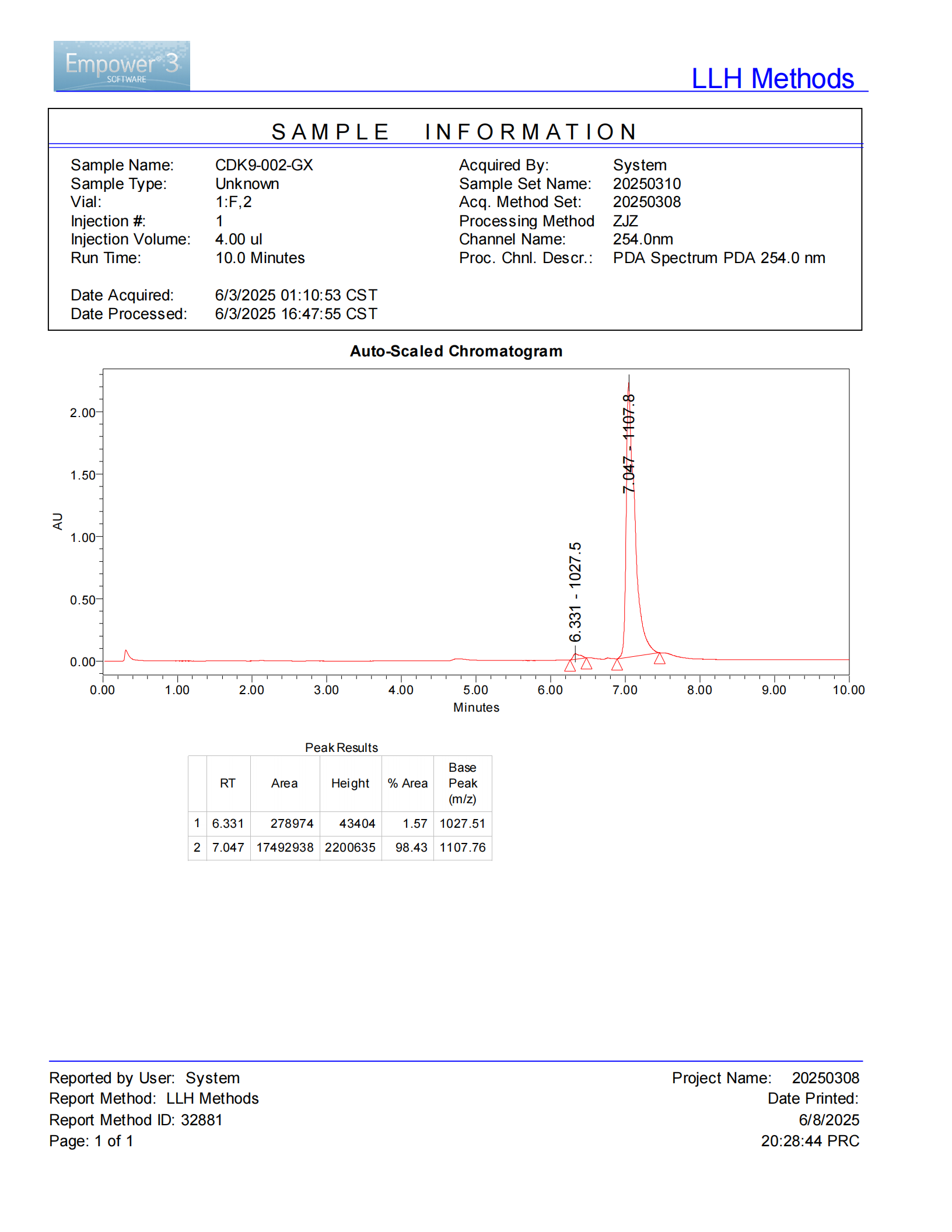
**

**
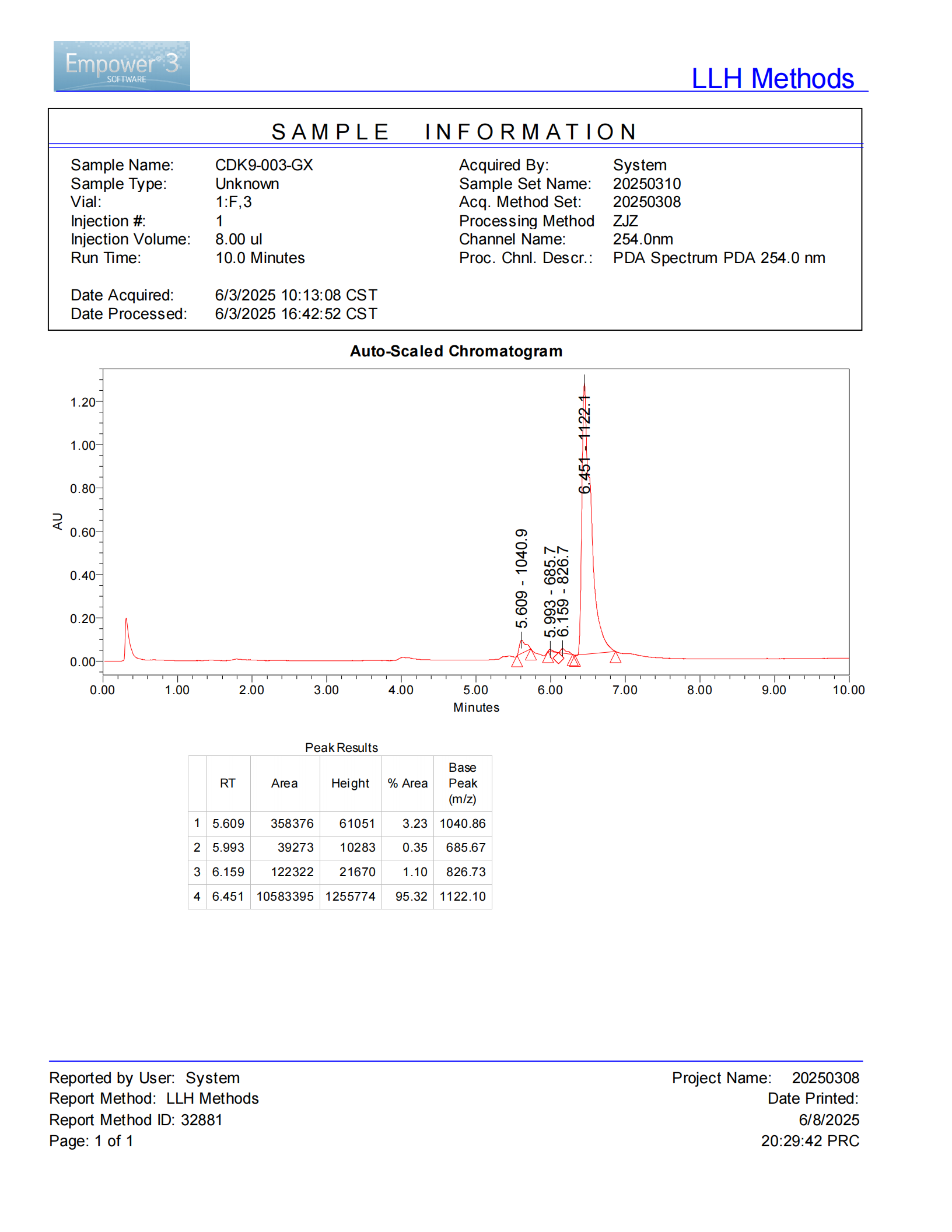
**

**
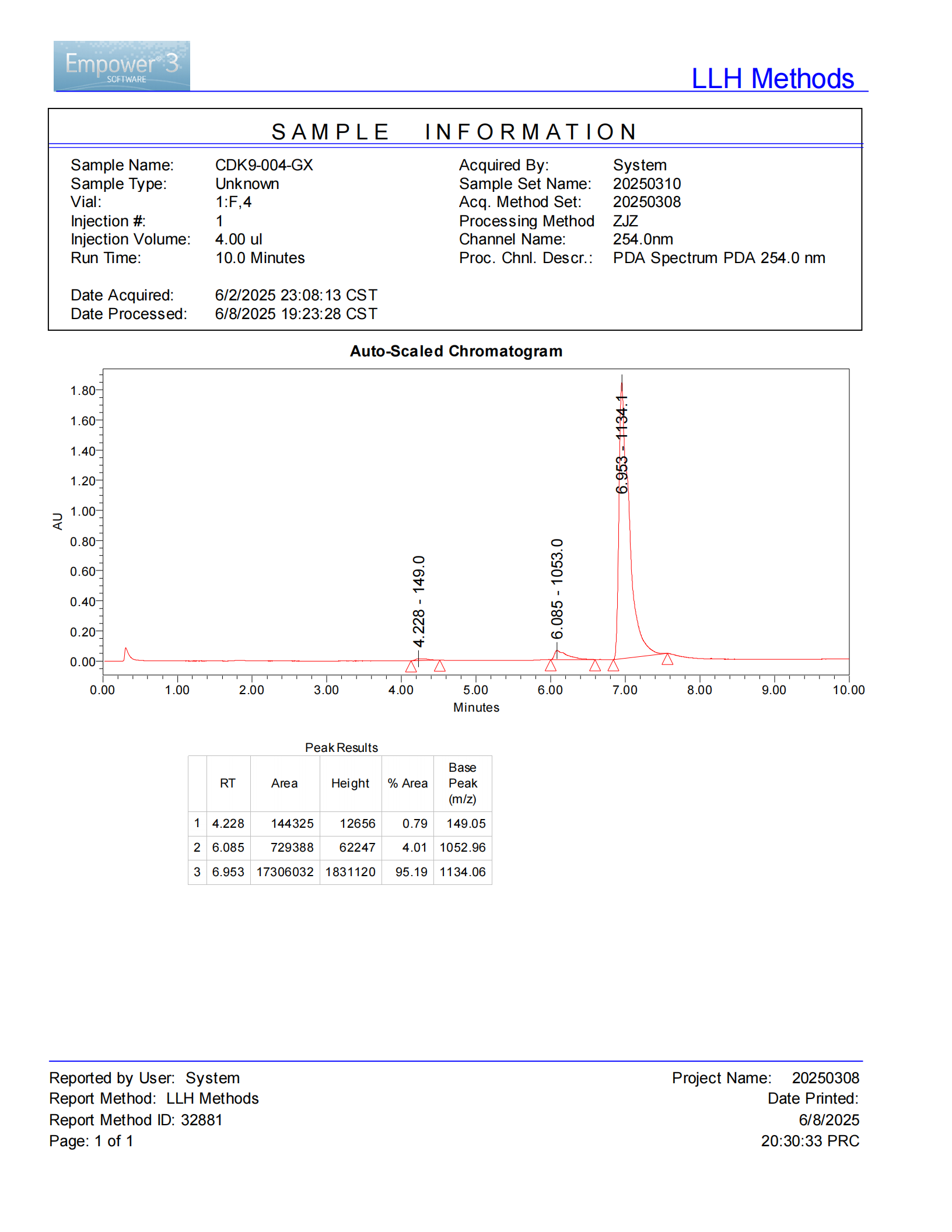
**

**
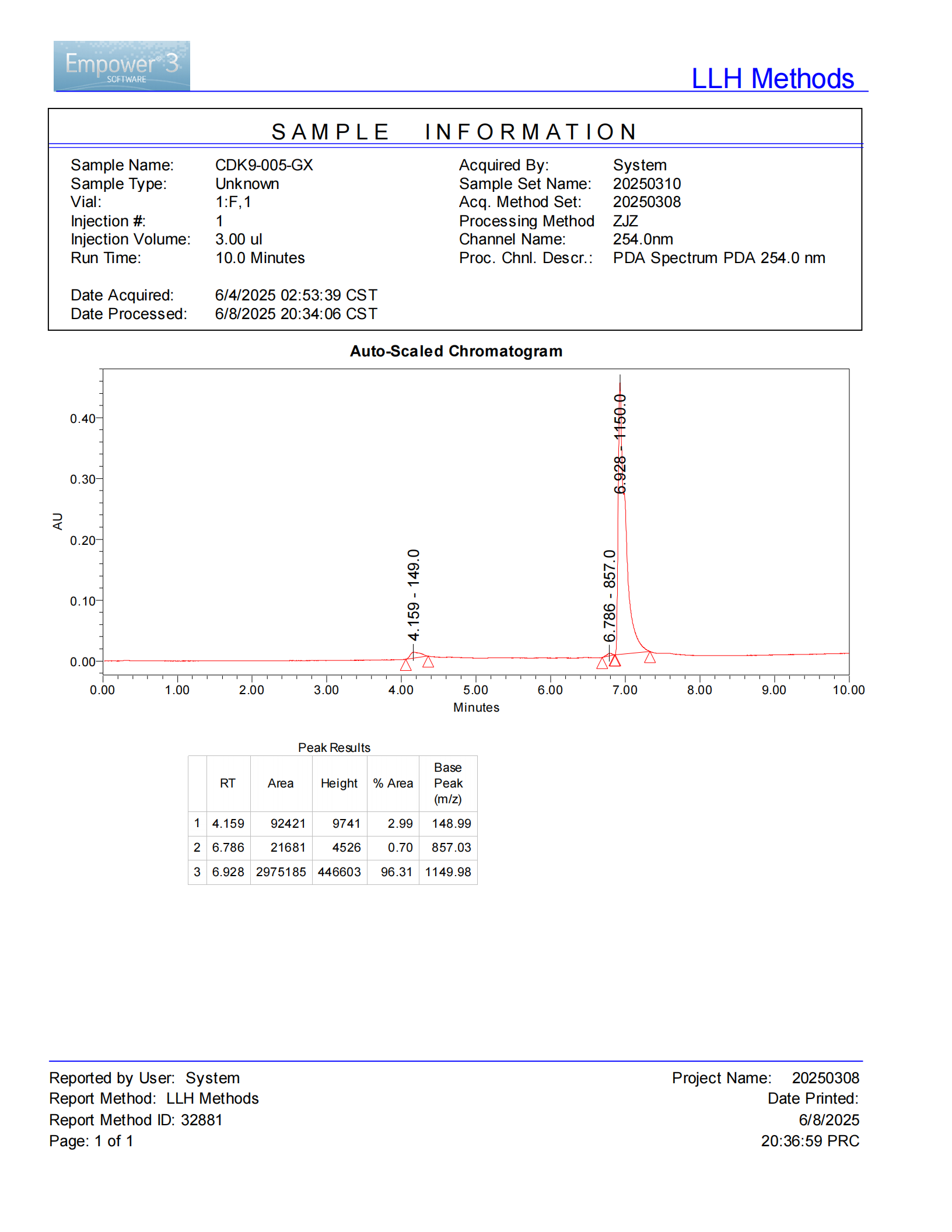
**

**
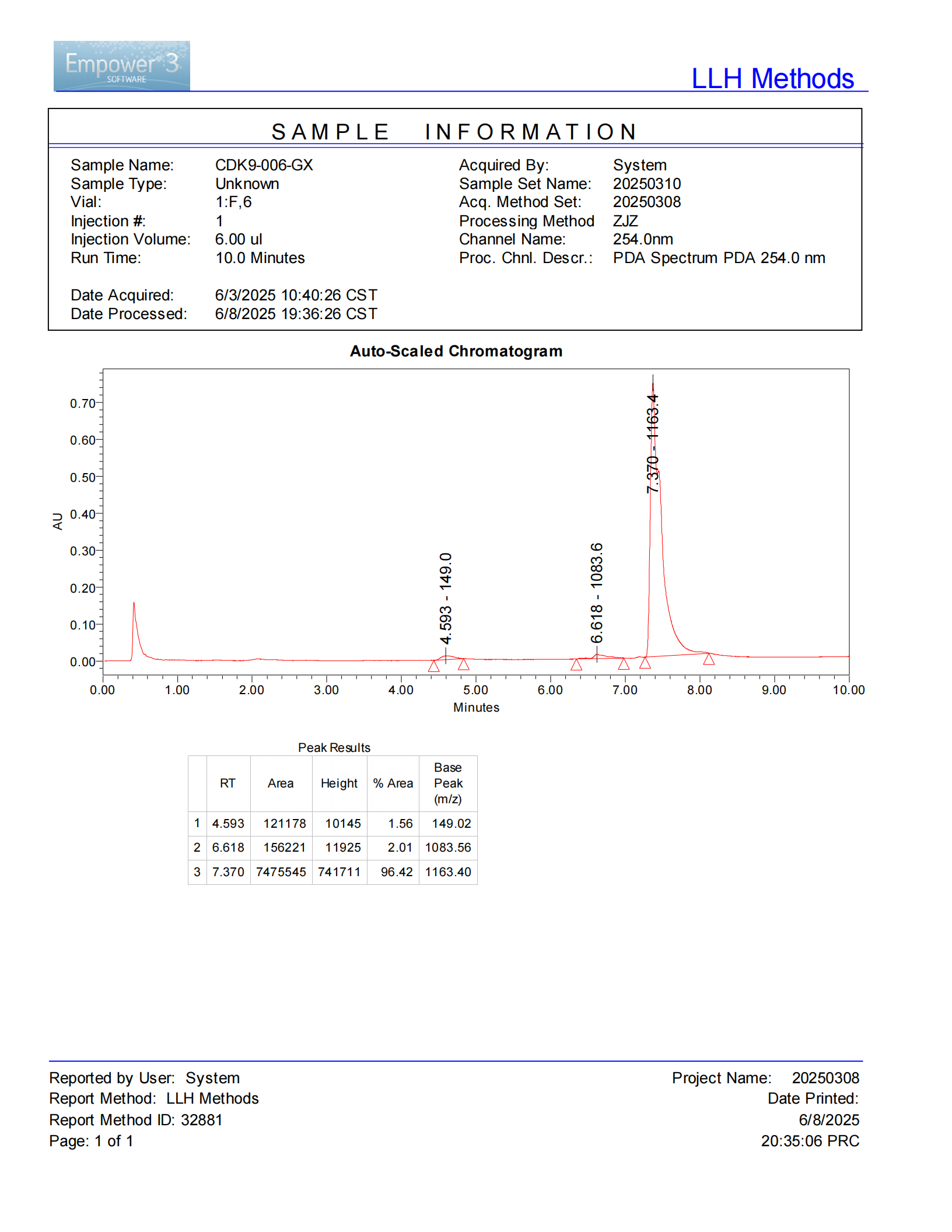
**

**
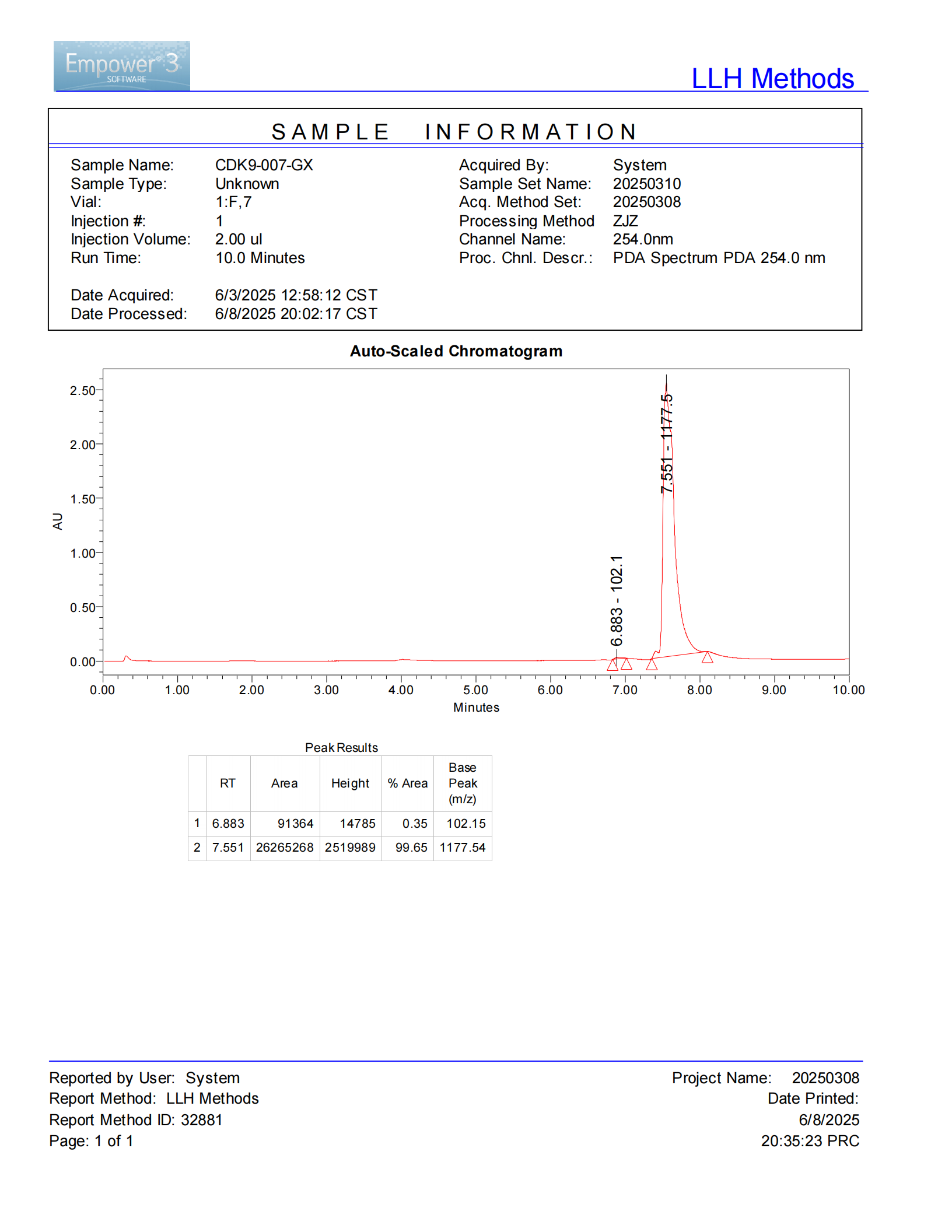
**

**
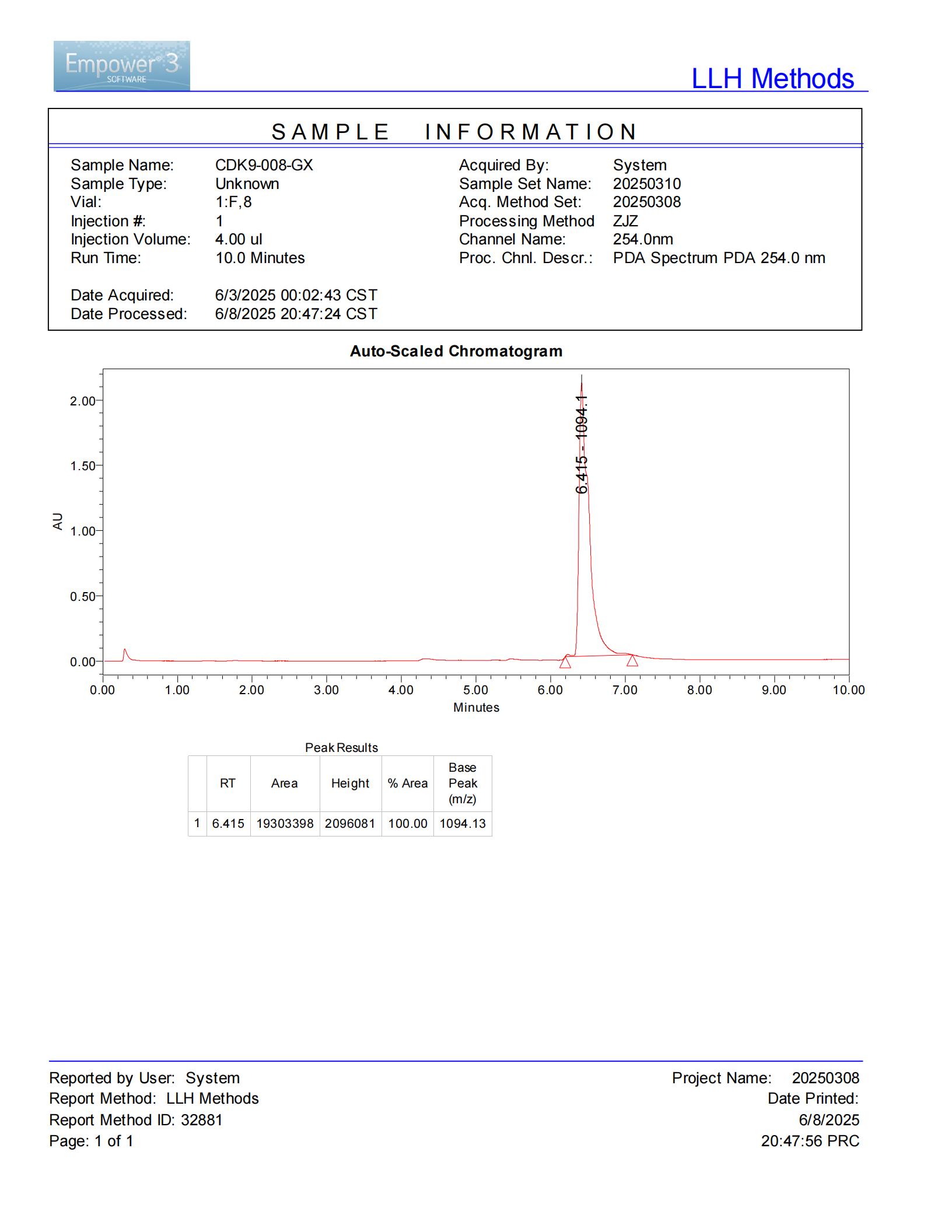
**

**
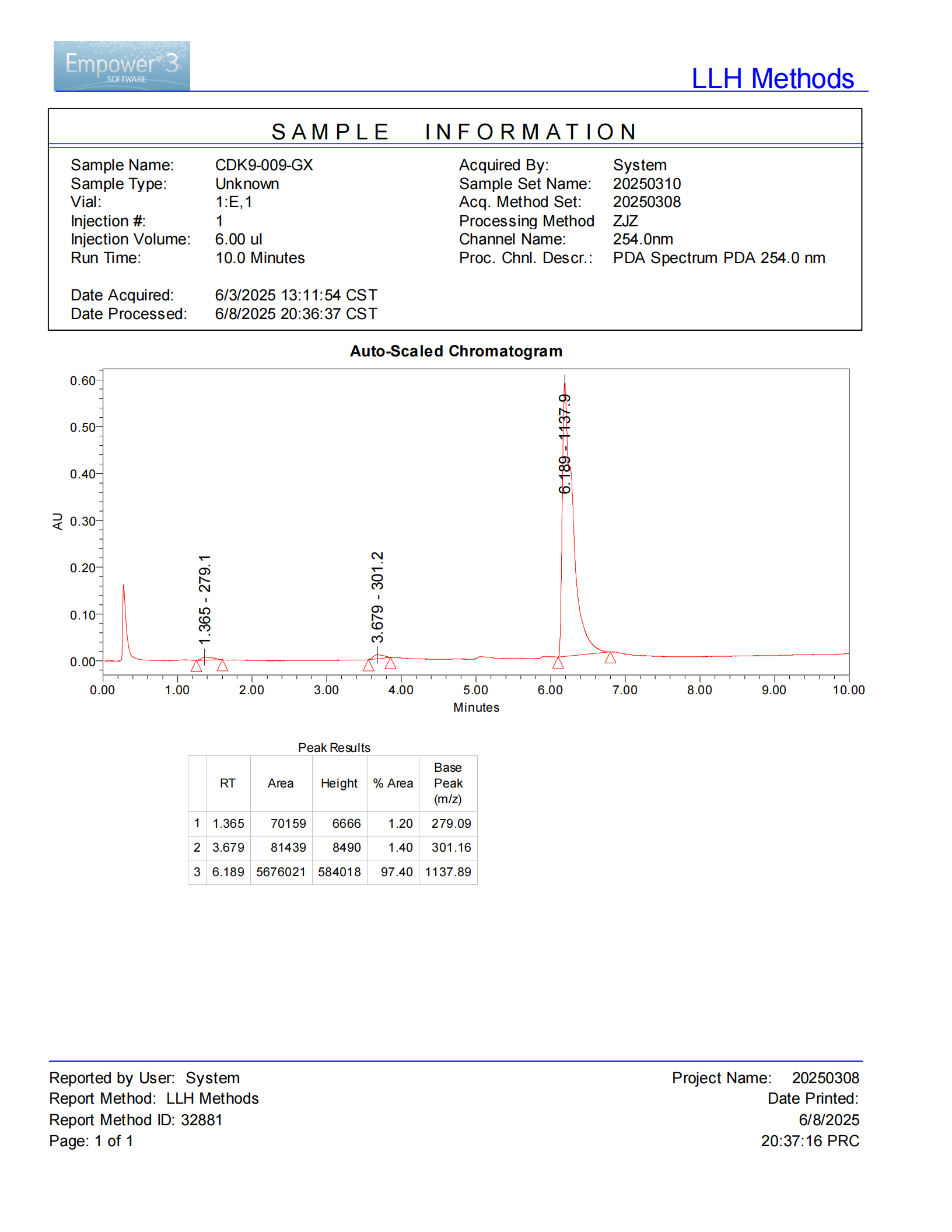

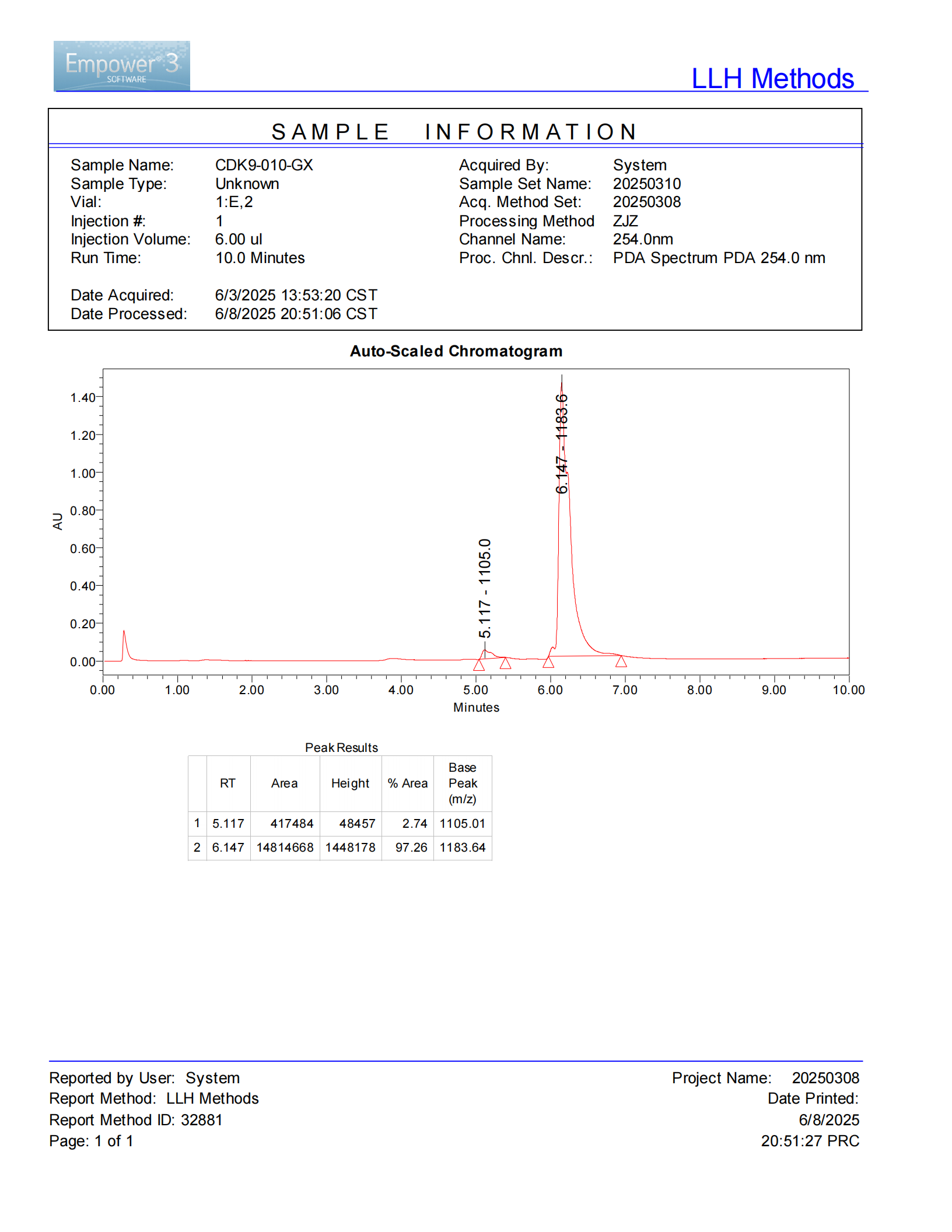

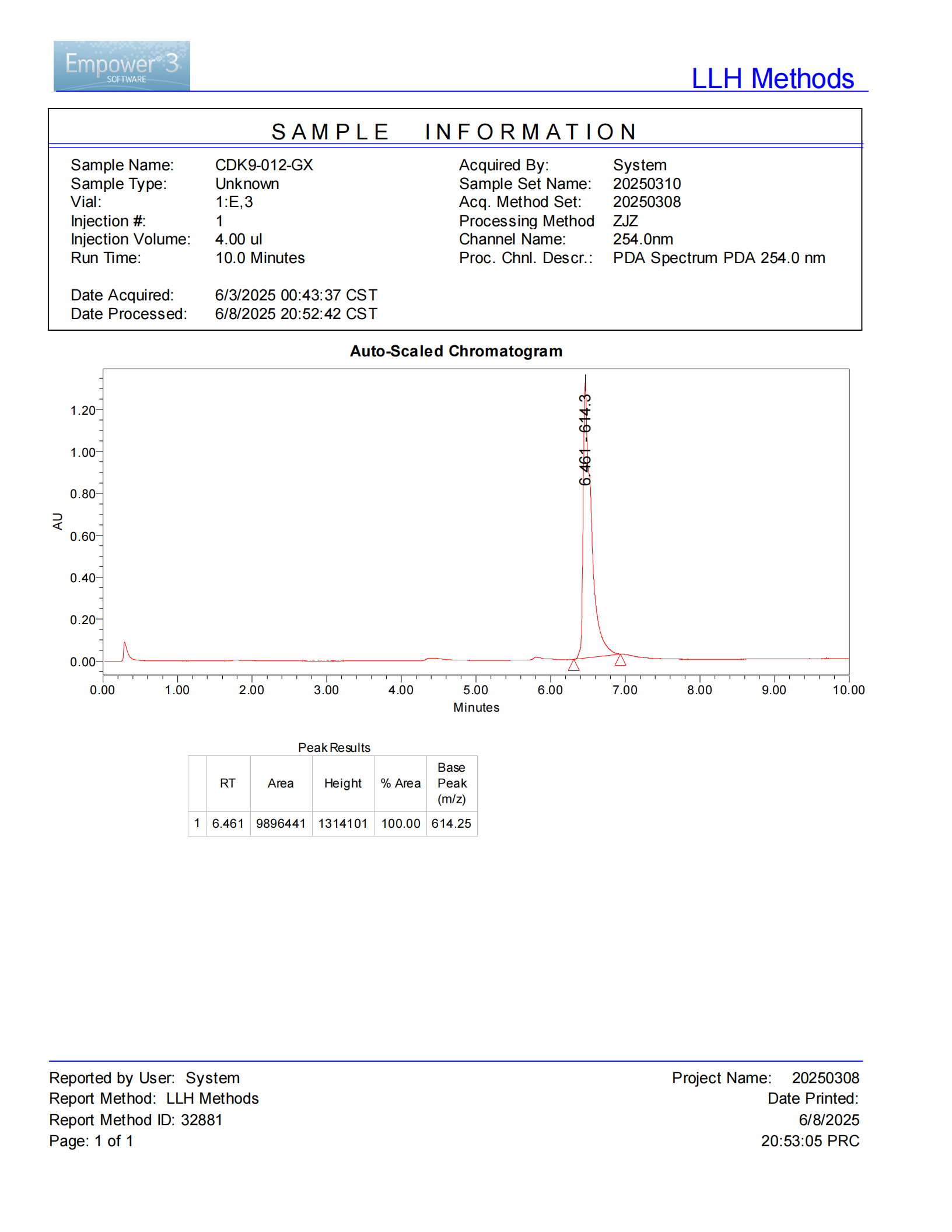

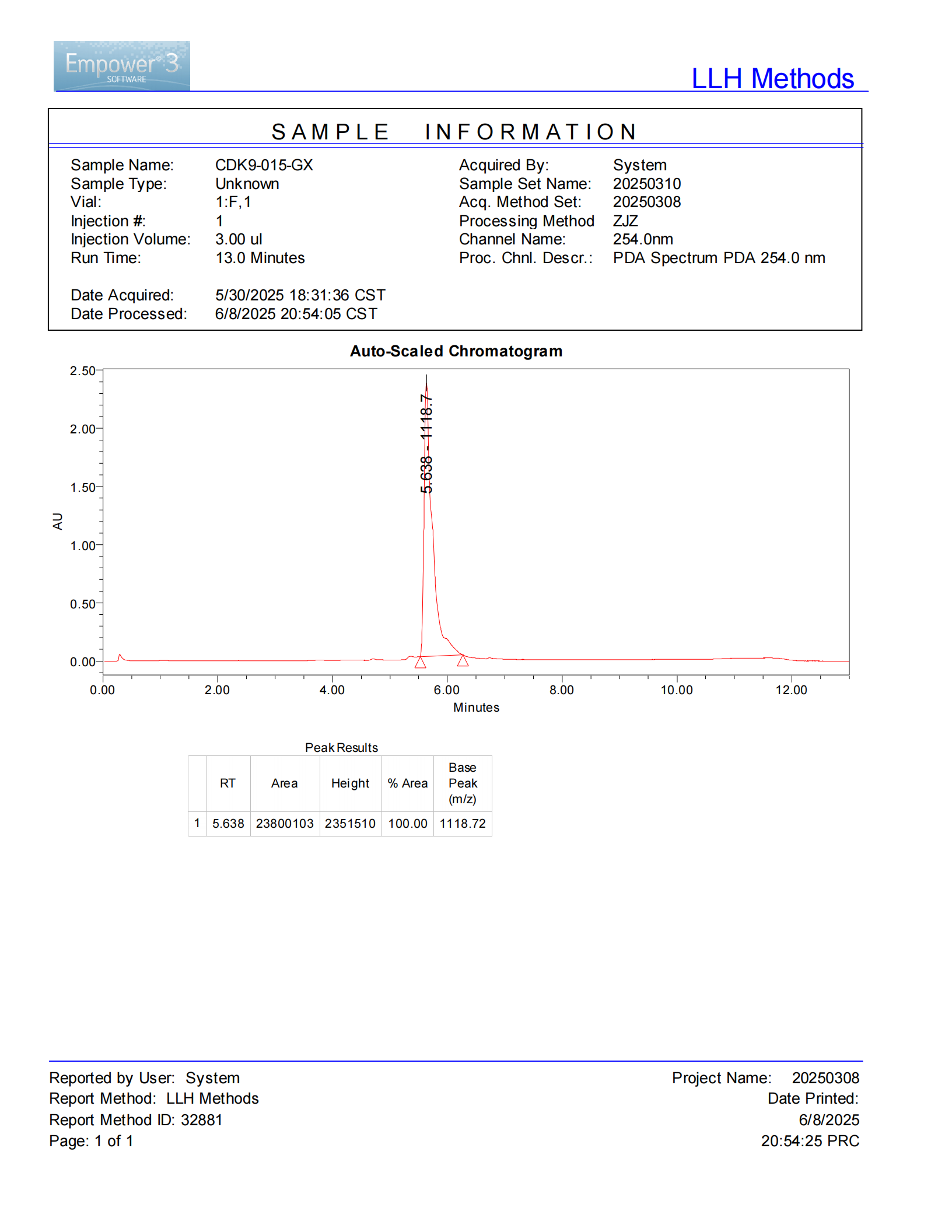

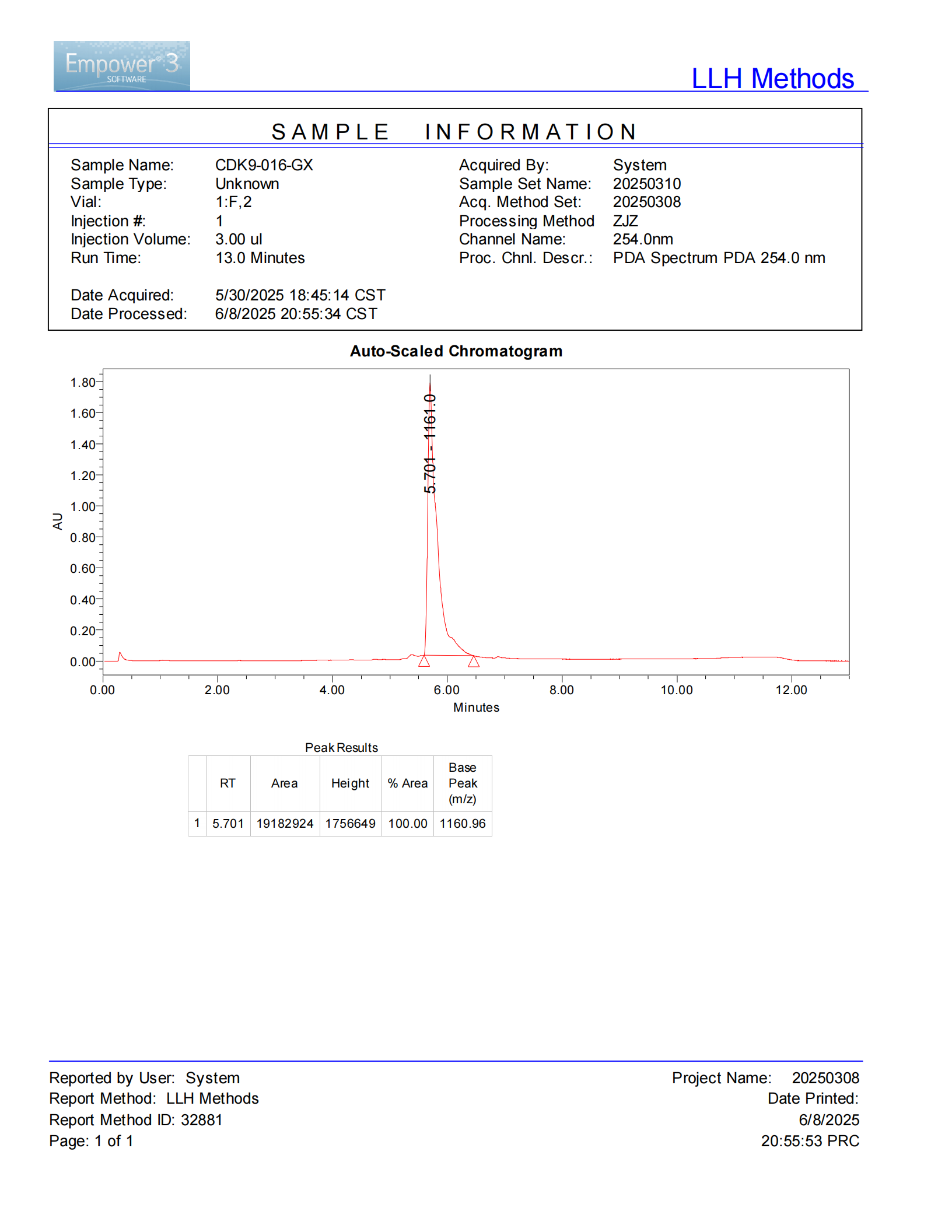

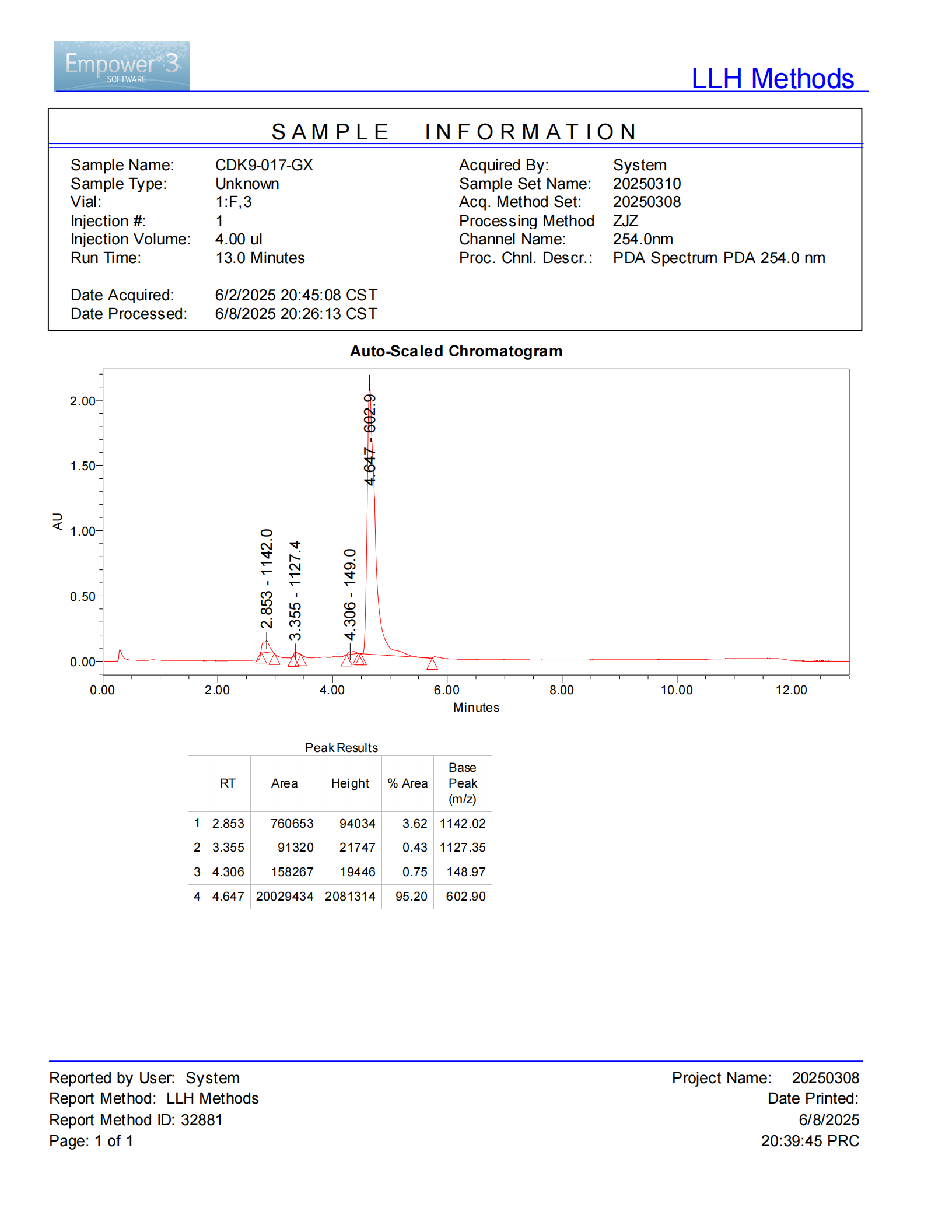

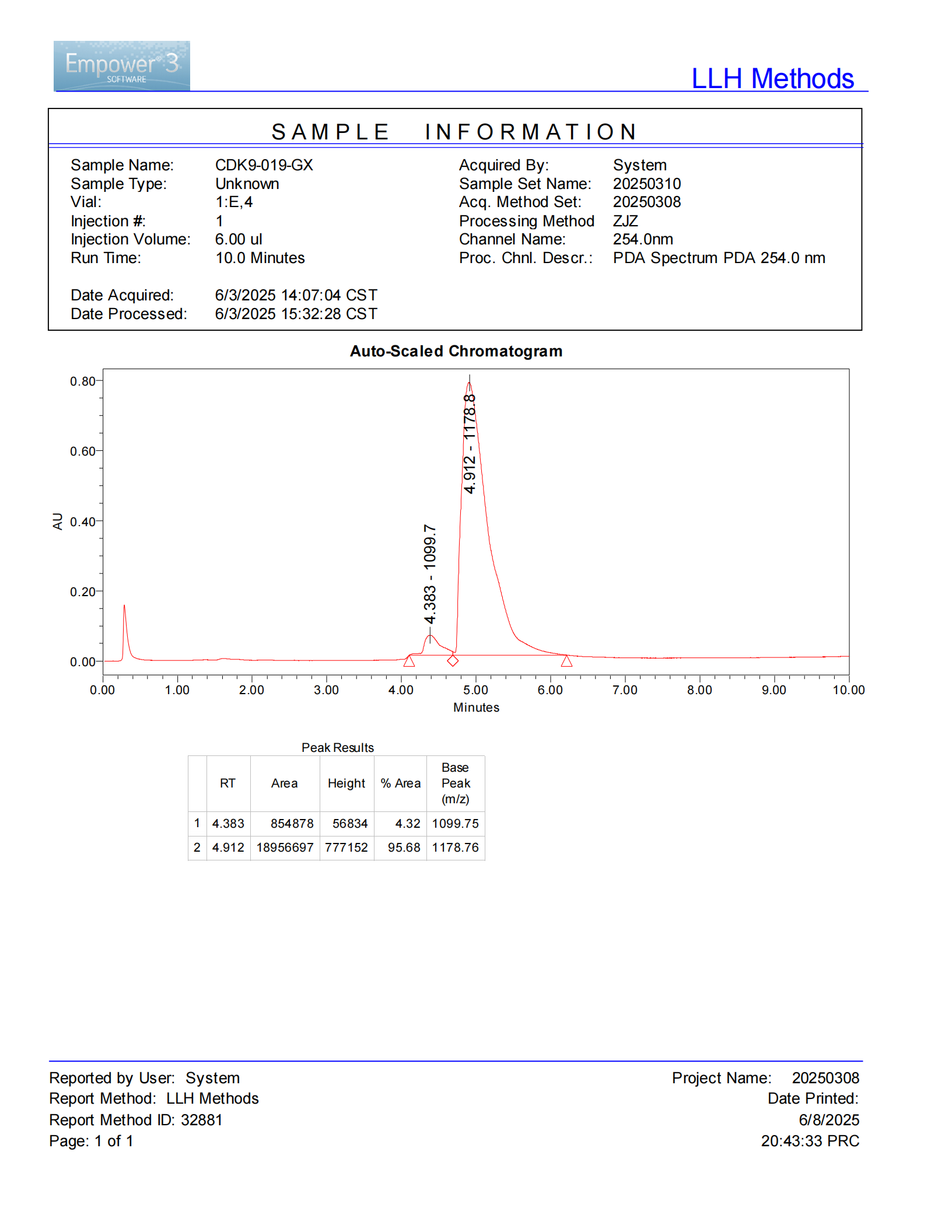

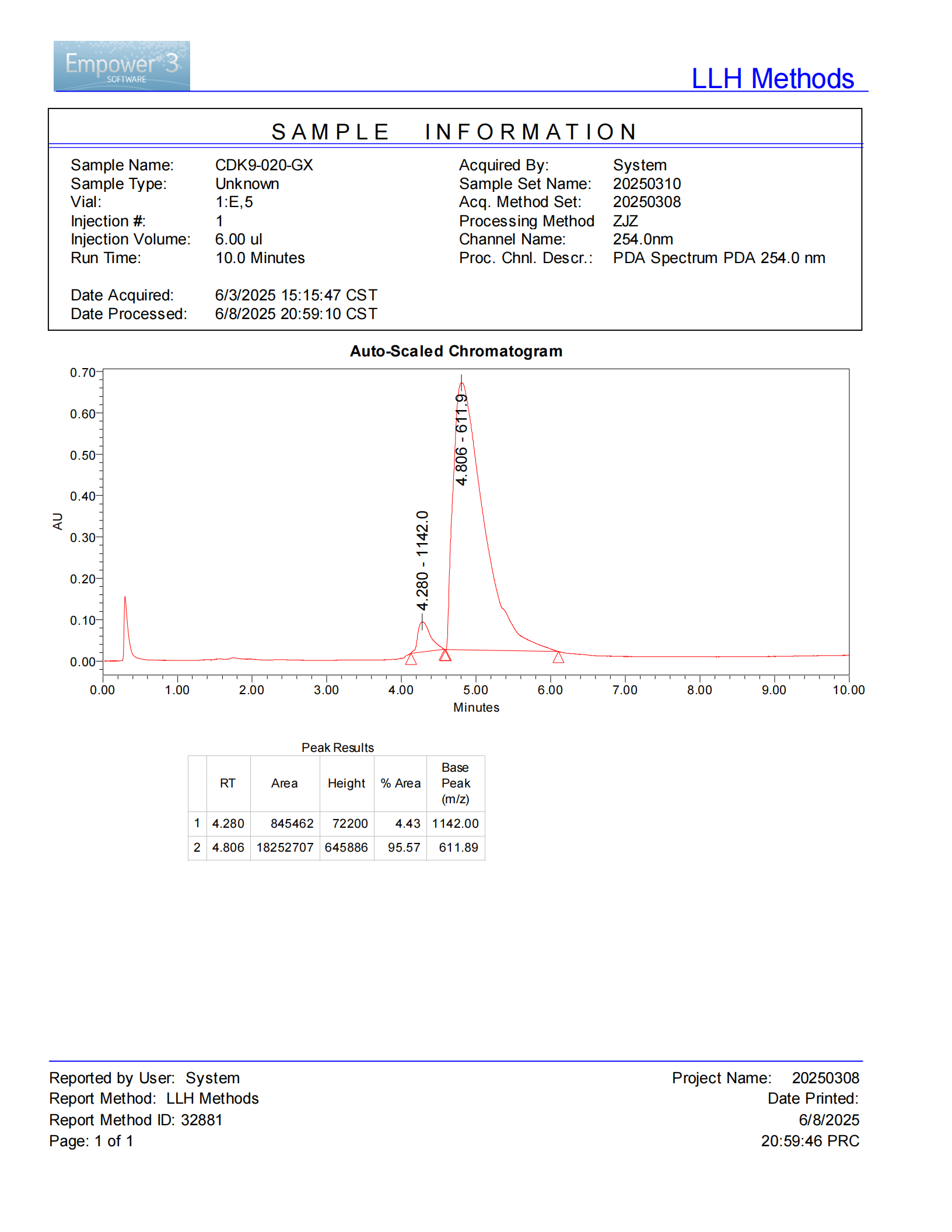

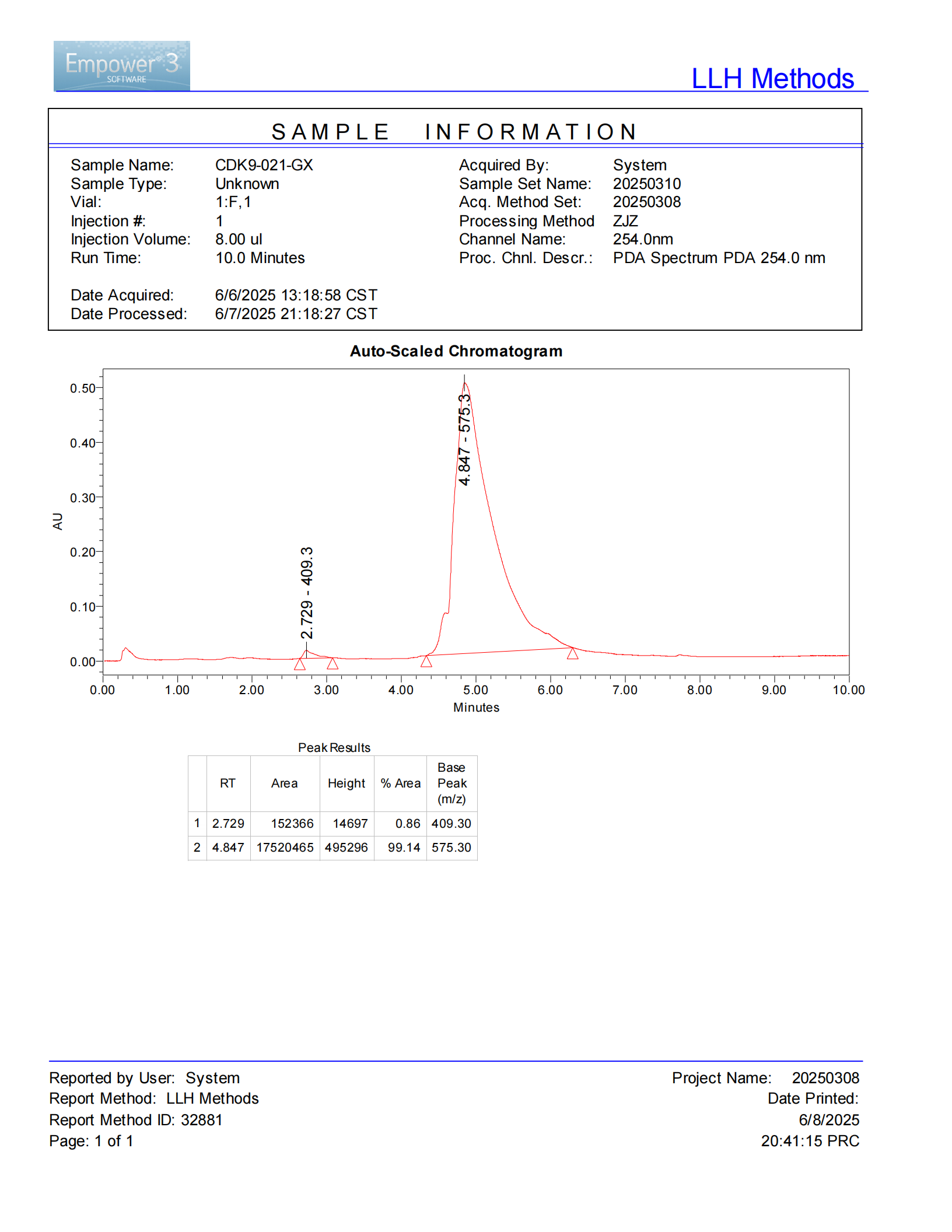

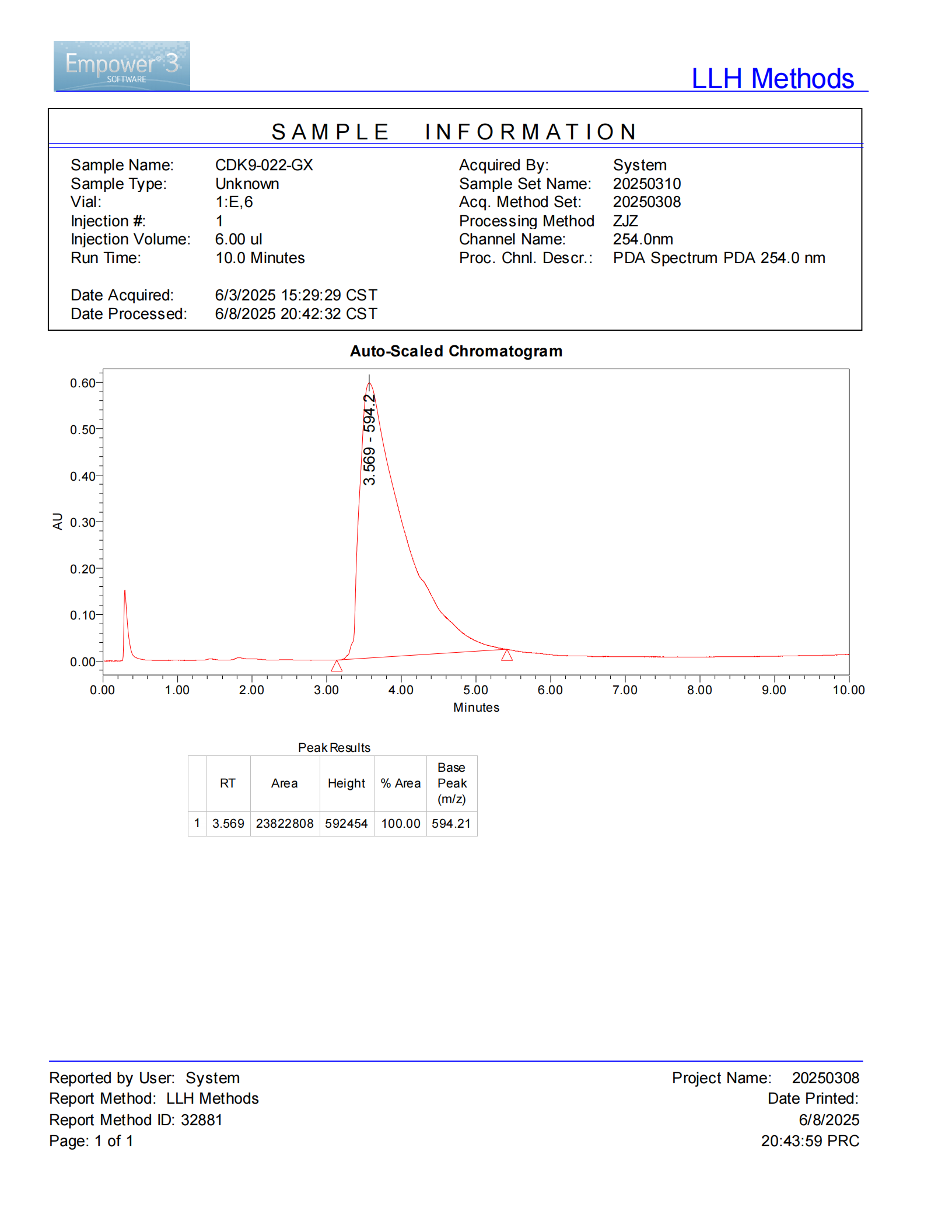

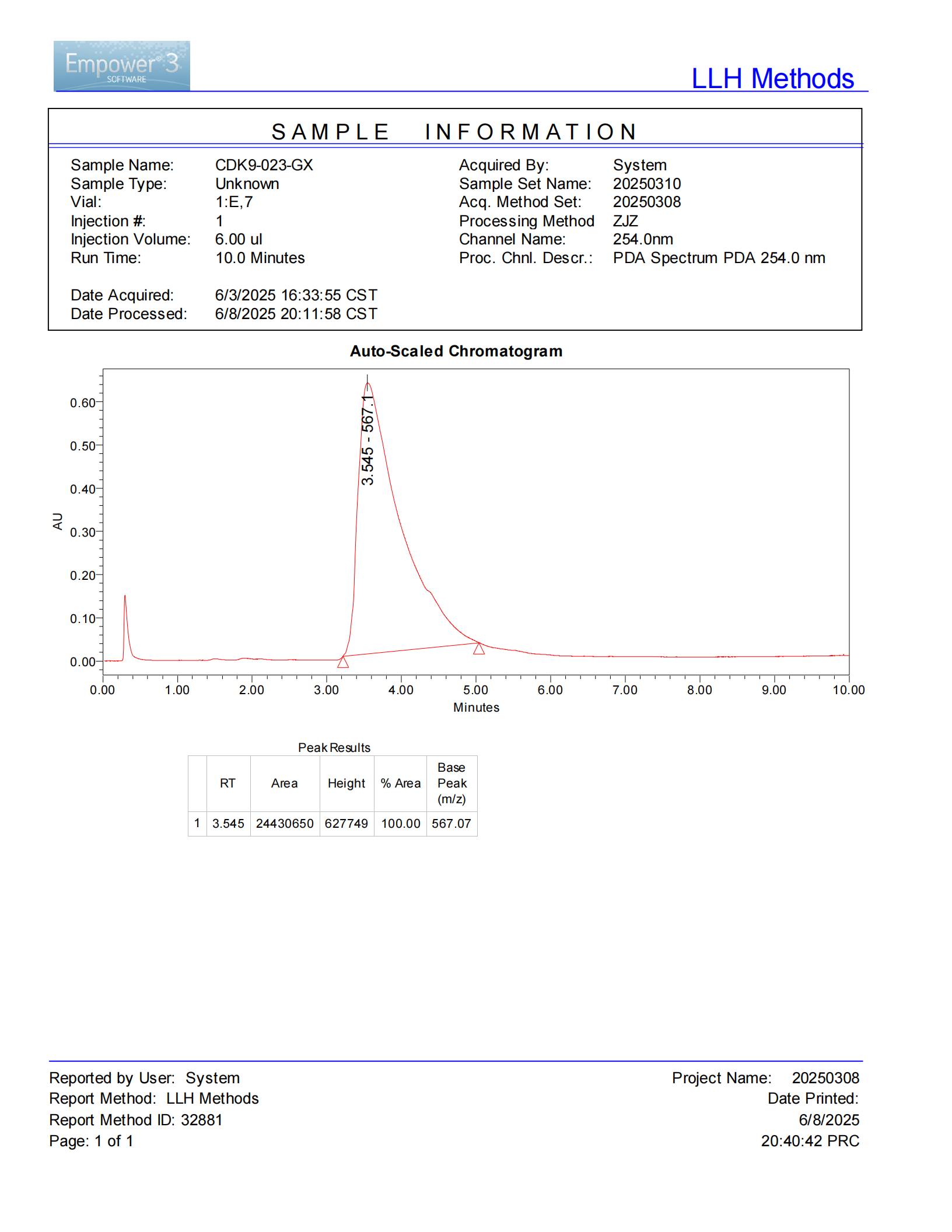

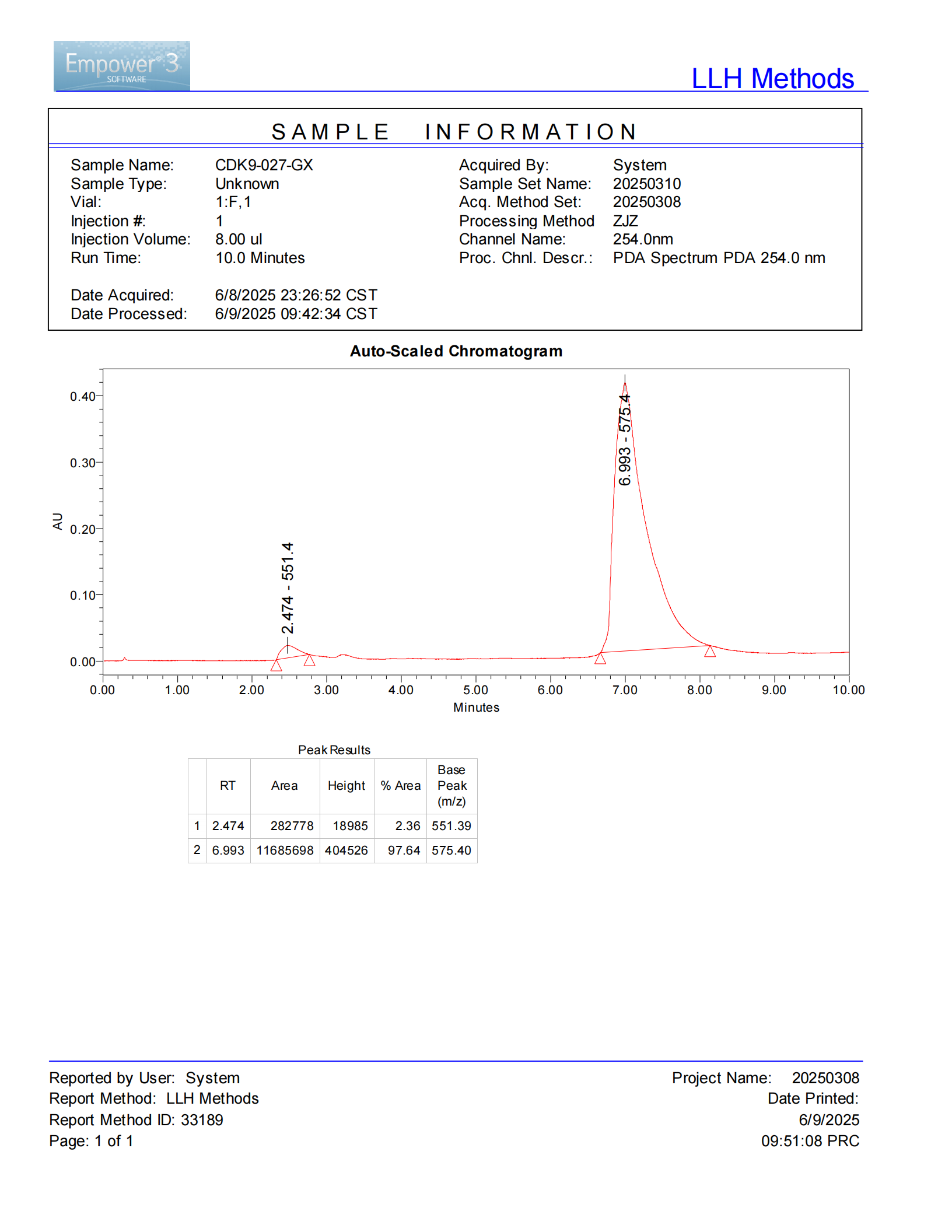
**

## Part 4. HRMS spectra

**HRMS spectra of INT 31**

**HRMS spectra of INT 57**

**HRMS spectra of INT 66**

**HRMS spectra of 4 (dCDK9-001)**

**HRMS spectra of 5 (dCDK9-002)**

**HRMS spectra of 6 (dCDK9-003)**

**HRMS spectra of 7 (dCDK9-004)**

**HRMS spectra of 8 (dCDK9-005)**

**HRMS spectra of 9 (dCDK9-006)**

**HRMS spectra of 10 (dCDK9-007)**

**HRMS spectra of 11 (dCDK9-008)**

**HRMS spectra of 12 (dCDK9-009)**

**HRMS spectra of 13 (dCDK9-010)**

**HRMS spectra of 14 (dCDK9-012)**

**HRMS spectra of 15 (dCDK9-015)**

**HRMS spectra of 16 (dCDK9-016)**

**HRMS spectra of 17 (dCDK9-017)**

**HRMS spectra of 18 (dCDK9-019)**

**HRMS spectra of 19 (dCDK9-020)**

**HRMS spectra of 20 (dCDK9-021)**

**HRMS spectra of 21 (dCDK9-022)**

**HRMS spectra of 22 (dCDK9-023)**

**HRMS spectra of 23 (dCDK9-027)**
